# Visible-light-mediated catalyst-free synthesis of unnatural α-amino acids and peptide macrocycles

**DOI:** 10.1038/s41467-021-27086-x

**Published:** 2021-11-25

**Authors:** Mengran Wang, Chao Wang, Yumei Huo, Xiaobo Dang, Hongxiang Xue, Liangyu Liu, Hongli Chai, Xiuling Xie, Zhixuan Li, Doudou Lu, Zhaoqing Xu

**Affiliations:** 1 Key Laboratory of Preclinical Study for New Drugs of Gansu Province, School of Basic Medical Science, Lanzhou University, Lanzhou, 730000 China; 2 Institute of Biochemistry and Molecular Biology, School of Life Sciences, Lanzhou University, Lanzhou, 730000 China; 3 School of Pharmacy, Lanzhou University, Lanzhou, 730000 China; 4 Research Unit of Peptide Science, Chinese Academy of Medical Sciences, 2019RU066, Lanzhou, 730000 China; 5 Key Laboratory of Dental Maxillofacial Reconstruction and Biological Intelligence Manufacturing, Gansu Province, Lanzhou University, Lanzhou, 730000 China

**Keywords:** Photochemistry, Synthetic chemistry methodology

## Abstract

The visible light induced, photocatalysts or photoabsorbing EDA complexes mediated cleavage of pyridinium C-N bond were reported in the past years. Here, we report an ionic compound promote homolytic cleavage of pyridinium C-N bond by exploiting the photonic energy from visible light. This finding is successfully applied in deaminative hydroalkylation of a series of alkenes including naturally occurring dehydroalanine, which provides an efficient way to prepare β-alkyl substituted unnatural amino acids under mild and photocatalyst-free conditions. Importantly, by using this protocol, the deaminative cyclization of peptide backbone N-terminals is realized. Furthermore, the use of Et_3_N or PPh_3_ as reductants and H_2_O as hydrogen atom source is a practical advantage. We anticipate that our protocol will be useful in peptide synthesis and modern peptide drug discovery.

## Abstract (web summary)

To address the shortcomings in the application of bioactive peptides as drugs, incorporation of unnatural amino acids (UAAs) has been used. Here, the authors report an ionic compound-promoted C-N cleavage of alkyl pyridinium to generate alkyl radicals upon excitation by visible light, and apply it for deaminative hydroalkylation of alkenes to synthesise diverse β-alkyl substituted UAAs.

## Introduction

Peptides are indispensable bioactive components for exerting biological function normally in various cells. Thus, therapeutic peptide is deemed to be an exquisite substitution of an endogenous molecule which possesses high affinity and selectivity against a pharmacologically diverse set of biological targets^1,2^. Although remarkable achievements have been made in peptide research, poor membrane permeability, low metabolic resistance, and bioavailability still are insurmountable barriers to endogenous bioactive peptide therapeutic^3^. In this context, several methods have been developed and applied to solve these problems, such as incorporation of unnatural amino acids (UAAs) or macrocyclizations of linear peptides^4^.

In recent years, with the rapid development of photochemistry, visible light promoted radical coupling reactions have become important pathway for building chemical bonds^5^. Owing to mild reaction conditions and excellent functional group tolerance, photoinduced chemical transformations provided an excellent strategy for chemoselective biomolecule modification, which are widely applied in the modification of amino acids, peptides, and proteins^6–12^. The modification of amino acids is an important strategy for preparing UAAs, such as the modification of glycine, cysteine, tyrosine, tryptophan, histidine, etc. Dehydroalanine (Dha) is a naturally occurring amino acid^13,14^, and also can be easily prepared from Ser, Cys, and selenocystein, which is pre-inserted at the position of interest in peptides and proteins^15,16^. In recent years, Dha has been used as a versatile backbone for synthesis of UAAs. Although these methods provided efficient ways for preparation of various UAAs, the accessing of β-alkyl substituted UAAs were still limited, and the reactions required either transition metal catalysts or stoichiometric amount of metal reagents^17–32^. Furthermore, the compatible methods for modification of Dha unit in peptides are still rare^33,34^, and the related peptide cyclization protocol based on functionalization of Dha is still underdeveloped.

**Fig. 1:**
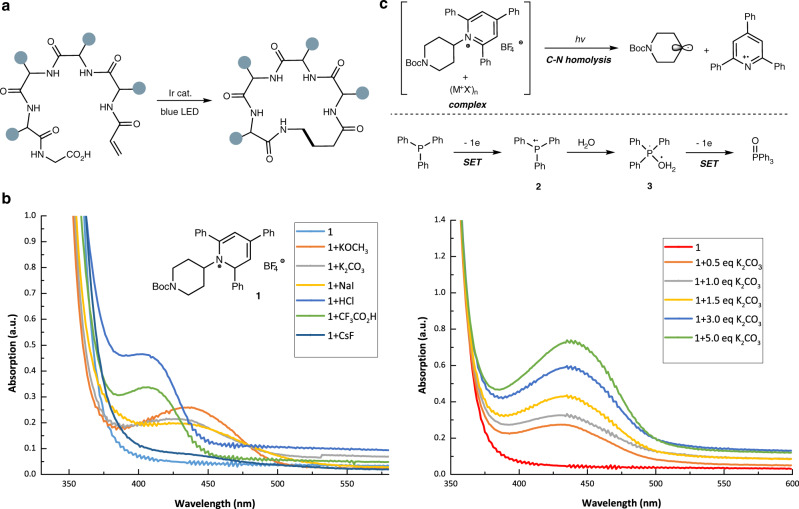
Design plan. **a** Photoredox decarboxylative macrocyclization. **b** UV-Vis absorption spectrum of **1** with ionic compounds. **c** Visible light induced ionic compound promoted homolytic fragmentation of pyridinium C–N bond.

The traditional peptide cyclization methods are based on lactamization and disulfide bond formation. In the past decades, with the advent of new generations of peptide pharmaceuticals, varying the nature of ring-forming linkage in peptide macrocycles became a necessity^35^. In this context, transition metal-catalyzed peptide macrocyclization strategies are gaining increasing popularity, and include C–H activation, oxidative cross-couplings, heteroatom ligation, and radical reactions, etc^36–39^. MacMillan and co-workers recently reported a visible-light-promoted decarboxylative Giese reaction, which provided an efficient way for macrocyclization of peptide backbone *C*-terminal in an alternative manner besides lactam bond (Fig. 1a)^40,41^.

Primary amines are naturally occurring and chemically diverse starting materials, and deaminative reaction of primary amines has emerged as an important strategy for generating alkyl radicals. Katritzky salts derived from α-1° and α-2° amines, as well as imines derived from sterically encumbered α-3° primary amines are important alkyl radical precusors^42,43^. In line with our interests in photocatalysis and peptide synthesis^44–46^, we here report a visible light induced, ionic compounds promoted C–N cleavage of Katritzky salts, which is successfully used in the preparation of β-alkyl substituted UAAs and macrocyclization of peptide. Notably, Et_3_N and PPh_3_ are effective to act as single-electron reductants for the reactions.

## Results

### Design plan

The Katritzky-type alkyl substituted pyridinium (**1**) can generate an alkyl radical through a photoinduced dissociative electron transfer^47^. However, the light absorption of alkyl pyridinium **1** is <400 nm wavelength. To exploit the photonic energy from visible light (wavelength > 400 nm) for cleavage of pyridinium C–N bond, photocatalysts^48^ or photoabsorbing EDA complexes are essentially required^44,49–53^. For example, Aggarwal and co-workers recently reported a visible light induced Giese-type alkylation reaction, in which pyridinium and Hantzsch esters in situ formed EDA complex and mediated the photoredox process^52^. Interestingly, in our recent studies, we found that some ionic compounds (M^+^X^-^), such as K_2_CO_3_, KOCH_3_, NaI, HCl, CF_3_CO_2_H, CsF, etc., could dramatically increase the absorption of **1** in visible light (400-480 nm) region (Fig. 1b, see the “Supplementary information” for details). The loading amount of ionic compound is directly proportional to the light absorption intensity, indicating the key role of ionic additives for increasing the visible light absorption (The reason that ionic additives increasing visible light absorption of Katritzky salt was unclear at this stage. The mechanistic studies and further applications are underway in our lab).

These findings let us wonder whether visible light can be directly used for homolytic alkyl pyridinium (**1**) C–N bond without assistance of photosensitizers by addition of ionic compounds. If so, upon irradiation, C–N homolysis of **1** can promote the release of alkyl radical. Compared with reported EDA complexes triggered SET to alkyl pyridinium, which were highly dependent on a suitable electron donor (e.g., indoles, Hantzsch esters, or aryl amines) to form a light-absorbing aggregator^44,49–53^, these ionic compounds promoted photocatalytic C–N homolysis would unveil more rich photochemistry.

To validate our proposal in Fig. 1c, a suitable single-electron reductant (SER) is required. Tertiary amines are previously widely used as SERs. Besides tertiary amines, we speculated that electron-rich tertiary phosphines, such as PPh_3_, could also be used in our reaction for electron transfer. We assume phosphine could provide one electron to pyridinium radical cation and form a phosphine radical cation **2**, which would then react with H_2_O to give the intermediate **3**. The latter could act as an SER again by supplying a second electron and yield phosphine oxide. Overall, the proposed photochemical mechanism to generate C(sp^3^)-centered radicals by using phosphine and H_2_O as SER was not realized before^54^.

### Investigation of reaction conditions

For our initial explorations (Table 1), we selected N-Ac-Dha methyl ester (**4a**) (0.1 mmol) as Michael acceptor and N-Boc-protected cyclic pyridinium salt **1** as alkyl radical precursor. Most of the tested tertiary alkyl amines and trivalent tertiary phosphines could serve as SER for the reaction, while Et_3_N (entry 1) and PPh_3_ (entry 2) gave the best results, respectively. Notably, in the case when PPh_3_ was used, stoichiometric amount of triphenylphosphine oxide (Ph_3_PO) was formed as a byproduct, indicating that PPh_3_ and H_2_O were all involved in SET process. **5a** was obtained in 50% yield in the absence of K_2_CO_3_ under condition A, which might be attributed to the formation of the EDA complex between Katritzky salt **1** and Et_3_N (entry 3)^52^. It is worth noting that the yield of **5a** dramatically increased to 97% in the presence of K_2_CO_3_ (entry 1). The yield of **5a** decreased to 13% in the absence of KOCH_3_ under condition B (entry 4). In addition to K_2_CO_3_ and KOCH_3_, fluoride additives could also increase the outcome (entries 5 and 6, respectively). Without Et_3_N or PPh_3_, only a trace amount of desired product formed (entry 7). The reaction gave diminished yield in the absence of H_2_O (entry 8). We assumed that H_2_O might play two roles in the reaction. One is increasing the solubility of an ionic compound; the other is involving in the SET process when PPh_3_ is used as an SER reagent (Fig. 1c). Without light irradiation, the reaction did not proceed at room temperature or 60 °C(entries 9 and 10, respectively).

**Table 1:** Control experiments.


Entry	Changes from condition A or B	Yield of 5a (%)^a^
1	no change from condition A	97% (94%)
2	no change from condition B	88% (84%)
3	no K_2_CO_3_ under condition A	50%
4	no KOCH_3_ under condition B	13%
5	KF instead of K_2_CO_3_ under condition A	63%
6	CsF instead of KOCH_3_ under condition B	41%
7	without Et_3_N or PPh_3_	trace
8	without H_2_O	<54%
9	no light	N.D.
10	no light, 60 ^ o^C	N.D.

^a^Yield was determined by ^1^H NMR using 4-bromobenzaldehyde as an internal standard. The value within parentheses refers to isolated yield.

### Substrate scopes

**Fig. 2:**
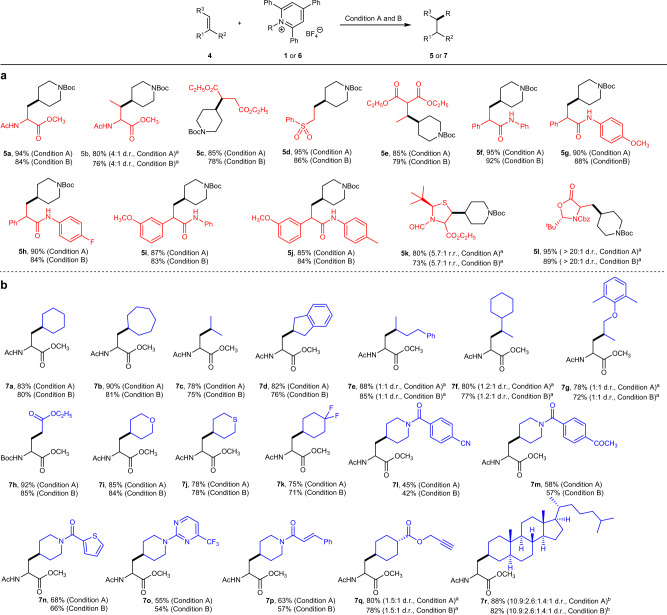
Investigation of reaction substrate scope with respect to the alkenes and Katritzky salts. Reaction condition A: alkene (0.2 mmol), Katritzky salt (0.5 mmol, 2.5 equiv), Et_3_N (0.4 mmol, 2.0 equiv), K_2_CO_3_ (0.2 mmol, 1.0 equiv), and H_2_O (6.0 mmol. 30 equiv) in CH_3_CN (3.0 mL), violet LED (24 W, 410-420 nm), argon atmosphere, 12 h, room temperature. Reaction condition B: alkene (0.2 mmol), Katritzky salt (0.4 mmol, 2.0 equiv), PPh_3_ (0.48 mmol, 2.4 equiv), KOCH_3_ (0.6 mmol, 3.0 equiv), and H_2_O (5.0 mmol. 25 equiv) in acetone (3.0 mL), violet LED (24 W, 420–430 nm), argon atmosphere, 12 h, room temperature. Isolated yields after chromatographic purification. ^a^Regio- or diastereomers were measured by^1^H NMR. ^b^Diastereomers were measured by HPLC. **a** Substrates with alkenes (Condition A and B). **b** Substrates with Katritzky salts (Condition A and Condition B).

Having established the optimal reaction conditions (Table 1, entries 1 and 2), we examined a series of alkenes (**4b**–**4l**) as shown in Fig. 2a. Similar to Dha, another naturally occurring dehydroamino acid derived alkene, namely dehydrobutyrine, also reacted smoothly to give the corresponding product **5b** in 80% yield (4:1 d.r.) under condition A and 76% yield (4:1 d.r.) under condition B. Other alkenes, such as diethyl fumarate, vinyl sulfone, or dimethyl malonate derived alkene were all compatible in the reaction with good yields (**5c**–**5e**, 78–95%). The Michael acceptors bearing amide groups also worked well under optimal conditions to give the desired products in uniformly good yields (**5f**–**5j**). Furthermore, we expanded the reaction scope to β-thiolated amino acid derivative (**4k**) which was readily prepared from *L*-cysteine and dehydroalanine derived Karady-Beckwith alkene (**4l**). The corresponding hydroalkylation products **5k** and **5l** were obtained in high yields and good r.r. (rotamers mixture ratio)^55^ or d.r.

Subsequently, we shifted our attention to the scope of Katritzky salts for the synthesis of unnatural α-amino acids using a series of secondary alkyl substituted pyridinium (Fig. 2b). Uniformly good yields were obtained (**7a**–**7h**, up to 92%). Importantly, this reaction could be extended to glycine derived Katritzky salt (**7h**, 92 and 85%), which provided the possibility for deaminative N-terminal macrocyclization of the linear peptide backbone. Furthermore, more complex pyridinium radical precursors bearing various functional groups and structural motifs were tested under condition A and B, which provided corresponding products **7i**–**7r** in moderate to excellent yields. Unfortunately, unactivated primary alkyl substrates had failed to give the desired hydroalkylation products (When the unactivated primary alkyl substrate was used in the reaction, significant amounts of byproduct from radical–radical coupling of primary alkyl dihydropyridine radical with unactivated primary alkyl radical was obtained. See supplementary information for details.).

**Fig. 3:**
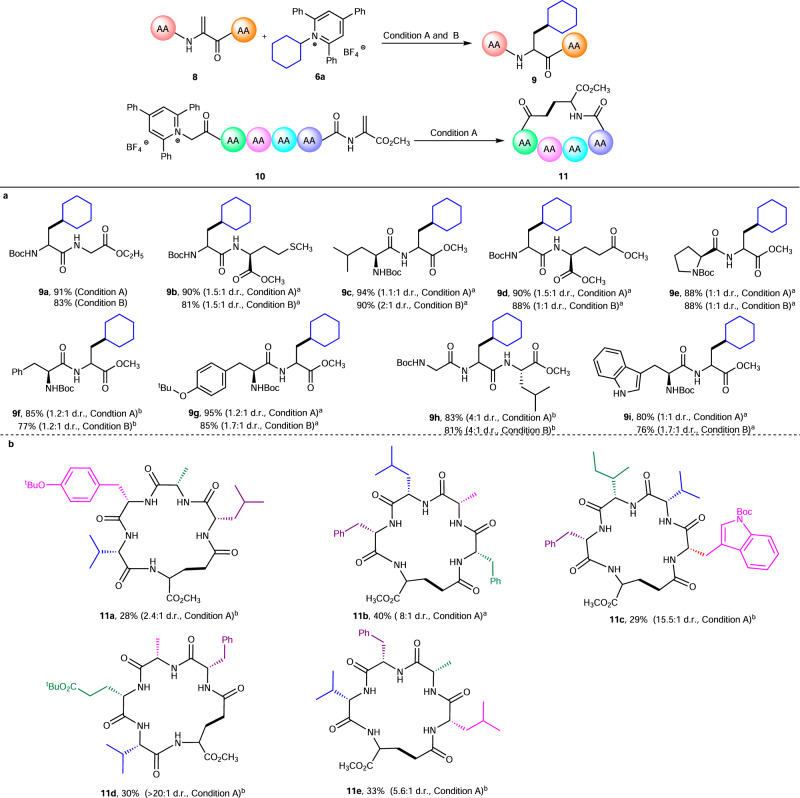
Modification and macrocyclization of peptides. ^a^Diastereomers were measured by ^1^H NMR. ^b^Diastereomers were measured by HPLC. **a** Isolated yield on 0.2 mmol scale (condition A and condition B). **b** For **11a** and **11c**–**11e**, isolated yields on 0.02 mmol under condition A; for **11b**, isolated yield on 0.05 mmol under condition A.

Next, a variety of Dha containing peptides was prepared to test the functional group tolerance of this reaction with various amino acid residues incorporated. To our delight, peptides bearing glycine, proline, leucine, tryptophan, phenylalanine, or tyrosine units were able to participate in the deaminative conjugation to furnish the desired adducts in yields between 76 and 95% with other amino acid residues untouched (Fig. 3a, **9a**–**9i**). Based on excellent performance on modification of peptides, we turned our attention to deaminative N-terminals macrocyclization of linear peptides. As shown in Fig. 3b, a series of peptides that incorporate a structurally diverse set of amino acids can be successfully cyclized using this deaminative method (**11a**–**11e**, 28–40% isolated yields after HPLC purifications). Regrettably, the attempt to macrocyclize Dha contained peptide “on resin” during solid-phase peptide synthesis procedure has failed.

### Mechanistic studies

**Fig. 4:**
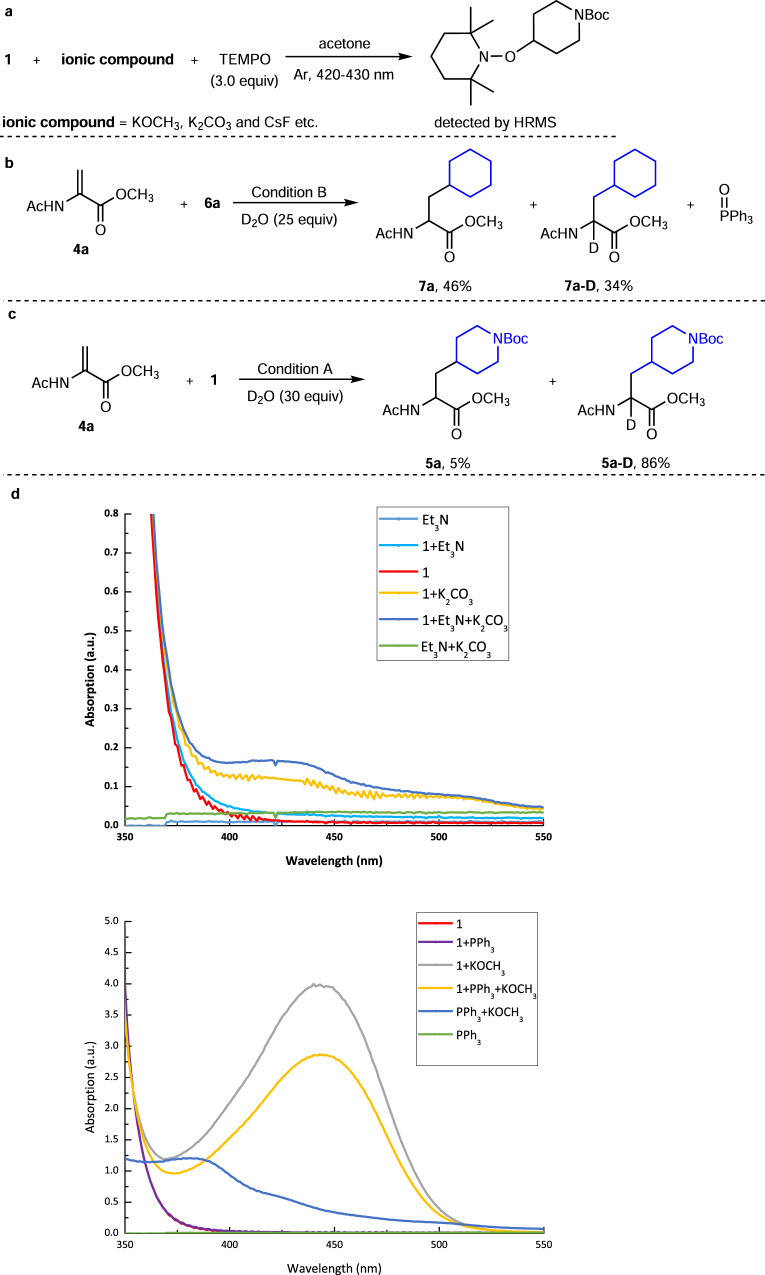
Mechanistic studies. **a** Radical trapping experiments. **b** Isotope tracking experiments of Condition B. **c** Isotope tracking experiments of Condition A. **d** Analysis of UV-Vis absorption spectra.

To gain some detailed information for the reaction, a series of mechanistic studies were carried out (see Supplementary information for details). As shown in Fig. 4a, radical trapping experiments indicated that the combination of ionic compounds (such as K_2_CO_3_, KOCH_3_, and CsF) and pyridinium were able to harness photonic energy from visible light and promoted C–N homolysis of Katritzky salt to generate an alkyl radical. In isotope tracking experiments (Fig. 4b, c), when D_2_O was used instead of H_2_O under Condition A and B, deuterated product **5a–D** and **7a–D** were obtained in 86 and 34% yield, respectively, revealing that H_2_O acted as hydrogen atom source to provide an H^+^ for the hydroalkylation process. Furthermore, stoichiometric amount of triphenylphosphine oxide (Ph_3_PO) was formed as the byproduct, which suggested that PPh_3_ could possibly provide two electrons in a stepwise way during the SET process (Fig. 4b). The UV-Vis absorption of individual reagents or mixtures were shown in Fig. 4d. None of Et_3_N, PPh_3_, pyridinium (**1**), or the mixture of [PPh_3_ + **1**] showed significant absorption over 400 nm. However, basic ionic compounds, namely K_2_CO_3_ and KOCH_3_, could dramatically increase the absorption of pyridinium (**1**) in the visible light region, respectively. Interestingly, the combination [Et_3_N + **1**] showed a slight bathochromic shift, which might be due to the formation of EDA complex between **1** and Et_3_N^52^. Although this EDA complex could possibly mediate the alkylation process (Table 1, entry 3), the yield was significantly lower than for K_2_CO_3_ promoted reaction (50% vs 97%). Some ionic compounds, such as Cs_2_CO_3_, K_3_PO_4_, and NaOCH_3_, provide higher levels of visible light absorption; however, only moderate yields were obtained (Table S3 and Fig. S8). The use of KBF_4_ and NaBF_4_ as ionic additives did not give any desired product, which indicated that BF_4_^−^ counter anion in Katritzky salt was not crucial for the transformation (Table S3, Table S13, and Fig. S8).

**Fig. 5:**
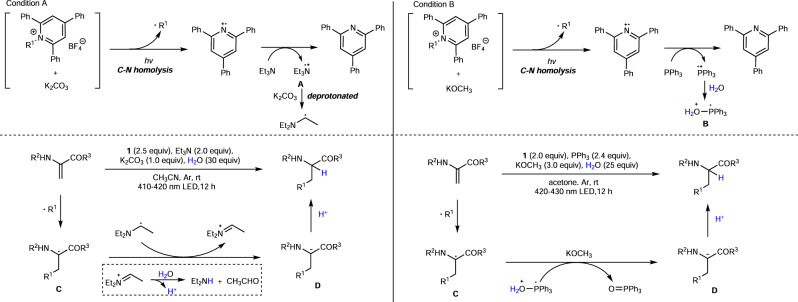
Proposed mechanism. Visible-light-mediated catalyst-free synthesis of unnatural α-amino acids.

On the basis of these preliminary results, a possible mechanism is proposed (Fig. 5) (Although the mechanistic investigations suggested that a visible light promoted C-N homolysis of Katritzky salts in the presence of ionic compounds is highly possible in our reaction, the EDA complex pathway cannot be completely ruled out at this stage. See supplementary information for details.). Initially, C–N homolysis of Katritzky salt occurred in the presence of basic ionic compounds under visible light irradiation, followed by the formation of an alkyl radical (•R^1^) and pyridinium radical cation. Et_3_N (PPh_3_) was oxidized to nitrogen radical cation **A**^52^ (phosphine radical cation) with the formation of 2,4,6-triphenylpyridine. Notably, under condition A, nitrogen radical cation **A** is deprotonated to yield α-amino radical, and under condition B, phosphine radical cation reacted with H_2_O to give intermediate **B**^54^. Radical addition of •R^1^ to Michael acceptor **4** gives radical intermediate **C**. According to the previous report^56^, under condition A, intermediate **C** was reduced by α-amino radical to give the carbon anion species **D** and an immonium ion. Subsequently, the hydrolysis of immonium ion promoted the generation of diethylamine and acetaldehyde, followed by the release of a proton. Under condition B, intermediate **C** was reduced by **B** to form a carbon anion species **D** alone with the formation of triphenylphosphine oxide. The final protonation of **D** provided our desired deaminative hydroalkylation product. In condition B, PPh_3_ served as the single-electron reductant and provided two electrons in a stepwise way.

## Discussion

In summary, we report ionic compounds promoted homolytic cleavage of pyridinium C–N bond by exploiting the photonic energy from visible light. This finding was successfully applied in deaminative hydroalkylation of a series of alkenes including naturally occurring Dha, which provided an efficient way to prepare β-alkyl substituted UAAs under mild and catalyst-free conditions. Importantly, by using this protocol, the deaminative cyclization of peptide backbone N-terminals was realized. Furthermore, the use of Et_3_N or PPh_3_ as reductants and H_2_O as hydrogen atom source is a significantly practical advantage. Further investigating the mechanism of ionic compounds improved visible light absorption of pyridinium and other synthetic applications are currently underway and will be reported in due course.

## Methods

### General procedure of condition A

To an oven-dried 10 mL quartz test tube with a stirring bar was added alkene (0.2 mmol), alkyl pyridinium salt (0.5 mmol, 2.5 equiv), Et_3_N (0.4 mmol, 2 equiv), and K_2_CO_3_ (0.2 mmol, 1 equiv). Then, the air was withdrawn and backfilled with Ar (three times). CH_3_CN (3 mL) and H_2_O (6 mmol, 30 equiv) were added. The mixture was transferred to a violet LED photoreactor (24-W, 410–420 nm), where it was irradiated for 12 h. Then, the reaction was quenched with water (5 mL), extracted with ethyl acetate, washed with brine, dried over anhydrous sodium sulfate, concentrated in vacuo, and purified by column chromatography (hexane/acetone) to afford the product.

### General procedure of condition B

To an oven-dried 10 mL quartz test tube with a stirring bar was added alkene (0.2 mmol), alkyl pyridinium salt (0.4 mmol, 2.0 equiv), PPh_3_ (0.48 mmol, 2.4 equiv), and KOCH_3_ (0.6 mmol, 3.0 equiv). Then, the air was withdrawn and backfilled with Ar (three times). Acetone (3 mL) and H_2_O (5 mmol, 25 equiv) were added. The mixture was transferred to a violet LED photoreactor (24-W, 420–430 nm), where it was irradiated for 12 h. Then, the reaction was quenched with water (5 mL), extracted with ethyl acetate, washed with brine, dried over anhydrous sodium sulfate, concentrated in vacuo, and purified by column chromatography (hexane/acetone) to afford the product.

## Supplementary information

Supplementary information

## Data Availability

The authors declare that the data supporting the findings of this study, including experimental details and compound characterization, are available within the article and its Supplementary information file. All data are available from the corresponding author upon request.
