# Supplementary material for: Visible-light-mediated catalyst-free synthesis of unnatural α-amino acids and peptide macrocycles
Source: Nat Commun. 2021 Nov 25;12:6873. doi: 10.1038/s41467-021-27086-x (PMC8617070; doi:10.1038/s41467-021-27086-x)
Supplement: Supplementary file 1 — Supplementary information [file 41467_2021_27086_MOESM1_ESM.pdf]

Supplementary Information

Visible-Light-Mediated Catalyst-Free Synthesis of Unnatural α-Amino Acids and Peptide Macrocycles

Mengran Wang,† Chao Wang,† Yumei Huo, Xiaobo Dang, Hongxiang Xue, Liangyu Liu, Hongli Chai, Xiuling Xie, Zhixuan Li, Doudou Lu, Zhaoqing Xu*

†These authors contributed equally to this work.

*correspondence to: zqxu@lzu.edu.cn

Table of Contents

1. General information -- 2
2. The synthesis of substrates --- 2
3. General procedures --- 8
4. Characterization of products --17
5. The mechanistic studies --33
6. NMR spectra of products --41
7. Supplementary References ---87

1. General information

All commercially available reagents were used without further purification unless otherwise stated. All solvents were purified and dried according to standard methods prior to use. α-amino acids, Et_3N, PPh_3 (triphenylphosphine), and $KOCH_3$ (potassium methoxide) were purchased from Energy Chem. ^1H NMR, ^{19}F NMR, and ^{13}C NMR spectra were recorded on a Bruker 300 instrument spectrometer in $CDCl_3$ unless otherwise noted. Data for ^1H NMR are recorded as follows: chemical shift (δ, ppm), multiplicity (s = singlet, d = doublet, t = triplet, m = multiplet, q = quartet, dd = doublet of doublets, dt = doublet of triplets, td = triplet of doublets, and br = broad signal, and coupling constant(s) in Hz integration). Data for ^{13}C NMR and ^{19}F NMR are reported in terms of chemical shift (δ, ppm). The NMR spectra of **11b** was recorded on 600MHz Liquid State NMR Spectrometer (AVANCE NEO 600) in CD_3OD. Reactions were monitored by thin layer chromatography (TLC) and column chromatography purifications were carried out using silica gel. Melting points were measured on a SCW X-4 and values are uncorrected. UV-Vis absorption spectra were recorded by using BIOMATE 3S UV-Visible Spectrophotometer. All new compounds were further characterized by high resolution mass spectra (HRMS, ESI source). Semi preparative HPLC was performed on HANBON NP7005C. HPLC analysis were performed on HANBON NP7001C. HPLC was performed on Waters 1525 Binary HPLC, using PHENOMENEX AD-H and CHIRALCEL IC chiral column, eluted with a mixture of hexane and ethanol.

2. The synthesis of substrates

Alkyl pyridinium salts[1-4], dipeptides[5,6], alkenes **4b**[7], **4f-4j**[8], and **4k**[9] were all prepared according to previous reports.

General Procedure A:

The alkyl amine (1.2 equiv) was added to a suspension of 2,4,6-triphenylpyrylium tetrafluoroborate (1.0 equiv) and EtOH (1.0 M) in a round-bottomed flask. The flask was fitted with a reflux condenser and the mixture was stirred at reflux (80-85 ºC) for 4 h. The mixture was then allowed to cool to room temperature. If product precipitation occurred, the solid was filtered, washed with EtOH (3 x 25 mL) and then Et_2O (3 x 25 mL), and dried under high vacuum. If product precipitation did not occur, the solution was diluted with Et_2O (2-3 times volume of EtOH used) and vigorously stirred for 1 h to induce trituration. The resulting solid pyridinium salt was filtered and washed with Et_2O (3 x 25 mL). If the salt still did not precipitate, it was subjected to silica gel chromatography using EtOAc/CH_2Cl_2 as the eluent.

General Procedure B:

The alkyl amine (1.0 equiv) was added to a suspension of 2,4,6-triphenylpyrylium tetrafluoroborate (1.0 equiv), powdered activated 4Å molecular sieves (~500 mg/mmol), and CH_2Cl_2 (0.5 M) in a round-bottomed flask equipped with a stir bar under Ar atmosphere. The flask was fitted with a septum and a vent needle. The mixture was stirred as Et_3N (1.0 equiv for free amines; 2.0 equiv for amine hydrochloride) was added by syringe. The vent needle was removed, and the mixture was stirred at r.t. for 30 min. The vent needle was reinserted before the addition of acetic acid (2.0 equiv). The needle was again removed, and the mixture was stirred at r.t. overnight. The mixture was then filtered through a short pad of Celite using CH_2Cl_2 to rinse the flask and the

Celite pad, and was concentrated under vacuo and purified by silica gel chromatography with $EtOAc/CH_2Cl_2$ as the eluent.

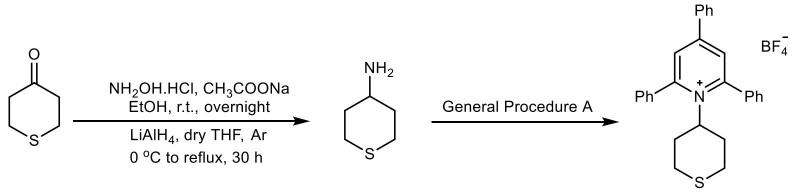

Tetrahydrothiopyran-4-one (2mmol) was dissolved in EtOH (20 mL), then $NH_2OH{\cdot}HCl$ (6 mmol, 3 equiv) and NaOAc (6 mmol, 3 equiv) were added. The mixture was stirred at r.t. overnight. Subsequently, resulting solution was concentrated in vacuo, diluted with CH_2Cl_2 and washed with water. Organic fraction was dried over Na_2SO_4, filtrated and concentrated. The crude oxime was redissolved in dry THF (20 mL), cooled to 0 °C, and $LiAlH_4$ (14 mmol, 7 equiv) was carefully added. The resulting suspension was refluxed for 30 h under Ar atmosphere. Subsequently, the reaction was quenched by dropwise addition of CH_3OH and filtrated. The resulted solution was diluted with CH_2Cl_2 and washed with saturated K_2CO_3. Organic fraction was dried over Na_2SO_4, filtrated and concentrated in vacuo to obtain crude tetrahydro-2H-thiopyran-4-amine. Then, the corresponding Katritzky salt was prepared through general procedure A. **2,4,6-triphenyl-1-(tetrahydro-2H-thiopyran-4-yl)pyridin-1-ium tetrafluoroborate :** ^{1}**H NMR** (300 MHz, $CDCl_3$) δ 7.85-7.43 (m,17H), 4.59 (t, J = 12.3Hz, 1H), 2.51-2.40 (m, 4H), 2.20-2.12 (m, 2H), 1.92-1.80 (m, 2H). **HRMS (ESI)** $C_{28}H_{26}NS^+$ [M-BF_4] calcd: 408.1781, found: 408.1785.

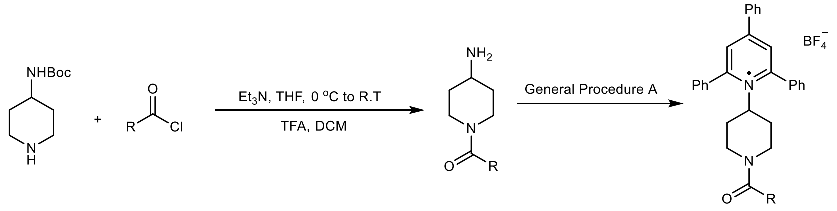

Et_3N (4 mmol, 2 equiv) was added into THF solution of acyl chloride (2 mmol, 1 equiv) at 0 °C, then, 4-Boc-aminopiperidine (2 mmol, 1 equiv) was added slowly. The mixture was stirred at room temperature. The resulting solution was concentrated in *vocuo*. The crude product was dissolved in CH_2Cl_2, and TFA (10 mmol, 5 equiv) was added. Then, the corresponding Katritzky salt was prepared through general procedure A.

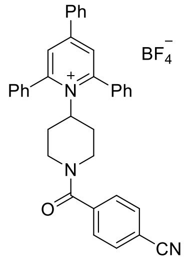

1-(1-(4-cyanobenzoyl)piperidin-4-yl)-2,4,6-triphenylpyridin-1-ium tetrafluoroborate.
^{1}**H NMR** (300 MHz, $CDCl_3$) δ 7.74-7.49 (m,17H), 7.41 (d, J = 6.9 Hz, 2H), 7.10 (d, J = 7.5 Hz, 2H), 4.91-4.87 (m, 1H), 4.49 (br, 1H), 3.36 (br, 1H), 2.49 (br, 1H), 2.23-2.19 (m, 2H), 1.82-1.61 (m, 3H). **HRMS (ESI)** $C_{36}H_{30}N_3O^+$ [M-BF_4] calcd: 520.2384, found: 520.2425.

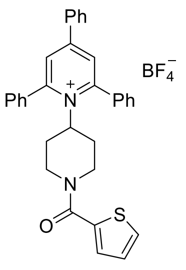

2,4,6-triphenyl-1-(1-(thiophene-2-carbonyl)piperidin-4-yl)pyridin-1-ium tetrafluoroborate.
^{1}H NMR (300 MHz, $CDCl_3$) δ 7.78 (s, 2H), 7.74-7.70 (m, 6H), 7.65-7.58 (m, 6H), 7.55-7.49 (m, 3H), 7.40 (d, *J* = 4.8 Hz, 1H), 6.99-6.94 (m, 2H), 4.93 (t, *J* = 11.1Hz, 1H), 4.28-4.24 (m, 2H), 2.42-2.34 (m, 2H), 2.26-2.22 (m, 2H), 1.81-1.78 (m, 2H). **HRMS (ESI)** $C_{33}H_{29}N_2OS^+$ [M-BF4] calcd: 501.1996, found: 501.2052.

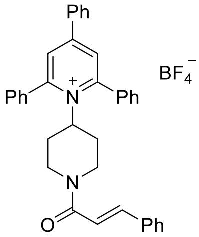

1-(1-cinnamoylpiperidin-4-yl)-2,4,6-triphenylpyridin-1-ium tetrafluoroborate.
^{1}H NMR (300 MHz, $CDCl_3$) δ 7.83 (s, 2H), 7.74-7.70 (m, 6H), 7.64-7.44 (m, 12H), 7.37-7.55 (m, 3H), 6.60(d, *J* = 15.6 Hz, 1H), 4.89 (t, *J* = 11.7Hz, 1H), 4.57-4.55 (m, 1H), 3.96-3.87 (m, 1H), 2.60-2.35 (m, 2H), 2.05 (br, 1H), 1.77-1.69 (m, 3H). **HRMS (ESI)** $C_{37}H_{33}N_2O^+$ [M-BF4] calcd: 521.2588, found: 521.2651.

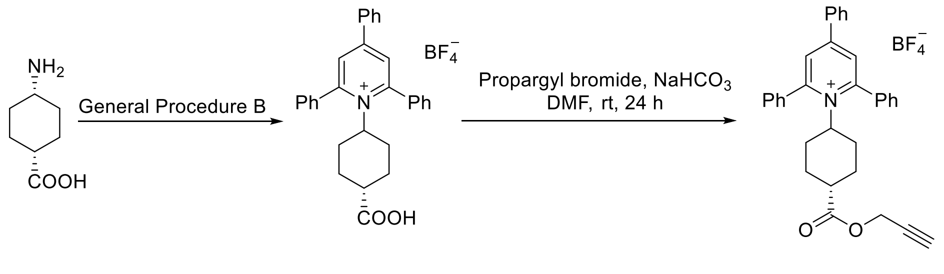

1-(4-carboxycyclohexyl)-2,4,6-triphenylpyridin-1-ium was prepared through procedure B. To a round bottom flask, 1-(4-carboxycyclohexyl)-2,4,6-triphenylpyridin-1-ium (1 mmol, 1 equiv) and $NaHCO_3$ (7.2 mmol, 7.2 equiv) was diluted with dry DMF under Ar atmosphere. The mixture was stirred 10 min at room temperature, propargyl bromide (21 mmol, 21 equiv) was then added. The mixture was stirred 24 h at room temperature. The resulting solution was diluted with EtOAc, and washed with saturated NaCl. Organic fraction was dried over Na_2SO_4, and concentrated in vacuo to give the product. **2,4,6-triphenyl-1-(4-((prop-2-yn-1-yloxy)carbonyl)cyclohexyl)pyridin-1-ium tetrafluoroborate : ^{1}H NMR** (300 MHz, $CDCl_3$) δ7.95-7.45 (m, 17H), 4.68 (t, *J* = 12.3Hz, 1H), 4.38 (s, 2H), 2.37-2.27 (m, 3H), 2.07-2.04 (m, 2H), 1.76-1.72 (m, 2H), 1.04-0.95 (m, 2H). **HRMS (ESI)** $C_{33}H_{30}NO_2^+$ [M-BF4] calcd: 472.2272, found: 472.2263.

Synthesis of polypeptide pyridinium[10,11]**.**

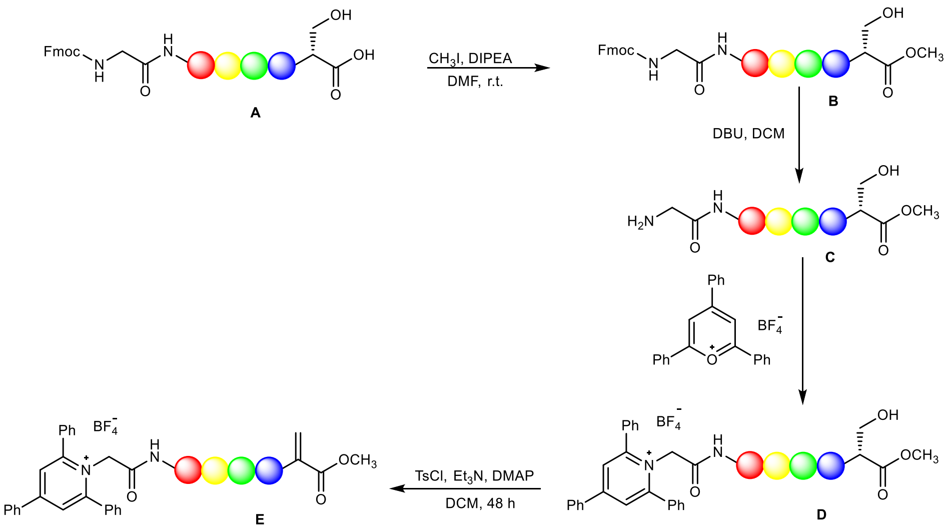

Polypeptide **A** (0.2 mmol) was prepared via standard solid phases peptide synthesis procedure. To a 10 mL round bottom flask, **A** (0.2 mmol) was dissolved by DMF (1 mL). CH_3I (0.5 mmol, 2.5 equiv) and DIPEA (0.4 mmol, 2 equiv) were added slowly. Then, the reaction mixture was stirred at room temperature. After 12 h, the reaction mixture was slowly added to the stirred water. The undissolved residue was filtrated and dried under a vacuum to give product **B** as a light-yellow solid, which was used for the next step without further purification.

DBU (0.6 mmol, 3.0 equiv) was added into the solution of **B** in CH_2Cl_2 at room temperature. The reaction was monitored by TLC. When the reaction was completed, the mixture was concentrated under vacuum to give the crude product of polypeptide **C**, which was used for the next step without further purification.

Polypeptide **C** (0.2 mmol), 2,4,6-triphenylpyrylium tetrafluoroborate (0.2 mmol, 1.0 equiv), and activated 4Å MS (0.5 g/mmol) were added to a round-bottomed flask. The flask was capped with a septum, and air was withdrawn and backfilled with Ar (three times). Then, CH_2Cl_2 (0.5 M) and Et_3N (0.4 mmol, 2.0 equiv) were added by syringes. The reaction mixture was stirred for 30 min at room temperature. Then, acetic acid (0.4 mmol, 2.0 equiv) was added and the mixture was stirred for a next 5 h at room temperature. The reaction mixture was filtered through a short celite pad with the flask and celite rinsed by CH_2Cl_2. The liquid phase was concentrated and the crude product was purified by silica gel chromatography with CH_2Cl_2 and CH_3OH as eluents to give the product **D** (yield ~ 40%).

TsCl (0.16 mmol, 2 equiv) and DMAP (0.016 mmol, 0.2 equiv) were added into the solution of peptide pyridinium salt (**D**, 0.08 mmol, 1.0 equiv). Then, Et_3N (0.16 mmol, 2 equiv) was added slowly into the reaction mixture, and it was stirred at room temperature for 48 h. After reaction was completed, the reaction mixture was concentrated and purified by silica gel chromatography with CH_2Cl_2 and CH_3OH as eluents to give the product **E** (yield ~ 40%).

Characterization of polypeptide pyridinium salts.

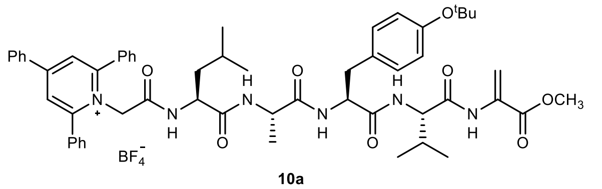

10a

1-((7*S*,10*S*,13*S*,16*S*)-10-(4-(tert-butoxy)benzyl)-16-isobutyl-7-isopropyl-13-methyl-4-methylene-3,6,9,12,15,18-hexaoxo-2-oxa-5,8,11,14,17-pentaazanonadecan-19-yl)-2,4,6-triphenylpyridin-1-ium tetrafluoroborate.

Prepared from the procedure outlined above. 0.2 mmol polypeptide was used as the starting material. Brown solid (27 mg, yield: 13%), **^{1}H NMR** (300 MHz, CD_3OD) δ 8.37 (s, 2H), 8.08-8.05 (m, 2H), 7.63-7.53 (m, 13H), 6.84 (d, *J* = 8.4 Hz, 2H), 6.62 (d, *J* = 8.4 Hz, 2H), 6.19 (s, 1H), 5.75 (s,1H), 4.57-4.53 (m, 1H), 4.36-4.23 (m, 2H), 4.10-4.01 (m, 1H), 3.77 (s, 3H), 2.92-2.87 (m, 2H), 2.56-2.51 (m, 1H), 2.13-2.07 (m, 1H), 1.41-1.21(m, 6H), 1.20 (s, 9H), 0.95-0.85 (m, 13H). **HRMS (ESI)** $C_{56}H_{67}N_6O_8^+$ [M-BF_4] calcd: 951.5015, found: 951.5025.

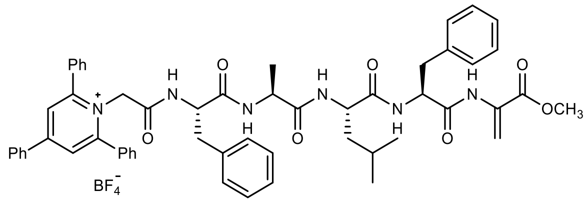

10b

1-((7*S*,10*S*,13*S*,16*S*)-7,16-dibenzyl-10-isobutyl-13-methyl-4-methylene-3,6,9,12,15,18-hexaoxo-2-oxa-5,8,11,14,17-pentaazanonadecan-19-yl)-2,4,6-triphenylpyridin-1-ium tetrafluoroborate.

Prepared from the procedure outlined above. 0.5 mmol polypeptide was used as the starting material. Brown oil (81 mg, yield: 16%), **^{1}H NMR** (300 MHz, CD_3OD) δ 8.23 (s, 2H), 7.95 (d, *J* = 6.9 Hz, 2H), 7.70-7.63 (m, 2H), 7.52-7.46 (m, 11H), 7.14-7.11 (m, 8H), 6.93-6.92 (m, 2H), 6.22 (s, 1H), 5.75 (s,1H), 4.63-4.59 (m, 1H), 4.42-4.37 (m, 1H), 4.31-4.17 (m, 2H), 3.68 (s, 3H), 3.09-3.03 (m, 2H), 2.91-2.76 (m, 3H), 2.58-2.50 (m, 1H), 1.43-1.35 (m,3H), 0.82-0.76 (m, 9H). **HRMS (ESI)** $C_{56}H_{59}N_6O_7$ [M-BF_4]$^+$ calcd: 927.4440, found: 927.4440.

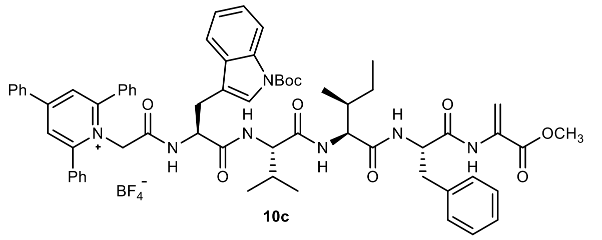

10c

1-((7*S*,10*S*,13*S*,16*S*)-7-benzyl-16-((1-(tert-butoxycarbonyl)-1H-indol-3-yl)methyl)-10-((S)-sec-butyl)-13-isopropyl-4-methylene-3,6,9,12,15,18-hexaoxo-2-oxa-5,8,11,14,17-pentaazanonadecan-19-yl)-2,4,6-triphenylpyridin-1-ium tetrafluoroborate.

Prepared from the procedure outlined above. 0.2 mmol polypeptide was used as the starting material. Brown solid (35 mg, yield: 15%), **^{1}H NMR** (300 MHz, CD_3OD) δ 8.35 (s, 2H), 8.10-8.04 (m, 2H), 7.65-7.60 (m, 13H),7.28-7.23 (m, 10H), 6.33 (s, 1H), 5.85 (s,1H), 4.70-4.65 (m, 1H), 4.29-4.17 (m, 2H), 4.10-4.07 (m,1H), 3.77 (s, 3H), 3.02-2.92 (m, 3H), 2.84-2.75 (m, 1H), 2.04-2.02 (m, 2H), 1.34 (s, 9H), 0.95-0.92 (m,7H), 0.79-0.67 (m, 9H). **HRMS (ESI)** $C_{65}H_{72}N_7O_9$ [M-BF_4]$^+$ calcd:

1094.5387, found: 1094.5368.

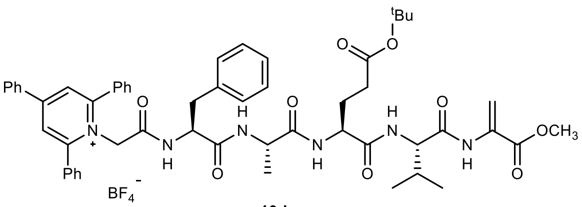

10d

1-((7*S*,10*S*,13*S*,16*S*)-16-benzyl-10-(3-(tert-butoxy)-3-oxopropyl)-7-isopropyl-13-methyl-4-methylene-3,6,9,12,15,18-hexaoxo-2-oxa-5,8,11,14,17-pentaazanonadecan-19-yl)-2,4,6-triphenylpyridin-1-ium tetrafluoroborate.

Prepared from the procedure outlined above. 0.2 mmol polypeptide was used as the starting material. Brown solid (35 mg, yield: 17%), ^{1}**H NMR** (300 MHz, CD_3OD) δ 8.25 (s, 2H), 7.99-7.95 (m, 2H), 7.55-7.58 (m, 13H),7.13-7.11 (m, 3H), 6.95-6.92 (m, 2H), 6.20 (s, 1H), 5.77 (s,1H), 4.41-4.28 (m, 2H), 4.26-4.19 (m, 2H), 3.71 (s, 3H), 2.91-2.85 (m, 1H), 2.60-2.49 (m, 1H), 2.23-2.18 (m, 2H), 2.14-1.93 (m, 3H), 1.84-1.74 (m, 1H), 1.58-1.50 (m,1H), 1.31 (s, 9H), 0.86 (d, J = 5.4 Hz, 6H), 0.82-0.78 (m, 3H). **HRMS (ESI)** $C_{55}H_{63}N_6O_9$ $[M\text{-}BF_4]^+$ calcd: 951.4657, found: 951.4658.

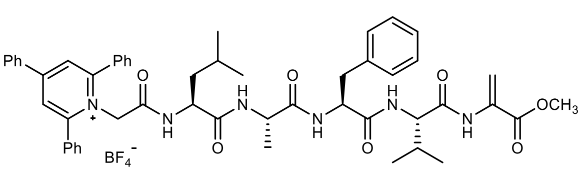

10e

1-((7*S*,10*S*,13*S*,16*S*)-10-benzyl-16-isobutyl-7-isopropyl-13-methyl-4-methylene-3,6,9,12,15,18-hexaoxo-2-oxa-5,8,11,14,17-pentaazanonadecan-19-yl)-2,4,6-triphenylpyridin-1-ium tetrafluoroborate.

Prepared from the procedure outlined above. 0.2 mmol polypeptide was used as the starting material. Black oil (40.6 mg, yield: 21%), ^{1}**H NMR** (300 MHz, CD_3OD) δ 8.35 (s, 2H), 8.09-8.05 (m, 2H), 7.68-7.58 (m, 13H), 7.24-7.15 (m, 5H), 6.29 (s, 1H), 5.88 (s, 1H), 4.70-4.58 (m, 1H), 4.26-4.17 (m, 3H), 3.80 (s, 3H), 3.14-3.08 (m, 2H), 3.00-2.89 (m, 2H), 2.15-2.04 (m, 1H), 1.79-1.58 (m, 3H), 1.00-0.83 (m, 15H). **HRMS (ESI)** $C_{52}H_{59}N_6O_7$ $[M\text{-}BF_4]^+$ calcd: 879.4440, found: 879.4431.

3. General procedures

3.1 Optimization of Condition A.

Supplementary Table 1. Light Sources Screening.

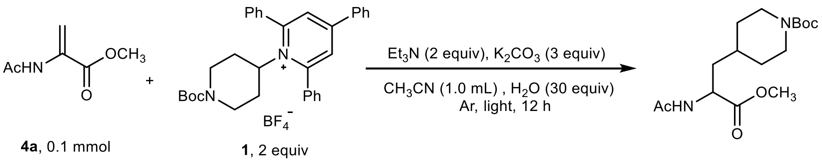

4a, 0.1 mmol **1**, 2 equiv

Entry	Light source	yield(%)[a]
1	380 nm	84%
2	395-400 nm	84%
3	410-420 nm	90%
4	420-430 nm	85%
5	blue LED	n.d
6	white LED	n.d
7	no light	n.d

[a]Yield was determined by ^{1}H NMR using 4-bromobenzaldehyde as an internal standard.

Supplementary Table 2. Screening of Alkyl Amines.

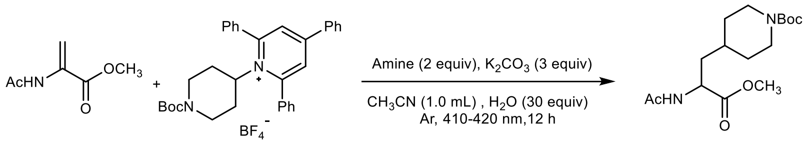

4a, 0.1 mmol **1**, 2 equiv

Entry	Amine	Yield(%)[a]
1	Et_3N	90%
2	DIPEA	67%
3	DABCO	n.d
4	triethanolamine	69%
5	DMAP	14%
6	pyridine	n.d

[a]Yield was determined by ^{1}H NMR using 4-bromobenzaldehyde as an internal standard.

Supplementary Table 3. Basic Ionic Compounds Screening.

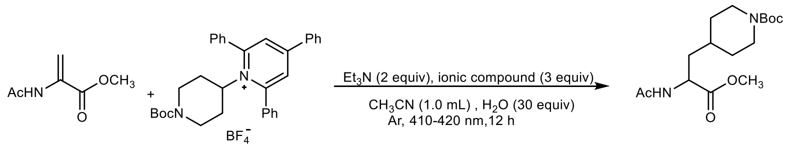

4a, 0.1 mmol **1**, 2 equiv

Entry	Ionic Compound	Yield(%)[a]	Entry	Ionic Compound	Yield(%)[a]
1	K_2CO_3	90%	6	$NaOCH_3$	14%
2	KOAc	60%	7	^tBuONa	13%
3	K_3PO_4	60%	8	^tBuOLi	n.d
4	$KOCH_3$	46%	9	Na_2CO_3	47%
5	CsF	57%	10	KBF_4 or $NaBF_4$	0%

[a]Yield was determined by ^1H NMR using 4-bromobenzaldehyde as an internal standard.

Supplementary Table 4. Solvents Screening.

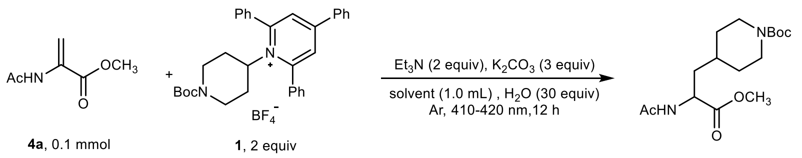

4a, 0.1 mmol **1**, 2 equiv

Entry	Solvent	Yield(%)[a]
1	CH_3CN	90%
2	acetone	84%
3	THF	49%
4	EtOAc	30%
5	CH_2Cl_2	66%
6	toluene	trace
7	DMF	38%

[a]Yield was determined by ^1H NMR using 4-bromobenzaldehyde as an internal standard.

Supplementary Table 5. Screening the Concentration of Reaction Mixtures.

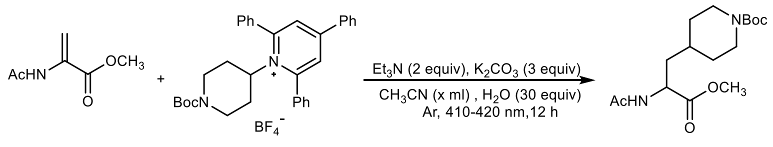

4a, 0.1 mmol **1**, 2 equiv

Entry	x (mL)	Yield(%)[a]
1	0.5 mL	76%
2	1.0 mL	90%
3	1.5 mL	92%
4	2.0 mL	90%
5	2.5 mL	90%

[a]Yield was determined by ^1H NMR using 4-bromobenzaldehyde as an internal standard.

Supplementary Table 6. Screening the Loading of H_2O

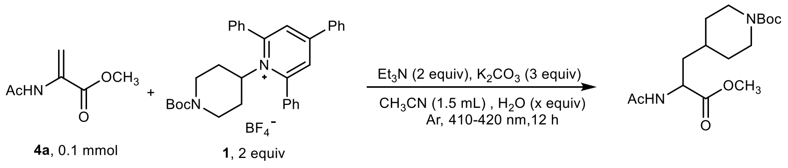

Entry	x(equiv)	Yield(%)[a]
1	10 equiv	62%
2	20 equiv	78%
3	30 equiv	92%
4	40 equiv	80%
5	50 equiv	84%

[a]Yield was determined by ^1H NMR using 4-bromobenzaldehyde as an internal standard.

Supplementary Table 7. Screening the Loading of K_2CO_3.

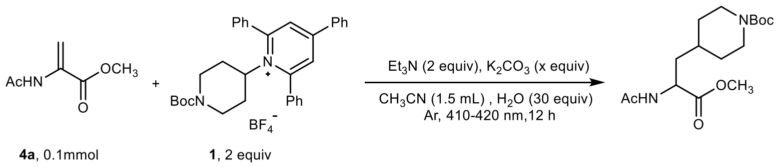

Entry	x (equiv)	Yield(%)[a]
1	0.25 equiv	71%
2	0.5 equiv	85%
3	0.75 equiv	81%
4	1.0 equiv	95%
5	1.5 equiv	93%
6	2.0 equiv	94%
7	2.5 equiv	92%
8	3.0 equiv	92%
9	3.5 equiv	94%
10	4.0 equiv	95%

[a]Yield was determined by ^1H NMR using 4-bromobenzaldehyde as an internal standard.

Supplementary Table 8. Screening the Loading of 1.

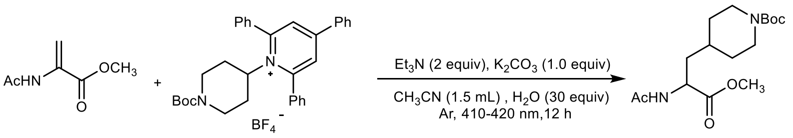

4a, 0.1 mmol **1**, x equiv

Entry	x (equiv)	Yield(%)[a]
1	1.0 equiv	48%
2	1.5 equiv	82%
3	2.0 equiv	95%
4	2.5 equiv	97% (94%)
5	3.0 equiv	97%

[a]Yield was determined by ^{1}H NMR using 4-bromobenzaldehyde as an internal standard. The value within parentheses refers to isolated yield.

Supplementary Table 9. Screening the Loading of Et3N.

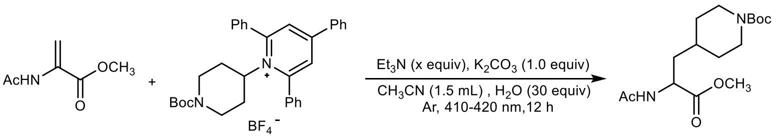

4a, 0.1 mmol **1**, 2.5 equiv

Entry	x (equiv)	Yield(%)[a]
1	0.5 equiv	26%
2	1.0 equiv	73%
3	1.5 equiv	92%
4	2.0 equiv	97% (94%)
5	2.5 equiv	92%
6	3.0 equiv	84%

[a]Yield was determined by ^{1}H NMR using 4-bromobenzaldehyde as an internal standard. The value within parentheses refers to isolated yield.

Supplementary Table 10. Influences of Reaction Time.

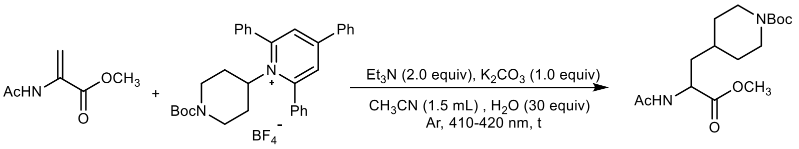

4a, 0.1 mmol **1**, 2.5 equiv

Entry	t (h)	Yield(%)[a]
1	3 h	45%
2	6 h	69%
3	9 h	92%
4	12 h	97% (94%)
5	15 h	89%

[a]Yield was determined by 1H NMR using 4-bromobenzaldehyde as an internal standard. The value within parentheses refers to isolated yield.

3.2 Optimization of Condition B.

Supplementary Table 11. Light Sources Screening.

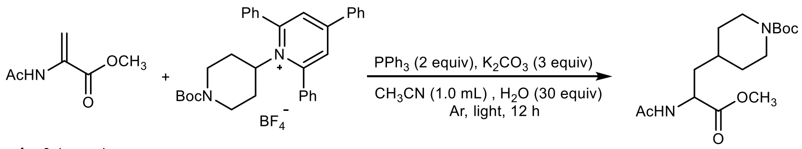

4a, 0.1 mmol **1**, 2 equiv

Entry	Light source	yield(%)[a]
1	380 nnm	30%
2	395-400 nm	34%
3	410-420 nm	37%
4	420-430 nm	44%
5	blue light	n.d
6	white light	n.d

[a]Yield was determined by ^1H NMR using 4-bromobenzaldehyde as an internal standard.

Supplementary Table 12. Screening of Phosphine.

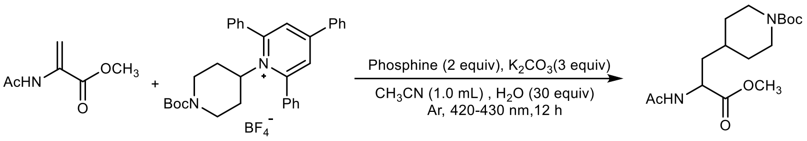

4a, 0.1 mmol **1**, 2 equiv

Entry	Phosphine	Yield(%)[a]
1	PPh_3	44%
2	$(CH_3)_2PhPh_2$	22%
3	$(4\text{-F-Ph})_3P$	28%
4	$BnPh_2P$	30%

[a]Yield was determined by ^1H NMR using 4-bromobenzaldehyde as an internal standard.

Supplementary Table 13. Basic Ionic Compounds Screening.

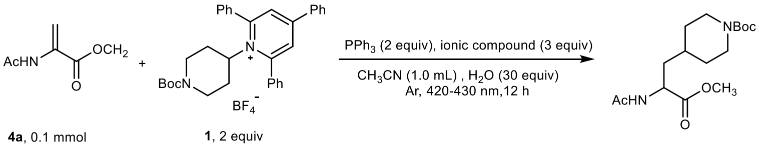

4a, 0.1 mmol **1**, 2 equiv

Entry	Ionic Compound	Yield(%)[a]	Entry	Ionic Compound	Yield(%)[a]
1	K_2CO_3	44%	10	Cs_2CO_3	12%
2	$KHCO_3$	33%	11	^tBuONa	n.d
3	KOAc	33%	12	$NaHCO_3$	14%
4	KO^tBu	11%	13	Na_2CO_3	10%
5	K_3PO_4	38%	14	NaOAc	20%
6	K_2HPO_4	24%	15	NaOH	23%
7	$KOCH_3$	54%	16	^tBuOLi	10%
8	KF	n.d	17	$NaOCH_3$	27%
9	CsF	17%	18	KBF_4 or $NaBF_4$	0%

[a]Yield was determined by ^1H NMR using 4-bromobenzaldehyde as an internal standard.

Supplementary Table 14. Solvents Screening.

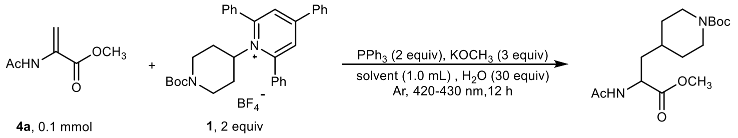

Entry	Solvent	Yield(%)[a]	Entry	Solvent	Yield(%)[a]
1	CH_3CN	54%	11	^{t}BuOH	62%
2	acetone	73%	12	1,4-dioxane	69%
3	THF	60%	13	$CHCl_3$	8%
4	EtOAc	65%	14	DMAc	54%
5	NMP	n.d	15	toluene	13%
6	DME	n.d	16	DMSO	15%
7	DMF	63%	17	DCE	42%
8	CH_2Cl_2	62%	18	1,3-dioxolane	62%
9	EtOH	n.d	19	CCl_4	n.d
10	HFIP	n.d	20	CF_3CH_2OH	n.d

[a]Yield was determined by ^{1}H NMR using 4-bromobenzaldehyde as an internal standard.

Supplementary Table 15. Screening the Concentration of Reaction Mixtures.

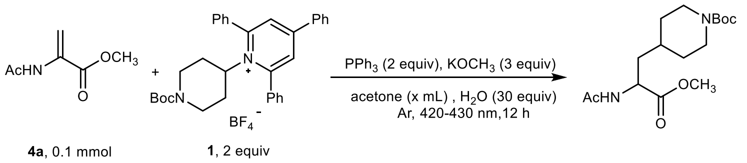

Entry	x (mL)	Yield(%)[a]
1	0.5 mL	20%
2	1.0 mL	73%
3	1.5 mL	76%
4	2.0 mL	70%

[a]Yield was determined by ^{1}H NMR using 4-bromobenzaldehyde as an internal standard.

Supplementary Table 16. Screening the Loading of H_2O.

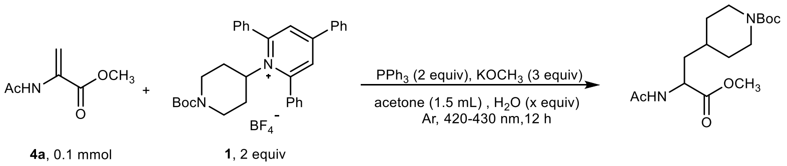

Entry	x(equiv)	Yield(%)[a]
1	5 equiv	26%
2	10 equiv	43%
3	15 equiv	62%
4	20 equiv	75%
5	25 equiv	79%
6	30 equiv	76%
7	40 equiv	65%
8	50 equiv	61%

[a]Yield was determined by ^{1}H NMR using 4-bromobenzaldehyde as an internal standard.

Supplementary Table 17. Screening the Loading of $KOCH_3$.

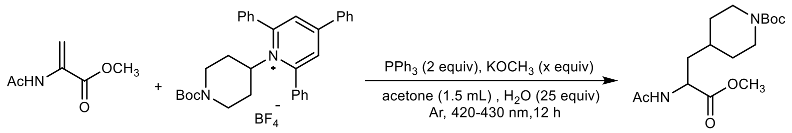

4a, 0.1 mmol **1**, 2 equiv

Entry	x (equiv)	Yield(%)[a]
1	1.5 equiv	64%
2	2.0 equiv	70%
3	2.5 equiv	70%
4	3.0 equiv	79%
5	3.5 equiv	70%

[a]Yield was determined by ^{1}H NMR using 4-bromobenzaldehyde as an internal standard.

Supplementary Table 18. Screening the Loading of PPh_3.

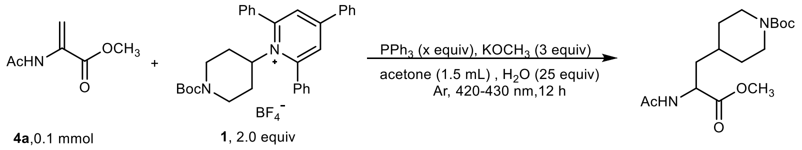

4a,0.1 mmol **1**, 2.0 equiv

Entry	x (equiv)	Yield(%)[a]
1	1.0 equiv	60%
2	2.0 equiv	79%
3	2.4 equiv	88% (84%)
4	3.0 equiv	77%
5	4.0 equiv	65%
6	5.0 equiv	67%

[a]Yield was determined by ^{1}H NMR using 4-bromobenzaldehyde as an internal standard. The value within parentheses refers to isolated yield.

Supplementary Table 19. Screening the Loading of 1.

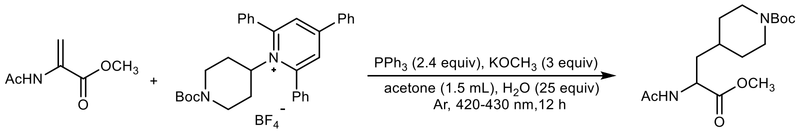

4a, 0.1 mmol **1**, x equiv

Entry	x (equiv)	Yield(%)[a]
1	1.2 equiv	52%
2	1.5 equiv	65%
3	2.0 equiv	88% (84%)
4	2.5 equiv	79%
5	3.0 equiv	75%

[a]Yield was determined by ^{1}H NMR using 4-bromobenzaldehyde as an internal standard. The value within parentheses refers to isolated yield.

Supplementary Table 20. Influences of Reaction Time.

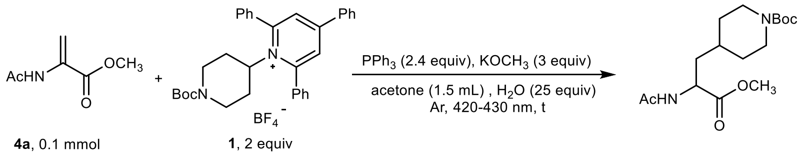

Entry	t (h)	Yield(%)[a]
1	6 h	51%
2	9 h	77%
3	12 h	88% (84%)
4	15 h	76%
5	18 h	79%

[a]Yield was determined by ^1H NMR using 4-bromobenzaldehyde as an internal standard. The value within parentheses refers to isolated yield.

3.3 General procedure for deaminative hydroalkylation of alkenes

General procedure of Condition A.

To an oven-dried 10 mL quartz test tube with a stirring bar was added alkene (0.2 mmol), alkyl pyridinium salt (0.5 mmol, 2.5 equiv), Et_3N (0.4 mmol, 2 equiv), and K_2CO_3 (0.2 mmol, 1 equiv). Then, air was withdrawn and backfilled with Ar (three times). CH_3CN (3 mL) and H_2O (6 mmol, 30 equiv) were added. The mixture was transferred to a violet LED photoreactor (24-W, 410-420 nm), where it was irradiated for 12 h. Then, the reaction was quenched with water (5 mL), extracted with ethyl acetate, washed with brine, dried over anhydrous sodium sulfate, concentrated in vacuo, and purified by column chromatography (hexane/acetone) to afford the product.

General procedure of Condition B.

To an oven-dried 10 mL quartz test tube with a stirring bar was added alkene (0.2 mmol), alkyl pyridinium salt (0.4 mmol, 2.0 equiv), PPh_3 (0.48 mmol, 2.4 equiv), and $KOCH_3$ (0.6 mmol, 3.0 equiv). Then, air was withdrawn and backfilled with Ar (three times). Acetone (3 mL) and H_2O (5 mmol, 25 equiv) were added. The mixture was transferred to a violet LED photoreactor (24-W, 420-430 nm), where it was irradiated for 12 h. Then, the reaction was quenched with water (5 mL), extracted with ethyl acetate, washed with brine, dried over anhydrous sodium sulfate, concentrated in vacuo, and purified by column chromatography (hexane/acetone) to afford the product.

General procedure for macrocyclization of peptides.

To an oven-dried 10 mL quartz test tube with a stirring bar was added polypeptide pyridinium salt (0.02 mmol, 1.0 equiv), Et_3N (0.04 mmol, 2 equiv), K_2CO_3 (0.02 mmol, 1 equiv). Then, air was withdrawn and backfilled with Ar (three times). CH_2Cl_2 (1 mL) and H_2O (0.5 mmol, 25 equiv) was added. The mixture was transferred to a violet LED photoreactor (24-W, 410-420 nm), where it was irradiated for 12 h. Then, the reaction was quenched with water (5 mL), extracted with ethyl acetate, washed with brine, dried over anhydrous sodium sulfate, concentrated in vacuo, and isolated by HPLC purification.

Picture of the reaction photo set-up (410-420 nm)

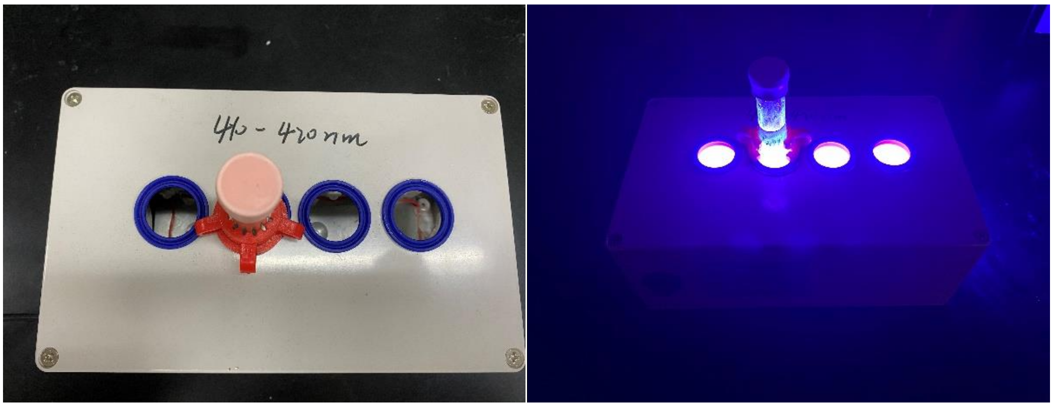

We also measured the wavelength of the LED light by ourselves (recorded on an AVANTES® AvaSpec-ULS2048 spectrometer instrument). The result was shown as follow:

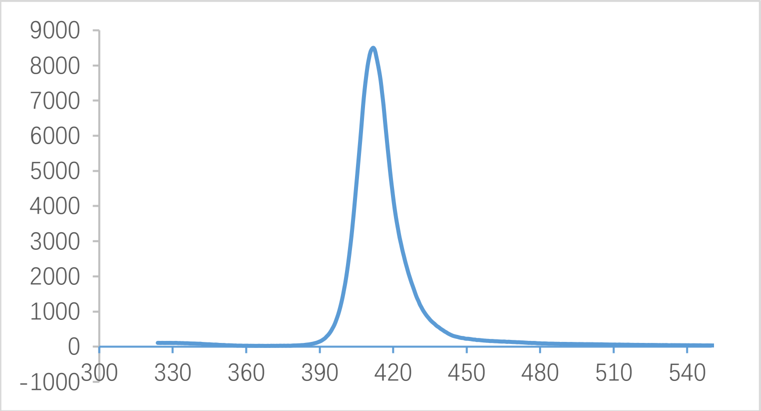

Picture of the reaction photo set-up (420-430 nm)

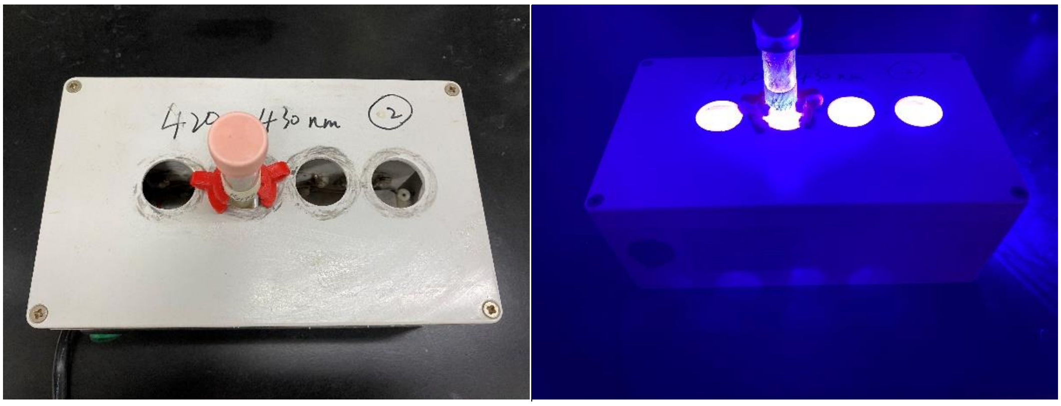

We also measured the wavelength of the LED light by ourselves (recorded on an AVANTES® AvaSpec-ULS2048 spectrometer instrument). The result was shown as follow:

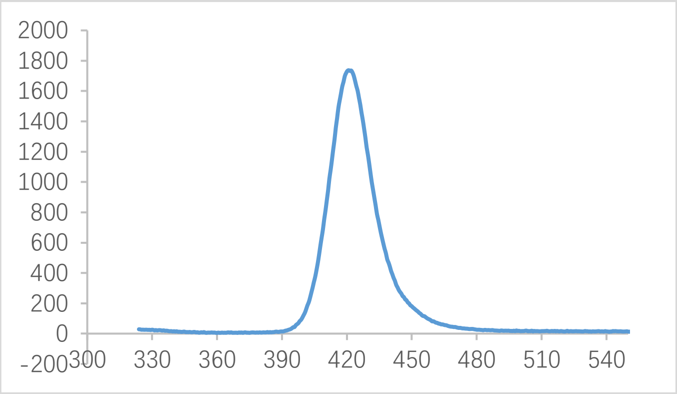

4. Characterization of products.

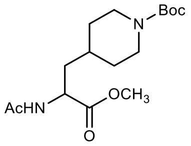

5a

tert-butyl 4-(2-acetamido-3-methoxy-3-oxopropyl)piperidine-1-carboxylate: 61.7 mg, yield: 94% under Condition A; 55.1 mg, yield: 84% under Condition B, colorless oil. **^{1}H NMR** (300 MHz, $CDCl_3$) δ 6.02 (d, *J* = 9.0 Hz, 1H), 4.73-4.66 (m, 1H), 4.07 (br, 2H), 3.75 (s, 3H), 2.66 (t, *J* = 12.0 Hz, 2H), 2.04(s, 3H), 1.82-1.52 (m, 5H), 1.45 (s, 9H), 1.21-1.04 (m, 2H). **^{13}C NMR** (75 Hz, $CDCl_3$) δ 173.46, 169.90, 154.79, 79.37, 52.47, 49.74, 43.60, 39.64, 32.59, 32.22, 31.46, 28.44, 23.23. **HRMS (ESI)** $C_{16}H_{28}N_2NaO_5$ $[M+Na]^+$ calcd: 351.1896, found: 351.1892.

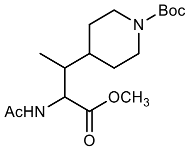

5b

tert-butyl 4-(3-acetamido-4-methoxy-4-oxobutan-2-yl)piperidine-1-carboxylate: 54.7 mg, yield: 80%, d.r. = 4:1 under Condition A; 52.0 mg, yield: 76%, d.r. = 4:1 under Condition B. Colorless oil. **^{1}H NMR**(300 MHz, $CDCl_3$) δ 6.18 (d, *J* = 9.0 Hz, 0.76H), 5.99 (d, *J* = 9.0 Hz, 0.2H), 4.89 (dd, *J* = 3.0 Hz, *J* = 6.0 Hz, 0.23H), 4.69 (t, *J* = 7.5 Hz, 0.79H), 4.12 (br, 2H), 3.75-3.74 (m, 3H), 2.66-2.62 (m, 2H), 2.06-2.03 (m, 3H), 1.75-1.65 (m, 3H), 1.53-1.40 (m, 10H), 1.36-1.31 (m, 1H), 1.18-1.05 (m, 1H), 0.84 (d, *J* = 6.0Hz, 3H). **^{13}C NMR** (75 MHz, $CDCl_3$) δ 173.22, 172.57, 170.18, 169.51, 154.73, 79.31, 54.20, 53.42, 52.38, 52.11, 41.46, 40.56, 37.96, 37.48, 30.28, 29.11, 28.43, 27.99, 23.24, 12.26, 11.85. **HRMS (ESI)** $C_{17}H_{30}N_2NaO_5$ $[M+Na]^+$ calcd: 365.2052, found: 365.2052.

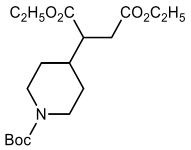

5c

diethyl 2-(1-(tert-butoxycarbonyl)piperidin-4-yl)succinate: 60.7 mg, yield: 85% under Condition A; 55.7 mg, yield: 78% under Condition B. Yellow oil. **^{1}H NMR** (300 MHz, $CDCl_3$) δ

4.20-4.09 (m, 5H), 2.79-2.61 (m, 4H), 2.49-2.39 (m, 1H), 1.78-1.55 (m, 4H), 1.45 (s, 9H), 1.29-1.22 (m, 8H). ^{13}**C NMR** (75 MHz, $CDCl_3$) δ 173.88, 172.08, 154.69, 79.49, 60.71, 60.65, 46.31, 43.75, 38.30, 33.47, 29.54, 29.25, 28.43, 14.24, 14.14. **HRMS (ESI)** $C_{18}H_{31}NNaO_6$ $[M+Na]^+$ calcd: 380.2049, found: 380.2063.

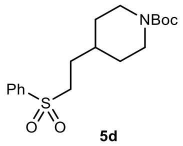

5d

tert-butyl 4-(2-(phenylsulfonyl)ethyl)piperidine-1-carboxylate: 67.0 mg, yield: 95% under Condition A; 60.6 mg, yield: 86% under Condition B. Colorless oil. ^{1}**H NMR** (300 MHz, $CDCl_3$) δ 7.91 (d, J = 9.0 Hz, 2H), 7.70-7.65 (m, 1H), 7.61-7.56 (m, 2H), 4.06 (br, 2H), 3.14-3.08 (m, 2H), 2.63 (t, J = 12.0 Hz, 2H), 1.71-1.57 (m, 4H), 1.52-1.44 (m, 10H), 1.13-0.99 (m, 2H).^{13}**C NMR** (75 MHz, $CDCl_3$) δ 154.69, 139.02, 133.77, 129.34, 127.98, 79.41, 53.86, 43.60, 34.89, 31.60, 28.89, 28.41. **HRMS (ESI)** $C_{18}H_{27}NNaO_4S$ $[M+Na]^+$ calcd: 376.1558, found: 376.1560.

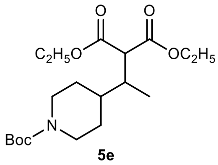

5e

diethyl 2-(1-(1-(tert-butoxycarbonyl)piperidin-4-yl)ethyl)malonate: 63.1 mg, yield: 85% under Condition A; 58.5 mg, yield: 79% under Condition B. Colorless oil. ^{1}**H NMR** (300 MHz, $CDCl_3$) δ 4.24-4.14 (m, 6H), 3.39 (d, J = 8.7 Hz, 1H), 2.66-2.54 (m, 2H), 2.27-2.16 (m, 1H), 1.63-1.48 (m, 3H), 1.45 (s, 9H), 1.37-1.33(m, 1H), 1.27 (t, J = 14.4 Hz, 6H), 1.22-1.08 (m, 1H), 0.93 (d, J = 6.9 Hz, 3H).^{13}**C NMR** (75 MHz, $CDCl_3$) δ 169.04, 168.68, 154.74, 79.32, 61.31, 61.20, 55.34, 43.73, 38.74, 37.73, 30.30, 28.43, 26.96, 14.10, 12.98. **HRMS (ESI)** $C_{19}H_{33}NNaO_6$ $[M+Na]^+$ calcd: 394.2206, found: 394.2205.

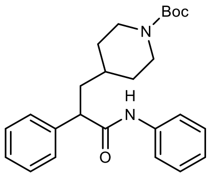

5f

tert-butyl 4-(3-oxo-2-phenyl-3-(phenylamino)propyl)piperidine-1-carboxylate: 77.5 mg, yield: 95% under Condition A; 75.1 mg, yield: 92% under Condition B. White solid. M. p. 174-176 °C. ^{1}**H NMR** (300 MHz, $CDCl_3$) δ 8.51 (s, 1H), 7.47 (d, J = 6.0 Hz, 2H), 7.37 (d, J = 9.0 Hz, 2H), 7.31-7.18 (m, 5H), 7.04-6.99 (m, 1H), 4.01 (br, 2H), 3.73 (t, J = 7.5 Hz, 1H), 2.55-2.52 (m, 2H), 2.14-2.03 (m, 1H), 1.80-1.60 (m, 3H), 1.44 (s, 9H), 1.38-1.23 (m, 1H), 1.12-1.01 (m, 2H). ^{13}**C NMR** (75 MHz, $CDCl_3$) δ 172.09, 154.98, 139.88, 138.30, 128.88, 128.82, 127.90, 127.36, 124.16, 120.04, 79.52, 50.53, 43.57, 40.11, 33.53, 32.25, 28.52. **HRMS (ESI)** $C_{25}H_{32}N_2NaO_3$ $[M+Na]^+$ calcd: 431.2311, found: 431.2306.

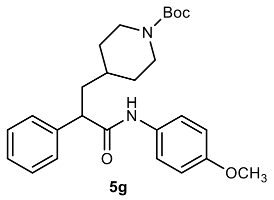

5g

tert-butyl 4-(3-((4-methoxyphenyl)amino)-3-oxo-2-phenylpropyl)piperidine-1-carboxylate: 78.9 mg, yield: 90% under Condition A; 77.2 mg, yield: 88% under Condition B. Brown solid. M. p. 160-162 °C. **^{1}H NMR** (300 MHz, $CDCl_3$) δ 8.02 (s, 1H), 7.35-7.25 (m, 7H), 6.76 (d, J = 9.0 Hz, 2H), 4.01 (br, 2H), 3.72 (s, 3H), 3.67 (t, J = 7.5 Hz, 1H), 2.58 (br, 2H), 2.21-2.12 (m, 1H), 1.67-1.63 (m, 3H), 1.44 (s, 9H), 1.37-1.35 (m, 1H), 1.12-1.08 (m, 2H). **^{13}C NMR** (75 MHz, $CDCl_3$) δ 171.67, 156.26, 154.92, 139.91, 131.27, 128.91, 127.90, 127.37, 121.75, 113.94, 79.43, 55.44, 50.48, 43.91, 40.01, 33.58, 32.13, 28.48. **HRMS (ESI)** $C_{26}H_{34}N_2NaO_4$ $[M+Na]^+$ calcd: 461.2416, found: 461.2418.

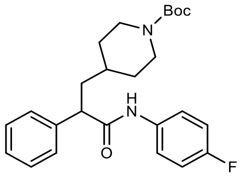

5h

tert-butyl4-(3-((4-fluorophenyl)amino)-3-oxo-2-phenylpropyl)piperidine-1-carboxylate: 76.7 mg, yield: 90% under Condition A; 71.5 mg, yield: 84% under Condition B. white solid. M. p. 190-192 °C. **^{1}H NMR (300 MHz, $CDCl_3$)** δ 8.04 (s, 1H), 7.42-7.27 (m, 7H), 6.92 (t, J = 9.0 Hz, 2H), 4.03 (br, 2H), 3.68 (t, J = 7.5 Hz, 1H), 2.60-2.56 (m, 2H), 2.16-2.08 (m, 1H), 1.70-1.65(m, 3H), 1.44 (s, 9H), 1.39-1.33 (m, 1H), 1.17-1.06 (m, 2H). **^{19}F NMR** (282 MHz, $CDCl_3$) δ -118.09. **^{13}C NMR** (75 MHz, $CDCl_3$) δ 171.80,160.84, 157.62, 154.96, 139.55, 134.13, 134.09, 129.02, 127.89, 127.54, 121.75, 121.64, 115.56, 115.27, 79.55, 50.54, 43.86, 39.91, 33.50, 32.12, 28.48. **HRMS (ESI)** $C_{25}H_{31}FN_2NaO_3$ $[M+Na]^+$ calcd: 449.2216, found: 449.2212.

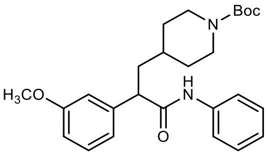

5i

tert-butyl 4-(2-(3-methoxyphenyl)-3-oxo-3-(phenylamino)propyl)piperidine-1-carboxylate: 76.3 mg, yield: 87% under Condition A; 72.8 mg, yield: 83% under Condition B. Colorless oil. **^{1}H NMR** (300 MHz, $CDCl_3$) δ 7.97 (s, 1H), 7.46 (d, J = 7.8Hz, 2H), 7.26-7.21 (m,3H), 7.06-7.01 (m, 1H), 6.95-6.93 (m, 2H), 6.82-6.79 (m, 1H), 4.01 (br, 2H), 3.77 (s, 3H), 3.66 (t, J = 7.5 Hz, 1H), 2.58 (br, 2H), 2.26-2.04 (m, 1H), 1.74-1.48 (m, 3H), 1.44-1.21 (m, 10H), 1.19-0.98 (m, 2H). **^{13}C NMR** (75MHz, $CDCl_3$) δ 171.65, 160.01, 154.92, 141.28, 138.12, 129.98, 128.85, 124.18, 120.26, 119.84, 113.77, 112.57, 79.44, 55.21, 50.72, 43.62, 39.87, 33.54, 32.18, 28.49. **HRMS (ESI)** $C_{26}H_{34}N_2NaO_4$ $[M+Na]^+$ calcd: 461.2411, found: 461.2409.

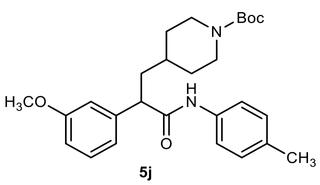

5j

tert-butyl 4-(2-(3-methoxyphenyl)-3-oxo-3-(p-tolylamino)propyl)piperidine-1-carboxylate: 76.9 mg, yield: 85% under Condition A; 76.0 mg, yield: 84% under Condition B. Colorless oil. **^{1}H NMR** (300 MHz, $CDCl_3$) δ 7.42 (s, 1H), 7.33-7.24 (m, 3H), 7.06 (d, J = 9.0 Hz, 2H), 6.94-6.90 (m, 2H), 6.84-6.81 (m, 1H), 4.03 (br, 2H), 3.80 (s, 3H), 3.61 (t, *J* = 7.5 Hz, 1H), 2.59 (br, 2H), 2.27 (s, 3H), 2.23-2.06 (m, 1H), 1.76-1.62 (m, 3H), 1.44(s, 9H), 1.40-1.34 (m, 1H), 1.19-1.05 (m, 2H). **^{13}C NMR** (75 MHz, $CDCl_3$), 171.30, 160.07, 154.88, 141.23, 135.36, 133.87, 130.09, 129.36, 120.25, 119.82, 113.75, 112.67, 79.35, 55.25, 50.86, 44.00, 39.74, 33.56, 32.33, 31.97, 28.47, 20.84. **HRMS (ESI)** $C_{27}H_{36}N_2NaO_4$ $[M+Na]^+$ calcd: 475.2573, found: 475.2565.

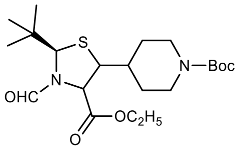

5k

ethyl (2R)-5-(1-(tert-butoxycarbonyl)piperidin-4-yl)-2-(tert-butyl)-3-formylthiazolidine-4-carboxylate: 68.5 mg, yield: 80%, r.r. = 5.7:1 under Condition A; 62.6 mg, yield: 73%, r.r. = 5.7:1 under Condition B. Colorless oil. **^{1}H NMR** (300 MHz, $CDCl_3$) δ 8.31 (s, 1H), 5.28 (s, 0.14H), 4.88 (d, *J* = 6.0 Hz, 0.85H), 4.76 (s, 0.84H), 4.46 (d, *J* = 6.0 Hz, 0.14H), 4.27-4.15 (m, 4H), 3.94 (t, *J* = 6.0 Hz, 0.17H), 3.85 (t, *J* = 6.0 Hz, 0.83H), 2.65 (br, 2H), 1.76-1.66 (m, 3H), 1.45 (s, 9H), 1.36-1.27 (m, 5H), 1.01 (s, 7.8H), 0.94 (s, 1.3H). **^{13}C NMR** (75 MHz, $CDCl_3$) δ 169.80, 162.60, 154.63, 79.54, 75.02, 63.03, 61.97, 54.08, 43.75, 40.13, 38.00, 31.10, 28.43, 28.21, 26.68, 26.29, 14.03. **HRMS (ESI)** $C_{21}H_{36}N_2NaO_5S$ $[M+Na]^+$ calcd: 451.2238, found: 451.2244.

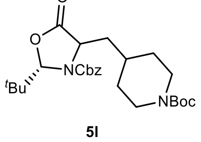

5l

benzyl (2S)-4-((1-(tert-butoxycarbonyl)piperidin-4-yl)methyl)-2-(tert-butyl)-5-oxooxazolidine-3-carboxylate: 90.1 mg, yield: 95%, > 20:1 d.r. under Condition A; 84.3 mg, yield: 89%, d.r. > 20:1 under Condition B. Colorless oil. **^{1}H NMR** (300 MHz, $CDCl_3$) δ 7.41-7.30 (m, 5H), 5.56 (s, 1H), 5.19-5.07 (m, 2H), 4.36-4.32 (m, 1H), 3.98 (br, 2H), 2.60-2.56 (m,2H), 1.86-1.59 (m, 5H), 1.46 (s, 9H), 1.08-0.96 (m, 11H). **^{13}C NMR** (75 MHz, $CDCl_3$) δ172.81, 155.92, 154.70, 135.01, 128.82, 128.76, 128.70, 114.26, 96.34, 79.19, 68.51, 40.14, 36.91, 32.75, 32.12, 31.63, 28.44, 24.91. **HRMS (ESI)** $C_{26}H_{38}N_2NaO_6$ $[M+Na]^+$ calcd:497.2628, found: 497.2628.

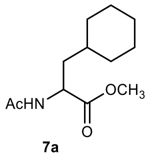

7a

methyl 2-acetamido-3-cyclohexylpropanoate: 37.7 mg, yield: 83% under Condition A; 36.2 mg, yield: 80% under Condition B. White solid. M. p. 74-76 °C. **^{1}H NMR** (300 MHz, $CDCl_3$) δ 5.89 (d, *J* = 9.0 Hz, 1H), 4.70-4.62 (m, 1H), 3.73 (s, 3H), 2.03 (s, 3H), 1.81-1.62 (m, 6H), 1.55-1.46 (m, 1H),

1.37-1.11 (m, 4H), 1.00-0.83 (m, 2H). **^{13}C NMR** (75 MHz, CDCl$_3$): 173.88, 169.88, 52.31, 50.07, 40.19, 34.07, 33.46, 32.54, 26.33, 26.16, 25.98, 23.20. **HRMS (ESI)** $C_{12}H_{21}NNaO_3$ [M+Na]$^+$ calcd: 250.1414, found: 250.1415.

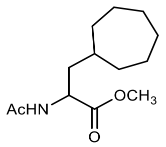

7b

methyl 2-acetamido-3-cycloheptylpropanoate: 43.4 mg, yield: 90% under Condition A; 39.0 mg, yield: 81% under Condition B. White solid. M. p. 63-65 °C. **^{1}H NMR** (300 MHz, CDCl$_3$) δ 6.05 (d, *J* = 9.0 Hz, 1H), 4.66-4.59 (m, 1H), 3.73 (s, 3H), 2.03 (s, 3H), 1.71-1.42 (m, 13H), 1.23-1.19 (m, 2H). **^{13}C NMR** (75 MHz, CDCl$_3$) δ 173.86, 169.91, 52.27, 50.59, 40.85, 35.51, 34.88, 33.58, 28.51, 28.40, 26.21, 25.99, 23.16. **HRMS (ESI)** $C_{13}H_{23}NNaO_3$ [M+Na]$^+$ calcd: 264.1571, found: 264.1571.

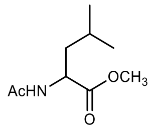

7c

methyl acetylleucinate: 29.2 mg, yield: 78% under Condition A; 28.0 mg, yield: 75% under Condition B. White solid. M. p. 72-74 °C. **^{1}H NMR** (300 MHz, CDCl$_3$) δ 5.90 (d, *J* = 6.0Hz, 1H), 4.68-4.61 (m, 1H), 3.74 (s, 3H), 2.03 (s, 3H), 1.66-1.50 (m,3H), 0.95-0.93 (m, 6H). **^{13}C NMR** (75 MHz, CDCl$_3$) δ 173.75, 169.82, 52.31, 50.67, 41.73, 24.85, 23.20, 22.79, 21.99. **HRMS (ESI)** $C_9H_{17}NNaO_3$ [M+Na]$^+$ calcd: 210.1106, found: 210.1102.

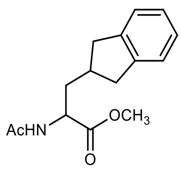

7d

methyl 2-acetamido-3-(2,3-dihydro-1H-inden-2-yl)propanoate: 42.8 mg, yield: 82% under Condition A; 39.8 mg, yield: 76% under Condition B. White solid. M. p. 71-72 °C. **^{1}H NMR** (300 MHz, CDCl$_3$) δ 7.19-7.11 (m, 4H), 6.09 (d, *J* = 9.0 Hz, 1H), 4.77-4.69 (m, 1H), 3.75 (s, 3H), 3.14-3.03 (m, 2H), 2.69-2.55 (m, 2H), 2.53-2.45 (m, 1H), 2.13-2.00 (m, 4H), 1.91-1.80 (m, 1H). **^{13}C NMR** (75 MHz, CDCl$_3$) δ 173.40, 169.83, 142.75, 126.26, 124.47, 124.32, 52.43, 51.41, 39.15, 39.02, 38.53, 36.80, 23.22. **HRMS (ESI)** $C_{15}H_{19}NNaO_3$ [M+Na]$^+$ calcd: 284.1263, found: 284.1258.

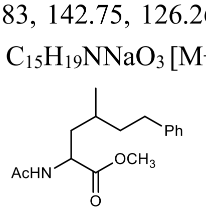

7e

methyl 2-acetamido-4-methyl-6-phenylhexanoate: 48.8 mg, yield: 88%, d.r. = 1:1 under Condition A; 47.0 mg, yield: 85%, d.r. = 1:1 under Condition B. Colorless oil. **^{1}H NMR** (300 MHz, CDCl$_3$) δ 7.30-7.24 (m, 2H), 7.19-7.15 (m, 3H), 6.10 (d, *J* = 6.0 Hz, 0.50H), 6.02 (d, *J* = 9.0 Hz, 0.45H), 4.69-4.62 (m, 1H), 3.69 (s, 3H), 2.66-2.53 (m, 2H), 1.98-1.97 (m, 3H), 1.83-1.45 (m, 5H), 1.01-0.98 (m, 3H). **^{13}C NMR** (75 MHz, CDCl$_3$) δ 173.78, 173.70, 170.03, 169.81, 142.31, 128.39, 128.36, 128.32, 125.71, 52.32, 52.27, 50.50, 50.32, 39.87, 39.66, 38.72, 37.94, 33.11, 32.98, 28.99, 23.09, 19.63, 19.12. **HRMS (ESI)** $C_{16}H_{23}NNaO_3$ [M+Na]$^+$ calcd: 300.1576, found: 300.1571.

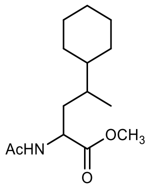

7f

methyl 2-acetamido-4-cyclohexylpentanoate: 40.8 mg, yield: 80%, d.r. = 1.2:1 under Condition A; 39.2 mg, yield: 77%, d.r. = 1.2:1 under Condition B. Colorless oil. **^{1}H NMR** (300 MHz, $CDCl_3$) δ 5.93 (d, J = 6.0 Hz, 0.51H), 5.80 (d, J = 9.0 Hz, 0.40H), 4.68-4.57 (m, 1H), 3.74 (s, 3H), 2.02 (m, 3H), 1.90-1.58 (m, 6H), 1.46-1.31 (m, 2H), 1.25-1.11 (m, 4H), 1.08-0.96 (m, 2H), 0.91-0.86 (m, 3H). **^{13}C NMR** (75 MHz, $CDCl_3$): 173.94, 173.73, 170.00, 169.60, 52.32, 52.24, 51.02, 50.56, 43.12, 42.12, 37.38, 37.25, 34.48, 30.49, 30.23, 28.81, 27.98, 26.78, 26.72, 26.66, 26.61, 23.21, 16.18, 15.71. **HRMS (ESI)** $C_{14}H_{25}NNaO_3$ $[M+Na]^+$ calcd: 278.1732, found: 278.1727.

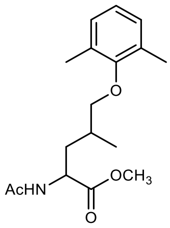

7g

methyl 2-acetamido-5-(2,6-dimethylphenoxy)-4-methylpentanoate: 47.9 mg, yield: 78%, d.r. =1:1 under Condition A; 44.1 mg, yield: 72%, d.r. = 1:1 under Condition B. White solid. M. p. 61-63 °C. **^{1}H NMR** (300 MHz, $CDCl_3$) δ 7.01-6.89 (m, 3H), 6.35 (d, J = 6.0 Hz, 0.48H), 6.18 (d, J = 9.0 Hz, 0.48H), 4.79-4.70 (m, 1H), 3.76-3.75 (m, 3H), 3.64-3.57 (m, 2H), 2.26 (d, J = 6.0 Hz, 6H), 2.21-1.93 (m, 5H), 1.83-1.64 (m,1H), 1.14 (t, J = 7.5 Hz, 3H). **^{13}C NMR** (75 MHz, $CDCl_3$) δ 173.52, 173.34, 170.14, 169.83, 155.42, 130.84, 130.76, 128.91, 123.89, 75.76, 52.44, 52.38, 50.60, 50.35, 36.64, 31.22, 31.07, 23.20, 17.59, 16.51, 16.32. **HRMS (ESI)** $C_{17}H_{25}NNaO_4$ $[M+Na]^+$ calcd: 330.1681, found: 330.1680.

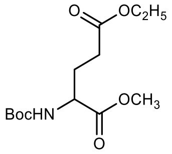

7h

5-ethyl 1-methyl (tert-butoxycarbonyl)glutamate: 53.2 mg, yield: 92% under Condition A; 49.3 mg, yield: 85% under Condition B. Yellow solid. M. p. 46-47 °C. **^{1}H NMR** (300MHz, $CDCl_3$) δ 5.19 (d, J = 6.0 Hz, 1H), 4.37- 4.30 (m, 1H), 4.14 (q, J = 6.0 Hz, 2H), 3.75 (s, 3H), 2.44-2.37 (m, 2H), 2.34-2.12 (m, 1H), 2.01-1.89 (m,1H), 1.44 (s, 9H), 1.26 (t, J = 6.0 Hz, 3H). **^{13}C NMR** (75 MHz, $CDCl_3$) δ 172.69, 155.33, 79.94, 60.60, 52.86, 52.36, 30.30, 28.25, 27.67, 14.14. **HRMS (ESI)** $C_{13}H_{23}NNaO_6$ $[M+Na]^+$ calcd: 312.1423, found:312.1419.

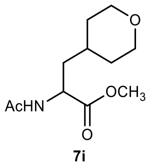

7i

methyl 2-acetamido-3-(tetrahydro-2H-pyran-4-yl)propanoate: 38.9 mg, yield: 85% under Condition A; 38.5 mg, yield: 84% under Condition B. White solid. M.p. 89-90 °C. **^{1}H NMR** (300 MHz, $CDCl_3$) δ 6.18 (d, *J* = 8.1 Hz, 1H), 4.73-4.68 (m, 1H), 3.97-3.92 (m, 2H), 3.75 (s, 3H), 3.41-3.31 (m, 2H), 2.03 (s, 3H), 1.76-1.70 (m,2H), 1.60-1.52 (m,3H), 1.36-1.26 (m, 2H). **^{13}C NMR** (75 MHz, $CDCl_3$) δ173.49, 169.94, 67.79, 67.72, 52.39, 49.52, 39.89, 33.07, 32.33, 31.58, 23.12. **HRMS (ESI)** $C_{11}H_{19}NNaO_4$ $[M+Na]^+$ calcd: 252.1212, found: 252.1209.

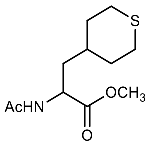

7j

methyl 2-acetamido-3-(tetrahydro-2H-thiopyran-4-yl)propanoate: 38.0 mg, yield: 78% under Condition A; 38.1 mg, yield: 78% under Condition B. Yellow solid. M. p. 91-92 °C. **^{1}H NMR** (300 MHz, $CDCl_3$) δ 5.99 (d, *J* = 7.8Hz, 1H), 4.73-4.66 (m, 1H), 3.74 (s, 3H), 2.69-2.56 (m, 4H), 2.18-2.10 (m, 1H), 2.03 (s, 3H), 1.98-1.95 (m, 1H), 1.76-1.68 (m, 1H), 1.55-1.31 (m, 4H). **^{13}C NMR** (75 MHz, $CDCl_3$) δ 173.51, 169.85, 52.46, 49.46, 40.44, 34.35, 33.71, 33.35, 28.49, 28.43, 23.23. **HRMS (ESI)** $C_{11}H_{20}NO_3S$ $[M+H]^+$ calcd: 246.1164, found: 246.1162.

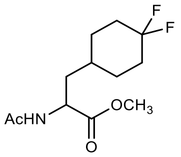

7k

methyl 2-acetamido-3-(4,4-difluorocyclohexyl)propanoate: 39.2 mg, yield: 75% under Condition A; 37.0 mg, yield: 71%, under Condition B. Colorless oil. **^{1}H NMR** (300 MHz, $CDCl_3$) δ 5.98 (d, *J* = 8.1Hz, 1H), 4.72-4.64 (m, 1H), 3.75 (s, 3H), 2.12-2.04 (m, 5H), 1.96-1.91 (m, 1H), 1.80-1.58 (m, 5H), 1.45-1.38 (m, 1H), 1.33-1.29 (m, 2H). **^{19}F NMR** (282 MHz, $CDCl_3$) δ -92.00 (d, *J* = 234 Hz), -102.11 (d, *J* = 236 Hz). **^{13}C NMR** (75 MHz, $CDCl_3$) δ 173.36, 169.92, 52.49, 50.13, 39.02, 33.26 (dd, *J* = 2.1 Hz, 12.2 Hz), 33.25 (dd, *J* = 12.1 Hz, 48.3 Hz), 32.28, 28.71 (dd, *J* = 9.5 Hz, 67.2 Hz), 23.20. **HRMS (ESI)** $C_{12}H_{19}F_2NNaO_3$ $[M+Na]^+$ calcd: 286.1231, found: 286.1227.

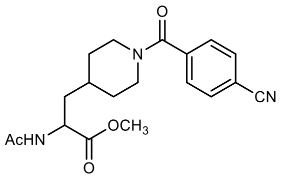

7l

methyl 2-acetamido-3-(1-(4-cyanobenzoyl)piperidin-4-yl)propanoate: 32.0 mg, yield: 45% under Condition A; 30.0 mg, yield: 42% under Condition B. Yellow oil. **^{1}H NMR** (300 MHz, $CDCl_3$) δ 7.71 (d, *J* = 8.4 Hz, 2H), 7.49 (d, *J* = 8.4 Hz, 2H), 5.99 (d, *J* = 8.4 Hz, 1H), 4.74-4.67 (m, 2H), 3.76 (s, 3H), 3.62-3.57 (m, 1H), 3.05-3.01 (m, 1H), 2.81-2.73 (m, 1H), 2.04 (s, 3H), 1.92-1.88 (m, 1H), 1.79-1.57 (m, 6H). **^{13}C NMR** (75 MHz, $CDCl_3$) δ 173.20, 169.94, 168.19, 140.56, 132.45, 127.56, 118.15, 113.39, 52.60, 49.66, 47.74, 42.34, 39.79, 32.67, 32.06, 29.68, 23.24. **HRMS (ESI)** $C_{19}H_{23}N_3NaO_4$ $[M+Na]^+$ calcd: 380.1586, found: 380.1589.

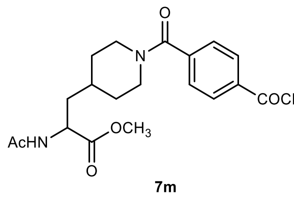

7m

methyl 2-acetamido-3-(1-(4-acetylbenzoyl)piperidin-4-yl)propanoate: 43.4 mg, yield: 58% under Condition A; 42.5 mg, yield: 57% under Condition B. Colorless oil. **^{1}H NMR** (300 MHz, $CDCl_3$) δ 7.99 (d, *J* = 8.1 Hz, 2H), 7.47 (d, *J* = 8.1 Hz, 2H), 6.01 (d, *J* = 8.1 Hz, 1H), 4.73-4.69 (m, 2H), 3.75 (s, 3H), 3.66-3.63 (m, 1H), 3.03-2.96 (m, 1H), 2.84-2.73 (m, 1H), 2.63 (m, 3H), 2.03-1.97 (m, 4H), 1.89-1.63 (m, 6H). **^{13}C NMR** (75 MHz, $CDCl_3$) δ 197.43, 173.24, 169.92, 169.13, 140.65, 137.67, 128.53, 127.02, 52.56, 49.68, 47.67, 42.27, 39.73, 32.72, 32.09, 29.70, 26.73, 23.24. **HRMS (ESI)** $C_{20}H_{26}N_2NaO_5$ $[M+Na]^+$ calcd: 397.1739, found: 397.1737.

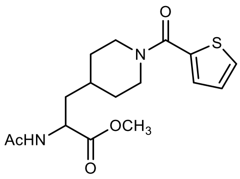

7n

methyl 2-acetamido-3-(1-(thiophene-2-carbonyl)piperidin-4-yl)propanoate: 45.9 mg, yield: 68% under Condition A; 44.4 mg, yield: 66% under Condition B. White solid. M. p. 67-68 °C. **^{1}H NMR** (300 MHz, $CDCl_3$) δ 7.43 (d, *J* = 4.8 Hz, 1H), 7.27 (d, *J* = 3.9 Hz, 1H), 7.27 (d, *J* = 3.9 Hz, 1H), 6.17 (d, *J* = 8.4 Hz, 1H), 4.71-4.68 (m, 1H), 4.27 (br, 2H), 3.75 (s, 3H), 2.93 (br, 2H), 2.04 (s, 3H), 1.97-1.90 (m, 2H), 1.78-1.55 (m, 5H). **^{13}C NMR** (75 MHz, $CDCl_3$) δ 173.30, 169.96, 163.48, 137.32, 128.49, 128.32, 126.63, 52.53, 49.71, 39.63, 32.80, 32.50, 31.82, 23.22. **HRMS (ESI)** $C_{16}H_{22}N_2NaO_4S$ $[M+Na]^+$ calcd: 361.1198, found: 361.1192.

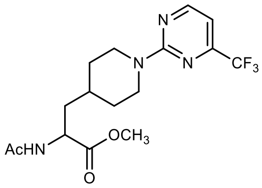

7o

methyl 2-acetamido-3-(1-(4-(trifluoromethyl)pyrimidin-2-yl)piperidin-4-yl)propanoate: 41.0 mg, yield: 55% under Condition A; 40.4 mg, yield: 54% under Condition B. Colorless oil. **^{1}H NMR** (300 MHz, $CDCl_3$) δ 8.46 (d, *J* = 4.8 Hz, 1H),6.70 (d, *J* = 7.8 Hz, 1H), 5.99 (d, *J* = 8.1 Hz, 1H), 4.80-4.69 (m, 3H), 3.75 (s, 3H), 2.92 (t, *J* = 24 Hz, 2H), 2.05 (m, 3H), 2.00-1.91 (m, 1H), 1.86-1.55 (m, 6H). **^{19}F NMR** (282 MHz, $CDCl_3$) δ -70.90. **^{13}C NMR** (75 MHz, $CDCl_3$) δ 173.46, 169.89, 161.32, 160.02, 156.47, 156.00,104.00, 52.49, 49.85, 43.87, 39.73, 32.85, 32.11, 31.41, 29.70, 23.26. **HRMS (ESI)** $C_{16}H_{21}F_3N_4NaO_3$ $[M+Na]^+$ calcd: 397.1463, found: 397.1463.

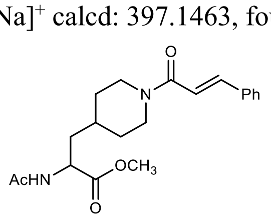

7p

methyl 2-acetamido-3-(1-cinnamoylpiperidin-4-yl)propanoate: 45.0 mg, yield: 63% under Condition A; 40.8 mg, yield: 57% under Condition B. Yellow oil. **^{1}H NMR** (300 MHz, $CDCl_3$) δ

7.65 (d, J = 15.6 Hz, 1H), 7.53-7.51 (m, 2H). 7.38-7.34 (m, 3H), 6.89 (d, J = 15.3 Hz, 1H), 6.09 (d, J = 8.4 Hz, 1H), 4.72-4.69 (m, 2H), 4.12-4.08 (m, 1H), 3.75 (s, 3H), 3.09-2.96 (m, 1H), 2.70-2.62 (m, 1H), 2.05-2.00 (m, 4H), 1.83-1.54 (m, 6H). **^{13}C NMR** (75 MHz, $CDCl_3$) δ 173.30, 170.05, 165.37, 142.52, 135.34, 129.54, 128.79, 127.72, 117.42, 52.52, 49.74, 45.99, 42.35, 39.63, 32.76, 23.22. **HRMS (ESI)** $C_{20}H_{26}N_2NaO_4$ $[M+Na]^+$ calcd: 381.1790, found: 381.1790.

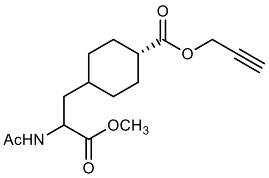

7q

prop-2-yn-1-yl 4-(2-acetamido-3-methoxy-3-oxopropyl)cyclohexane-1-carboxylate: 49.5 mg, yield: 80%, d.r. = 1.5:1 under Condition A; 48.1 mg, yield: 78%, d.r. = 1.5:1 under Condition B. Colorless oil. **^{1}H NMR** (300 MHz, $CDCl_3$) δ 6.01 (d, J = 7.8 Hz, 1H), 4.71-4.60 (m, 3H), 3.73 (s, 3H), 2.61-2.57 (m, 0.6H), 2.47 (t, J = 2.4 Hz, 1H), 2.34-2.23 (m, 0.4H), 2.03-1.97 (m, 5H), 1.81-1.27 (m, 8H), 1.07-0.88 (m, 1H). **^{13}C NMR** (75 MHz, $CDCl_3$) δ174.97, 174.41, 173.65, 169.90, 77.86, 74.75, 74.67, 52.36, 51.81, 51.75, 50.13, 49.94, 42.90, 40.02, 39.95, 33.39, 32.16, 31.41, 29.55, 28.56, 28.44, 25.96, 25.80, 23.16. **HRMS (ESI)** $C_{16}H_{23}NNaO_5$ [M+Na]+ calcd:332.1468, found: 332.1472.

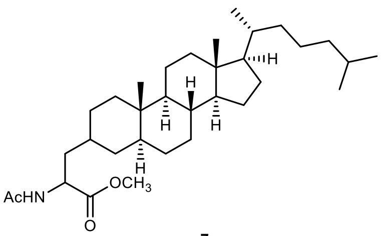

7r

methyl 2-acetamido-3-((5S,8R,9S,10S,13R,14S,17R)-10,13-dimethyl-17-((R)-6-methylheptan-2-yl)hexadecahydro-1H-cyclopenta[a]phenanthren-3-yl)propanoate: 90.7 mg, yield: 88% under Condition A; 84.4 mg, yield: 82% under Condition B, d.r. = 10.9:2.6:1.4:1 under Condition A, d.r. = 10.9:2.6:1.4:1 under Condition B (d.r. were measured by HPLC, IA, hexane : ethanol = 4:1). Yellow oil. **^{1}H NMR** (300 MHz, $CDCl_3$) δ 6.19-6.11 (m, 1H), 4.68-4.53 (m, 1H), 3.73 (s, 3H), 2.04-1.94 (m, 5H), 1.84-1.73 (m,2H), 1.55-1.45 (m, 7H), 1.34-0.91(m, 21H), 0.89-0.85 (m, 12H), 0.77 (s, 2H), 0.70 (s, 3H). **^{13}C NMR** (75 MHz, $CDCl_3$) δ173.88, 169.89, 56.59, 56.29, 54.62, 52.26, 50.98, 50.92, 42.58, 40.61, 40.04, 39.50, 36.38, 36.17, 35.81, 35.47, 35.00, 33.04, 32.05, 29.27, 28.90, 28.24, 27.99, 26.90, 24.15, 23.86, 23.11, 22.82, 22.56, 20.76, 18.65, 12.06, 11.69. **HRMS (ESI)** $C_{33}H_{57}NNaO_3$ $[M+Na]^+$ calcd:538.4231, found: 538.4225.

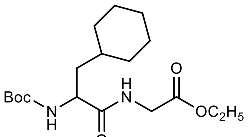

9a

ethyl (2-((tert-butoxycarbonyl)amino)-3-cyclohexylpropanoyl)glycinate: 64.8 mg, yield: 91% under Condition A; 59.3 mg, yield: 83% under Condition B. White solid. M. p. 120-125 °C. **^{1}H NMR** (300 MHz, $CDCl_3$) δ 6.85 (br, 1H), 5.04 (d, J = 9.0 Hz, 1H), 4.24-4.17 (m, 3H), 4.03-4.01 (m, 2H), 1.82-1.76 (m, 1H), 1.74-1.66 (m, 5H), 1.52-1.47 (m, 1H), 1.45 (s, 9H), 1.42-1.36 (m, 1H),

1.28 (t, J = 6.0 Hz, 3H), 1.23-1.15 (m, 3H), 1.02-0.86 (m, 2H). **^{13}C NMR** (75 MHz, $CDCl_3$) δ 173.04, 169.70, 155.74, 80.05, 61.43, 52.20, 41.27, 39.96, 34.01, 33.67, 32.48, 28.28, 26.39, 26.22, 26.04, 14.12. **HRMS (ESI)** $C_{18}H_{32}N_2NaO_5$ $[M+Na]^+$ calcd: 379.2209, found:379.2208.

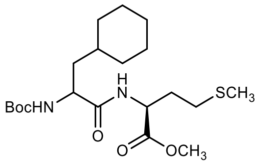

9b

methyl (2-((tert-butoxycarbonyl)amino)-3-cyclohexylpropanoyl)-*L*-methioninate: 74.9 mg, yield: 90%, d.r. = 1.5:1 under Condition A; 67.6 mg, yield: 81%, d.r. = 1.5:1 under Condition B. White solid. M. p. 72-75 °C. **^{1}H NMR** (300 MHz, $CDCl_3$) δ 7.06 (d, J = 6.0Hz, 0.38H), 6.90 (d, J = 9.0 Hz, 0.59H), 5.06 (d, J = 6.0 Hz, 1H), 4.74-4.67 (m, 1H), 4.20-4.14 (m, 1H), 3.75-3.74 (m, 3H), 2.51 (t, J = 6.0 Hz, 2H), 2.20-2.14 (m, 1H), 2.09 (s, 3H), 2.08-1.95 (m, 1H), 1.80-1.63 (m, 6H), 1.50-1.42 (m, 10H), 1.36-1.29 (m, 1H), 1.26-1.11 (m, 3H), 1.01-0.87 (m, 2H). **^{13}C NMR** (75 MHz, $CDCl_3$) δ 172.58, 172.20, 172.11, 155.69, 80.02, 52.46, 51.35, 39.68, 34.08, 33.95, 33.60, 32.66, 32.51, 31.60, 31.47, 29.84, 29.80, 28.27, 26.36, 26.22, 26.18, 26.04, 15.38. **HRMS (ESI)** $C_{20}H_{36}N_2NaO_5S$ $[M+Na]^+$ calcd: 439.2243, found: 439.2247.

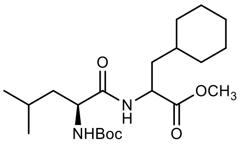

9c

Methyl 2-((*S*)-2-((tert-butoxycarbonyl)amino)-4-methylpentanamido)-3-cyclohexylpropanoate: 74.9 mg, yield: 94%, d.r. = 1.1:1 under Condition A; 71.7 mg, yield: 90%, d.r. = 2:1 under Condition B. white solid. M. p. 60-61 °C. Condition A: **^{1}H NMR** (300MHz, $CDCl_3$) δ 6.90 (d, J = 9.0 Hz, 0.41H), 6.71 (d, J = 6.0 Hz, 0.44H), 5.13 (br, 1H), 4.66-4.59 (m, 1H), 4.17 (br, 1H), 3.71 (s, 3H), 1.80-1.61 (m, 8H), 1.59-1.49 (m, 2H), 1.45 (s, 9H), 1.35-1.28 (m, 1H), 1.23-1.10 (m, 3H), 0.96-0.82 (m, 8H). **^{13}C NMR** (75 MHz, $CDCl_3$) δ 173.39, 173.26, 172.43, 155.62, 79.84, 52.88, 52.17, 49.91, 41.09, 39.88, 39.70, 33.99, 33.90, 33.48, 33.39, 32.36, 32.27, 28.26, 26.30, 26.11, 26.06, 25.91, 24.73, 24.58, 22.83, 22.12. Condition B: **^{1}H NMR** (300MHz, $CDCl_3$) δ 6.76 (d, J = 6.0 Hz, 0.61H), 6.57 (d, J = 7.8 Hz, 0.29H), 5.04-5.02 (m, 1H), 4.66-4.58 (m, 1H), 4.16 (br, 1H), 3.72 (s, 3H), 1.80-1.51 (m, 10H), 1.45 (s, 9H), 1.24-1.14 (m, 3H), 0.96-0.85 (m, 9H). **^{13}C NMR** (75 MHz, $CDCl_3$) δ 173.42, 173.31, 172.42, 155.67, 80.09, 52.96, 52.25, 49.98, 41.04, 39.83, 35.42, 34.05, 33.52, 33.35, 32.35, 28.30, 26.34, 26.16, 25.97, 24.79, 24.64, 22.89, 22.04. **HRMS (ESI)** $C_{21}H_{38}N_2NaO_5$ $[M+Na]^+$ calcd: 421.2678, found:421.2676.

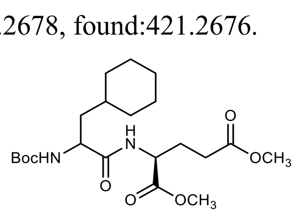

9d

dimethyl (2-((tert-butoxycarbonyl)amino)-3-cyclohexylpropanoyl)-*L*-glutamate: 77.1 mg, yield: 90%, d.r. = 1.5:1 under Condition A; 75.5 mg, yield: 88%, d.r. = 1:1 under Condition B. White solid. M. p. 99-101 °C. Condition A: **^{1}H NMR** (300 MHz, $CDCl_3$) δ 7.05 (d, J = 6.3Hz, 0.39 H), 6.91 (d, J = 6.0 Hz, 0.57H), 5.06 (d, J = 6.0 Hz, 1H), 4.64-4.58 (m, 1H), 4.20-4.15 (m, 1H), 3.75-

3.74 (m, 3H), 3.68-3.67 (m, 3H), 2.47-2.37 (m, 2H), 2.31-2.19 (m, 1H), 2.03-1.95 (m, 1H), 1.80-1.62 (m,6H), 1.51-1.41 (m, 10H), 1.36-1.31 (m, 1H), 1.26-1.15 (m, 3H), 0.97-0.86 (m, 2H). **^{13}C NMR** (75 MHz, $CDCl_3$) δ 173.16, 173.06, 172.71, 172.07, 171.97, 155.66, 79.97, 52.45, 51.76, 51.74, 51.42, 39.71, 34.06, 33.94, 33.64, 33.56, 32.63, 29.83, 28.23, 27.24, 27.18, 26.36, 26.18, 26.02. Condition B: **^{1}H NMR** (300 MHz, $CDCl_3$) δ 6.97 (d, *J* = 6.0Hz, 0.47 H), 6.84 (d, *J* = 7.8 Hz, 0.49H), 5.00-4.98 (m, 1H), 4.65-4.56 (m, 1H), 4.19-4.14 (m, 1H), 3.75 (m, 3H), 3.68 (s, 3H), 2.49-2.38 (m, 2H), 2.33-2.17 (m, 1H), 2.05-1.92 (m, 1H), 1.80-1.62 (m, 6H), 1.45-1.44 (m, 10H), 1.33-1.15 (m, 4H), 1.01-0.83 (m, 2H). **^{13}C NMR** (75 MHz, $CDCl_3$) δ 173.18, 173.09, 172.74, 171.97, 155.67, 80.05, 52.47, 51.79, 51.44, 39.68, 33.95, 33.66, 32.63, 32.48, 29.87, 28.24, 27.28, 27.21, 26.37, 26.19, 26.03. **HRMS (ESI)** $C_{21}H_{36}N_2NaO_7$ $[M+Na]^+$ calcd: 451.2420, found: 451.2424.

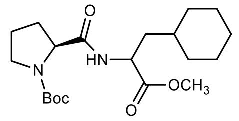

9e

tert-butyl (2*S*)-2-((3-cyclohexyl-1-methoxy-1-oxopropan-2-yl)carbamoyl)pyrrolidine-1-carboxylate: 67.3 mg, yield: 88% d.r. = 1:1 under Condition A; 67.4 mg, yield: 88%, d.r. = 1:1 under Condition B. White solid. M. p. 92-95 °C. **^{1}H NMR** (300 MHz, $CDCl_3$) δ 7.32 (s, 0.22H), 7.12 (s, 0.21H), 6.38 (s, 0.42H), 4.59 (br, 1H), 4.26 (br, 1H), 3.67 (s, 3H), 3.42-3.20 (m, 2H), 2.29-2.10 (m, 2H), 1.86-1.74 (m,3H), 1.62-1.60 (m, 6H), 1.43 (s, 9H), 1.29-1.11 (m, 4H), 0.90-0.83 (m, 2H). **^{13}C NMR** (75 MHz, $CDCl_3$) δ 175.05, 174.86, 173.53, 157.60, 82.15, 62.75, 53.81, 51.51, 48.78, 48.69, 41.66, 35.82, 35.56, 35.18, 34.05, 32.72, 30.04, 28.01, 27.84, 27.71, 27.57, 26.28, 25.27. **HRMS (ESI)** $C_{20}H_{34}N_2NaO_5$ $[M+Na]^+$ calcd: 405.2365, found: 405.2365.

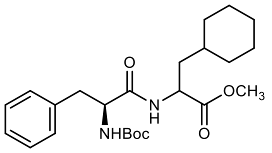

9f

methyl 2-((*S*)-2-((tert-butoxycarbonyl)amino)-3-phenylpropanamido)-3-cyclohexylpropanoate: 73.5 mg, yield: 85% under Condition A; 66.5 mg, yield: 77% under Condition B, d.r. = 1.2:1 under Condition A, d.r. = 1.2:1 under Condition B (d.r. were measured by HPLC, IA, hexane : ethanol = 2:1). Colorless oil. **^{1}H NMR** (300 MHz, $CDCl_3$) δ 7.31-7.20 (m, 5H), 6.47-6.41 (m, 1H), 5.18-5.12 (m, 1H), 4.63-4.55 (m, 1H), 4.42-4.40 (br, 1H), 3.69 (s, 3H), 3.07 (d, *J* = 6.0 Hz, 2H), 1.77-1.55 (m, 6H), 1.53-1.46 (m, 1H), 1.40 (s, 9H), 1.26-1.05 (m, 4H), 0.90-0.81 (m, 2H). **^{13}C NMR** (75 MHz, $CDCl_3$) δ 173.21, 172.97, 171.14, 155.38, 136.68, 129.39, 129.28, 128.63, 126.83, 80.09, 55.61, 52.25, 50.13, 50.02, 40.06, 39.84, 38.47, 38.15, 33.83, 33.73, 33.36, 32.44, 32.33, 28.24, 26.31, 26.06, 26.02, 25.93, 25.85. **HRMS (ESI)** $C_{24}H_{36}N_2NaO_5$ $[M+Na]^+$ calcd: 455.2517, found:455.2520.

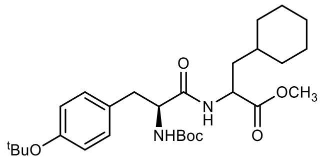

9g

methyl 2-((*S*)-3-(4-(tert-butoxy)phenyl)-2-((tert-butoxycarbonyl)amino)propanamido)-3-cyclohexylpropanoate. 95.8 mg, yield: 95%, d.r. = 1.2:1 under Condition A; 85.7 mg, yield: 85%,

d.r. = 1.7:1 under Condition B. Colorless oil. Condition A: **^{1}H NMR** (300MHz, $CDCl_3$) δ 7.10 (d, *J* = 9.0 Hz, 2H), 6.91 (d, *J* = 9.0 Hz, 2H), 6.62 (d, *J* = 6.0Hz, 0.55H), 6.53 (d, *J* = 9.0 Hz, 0.45H), 5.27-5.21 (m, 1H), 4.62-4.55 (m, 1H), 4.40-4.38 (m,1H), 3.69 (s, 3H), 3.11-2.93 (m, 2H), 1.77-1.55 (m, 6H), 1.50-1.45 (m, 1H), 1.39 (s, 9H), 1.32 (s, 9H), 1.24-1.10 (m, 4H), 0.95-0.84 (m, 2H). **^{13}C NMR** (75 MHz, $CDCl_3$) δ 173.18, 172.95, 171.29, 171.21, 155.39, 154.20, 131.49, 129.77, 129.67, 124.15, 79.91, 78.22, 55.59, 52.18, 50.11, 50.01, 39.98, 39.87, 37.73, 37.48, 33.86, 33.33, 33.30, 32.44, 32.37, 28.79, 28.22, 26.29, 26.01, 25.89, 25.85. Condition B: **^{1}H NMR** (300MHz, $CDCl_3$) δ 7.11 (d, *J* = 8.4 Hz, 2H), 6.92 (d, *J* = 8.1 Hz, 2H), 6.31-6.25 (m, 1H), 4.62-4.54 (m, 1H), 4.36-4.29 (m,1H), 3.70 (s, 3H), 3.09-2.95 (m, 2H), 1.71-1.57 (m, 6H), 1.55-1.46 (m, 1H), 1.42(s, 9H), 1.33 (s, 9H), 1.28-1.10 (m, 4H), 0.95-0.84 (m, 2H). **^{13}C NMR** (75 MHz, $CDCl_3$) δ 173.14, 172.94, 171.11, 154.34, 131.38, 129.80, 129.68, 124.29, 80.19, 78.37, 52.26, 50.16, 50.06, 39.98, 40.13, 33.89, 33.35, 32.45, 28.83, 28.25, 26.32, 26.04, 25.90. **HRMS (ESI)** $C_{28}H_{44}N_2NaO_6$ $[M+Na]^+$ calcd: 527.3097, found: 527.3102.

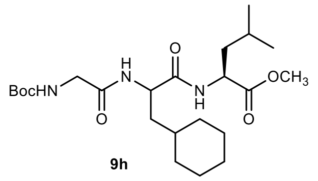

9h

methyl (2-(2-((tert-butoxycarbonyl)amino)acetamido)-3-cyclohexylpropanoyl)-*L*-leucinate: 75.6 mg, yield: 83% under Condition A; 73.9 mg, yield: 81% under Condition B, d.r. = 4:1 under Condition A, d.r. = 4:1 under Condition B (d.r. were measured by HPLC, IA, hexane : ethanol = 4:1). White solid. M. p. 97-99 °C. **^{1}H NMR** (300MHz, $CDCl_3$) δ 6.93 (d, *J* = 9.0 Hz, 1H), 6.70 (d, *J* = 6.0 Hz, 1H), 5.32 (s, 1H), 4.61-4.54 (m, 2H), 3.84-3.82 (m, 2H), 3.70 (s, 3H), 1.77-1.53 (m, 11H), 1.45 (s, 9H), 1.31-1.25 (m, 2H), 1.17-1.10 (m, 3H), 0.95-0.92 (m, 6H). **^{13}C NMR** (75 MHz, $CDCl_3$) δ 173.30, 171.94, 169.71, 156.19, 80.48, 52.28, 50.80, 44.45, 40.96, 39.31, 34.04, 33.54, 32.65, 29.70, 28.28, 26.35, 26.14, 26.02, 24.84, 22.85, 21.69. **HRMS (ESI)** $C_{23}H_{41}N_3NaO_6$ $[M+Na]^+$ calcd: 478.2893, found: 478.2899.

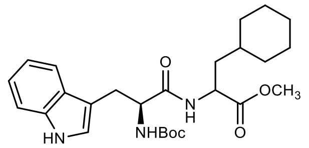

9i

methyl 2-((*S*)-2-((tert-butoxycarbonyl)amino)-3-(1H-indol-3-yl)propanamido)-3-cyclohexylpropanoate: 75.4 mg, yield: 80%, d.r. = 1:1 under Condition A; 71.6 mg, yield: 76%, d.r. = 1.7:1 under Condition B. Colorless oil. Condition A: **^{1}H NMR** (300MHz, $CDCl_3$) δ 8.49 (s, 1H), 7.63 (t, *J* = 9.0 Hz, 1H), 7.34 (d, *J* = 9.0 Hz, 1H), 7.20-7.03 (m, 3H), 6.32 (d, *J* = 9.0 Hz, 0.48 H), 6.26 (d, *J* = 9.0 Hz, 0.50H), 5.20 (br, 1H), 4.57-4.50 (m, 2H), 3.63 (s, 3H), 3.24-3.17 (m ,2H), 1.65-1.50 (m, 6H), 1.48 (s, 9H), 1.35-1.31 (m,1H), 1.21-1.10 (m, 4H), 0.88-0.77 (m, 2H). **^{13}C NMR** (75 MHz, $CDCl_3$) δ 173.19, 172.98, 171.69, 171.51, 155.50, 136.28, 127.52, 123.44, 123.17, 122.16, 119.64, 118.81, 118.72, 111.30, 110.42, 80.19, 55.15, 52.22, 52.19, 50.22, 50.11, 40.04, 33.84, 33.75, 33.27, 32.49, 32.41, 28.27, 26.30, 26.04, 26.01, 25.94, 25.87. Condition B: **^{1}H NMR** (300MHz, $CDCl_3$) δ 8.67 (s, 1H), 7.61 (d, *J* = 8.1 Hz, 1H), 7.34 (d, *J* = 7.8 Hz, 1H), 7.19-7.02 (m, 3H), 6.40 (d, *J* = 10.2 Hz, 0.64 H), 6.26 (d, *J* = 8.1 Hz, 0.37H), 5.27-5.22 (m, 1H), 4.55-4.50 (m, 2H), 3.62 (s, 3H), 3.24-3.22 (m ,2H), 1.64-1.54 (m, 6H), 1.40 (s, 9H), 1.28-1.25 (m, 1H), 1.12-1.09 (m, 4H),

0.88-0.78 (m, 2H). **^{13}C NMR** (75 MHz, $CDCl_3$) δ 173.14, 172.96, 171.52, 171.20, 155.50, 136.28, 123.33, 123.04, 122.30, 119.81, 118.91, 118.82, 111.19, 60.42, 58.49, 52.21, 50.18, 50.06, 40.12, 33.82, 33.73, 33.28, 32.42, 28.26, 26.30, 26.02, 25.88, 21.07, 14.20. **HRMS (ESI)** $C_{26}H_{37}N_3NaO_5$ $[M+Na]^+$ calcd: 494.2631, found:494.2636.

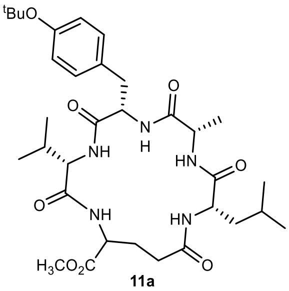

11a

methyl (2*S*,5*S*,8*S*,11*S*)-8-(4-(tert-butoxy)benzyl)-2-isobutyl-11-isopropyl-5-methyl-3,6,9,12,17-pentaoxo-1,4,7,10,13-pentaazacycloheptadecane-14-carboxylate. Prepared following the general procedure outlined using 1-((7*S*,10*S*,13*S*,16*S*)-10-(4-(tert-butoxy)benzyl)-16-isobutyl-7-isopropyl-13-methyl-4-methylene-3,6,9,12,15,18-hexaoxo-2-oxa-5,8,11,14,17-pentaazanonadecan-19-yl)-2,4,6-triphenylpyridin-1-ium tetrafluoroborate (20.8 mg, 0.02 mmol, 1.0 equiv), Et_3N (4.1 mg, 0.04 mmol, 2 equiv), K_2CO_3 (2.8 mg, 0.02 mmol, 1.0 equiv), H_2O (9.0 µL, 0.5 mmol, 25 equiv) and CH_2Cl_2 (1.0 mL). After 12 h, the reaction mixture was removed from light irradiation, purified by using Semi preparative HPLC, conditions: HPLCONE (Daiso, C_{18}, 10µ, 100Å), length 20 mm*250 mm, 30-88% CH_3CN/H_2O + 0.1% trifluoroacetic acid. The product was obtained as a white solid (3.6 mg, isolated yield: 28%), and subjected to HPLC analysis, conditions: HPLCONE (Daiso, C_{18}, 5µ, 100Å), length 4.6 mm*250 mm, 90% CH_3CN/H_2O + 0.1% trifluoroacetic acid. The d.r. = 2.4:1 under Condition A (d.r. were measured by HPLC, IA, hexane:ethanol = 2:1). **HRMS (ESI)** $C_{33}H_{51}N_5NaO_8$ $[M+Na]^+$ calcd: 668.3670, found: 668.3659.

Preparative reverse phase HPLC data for purified peptide

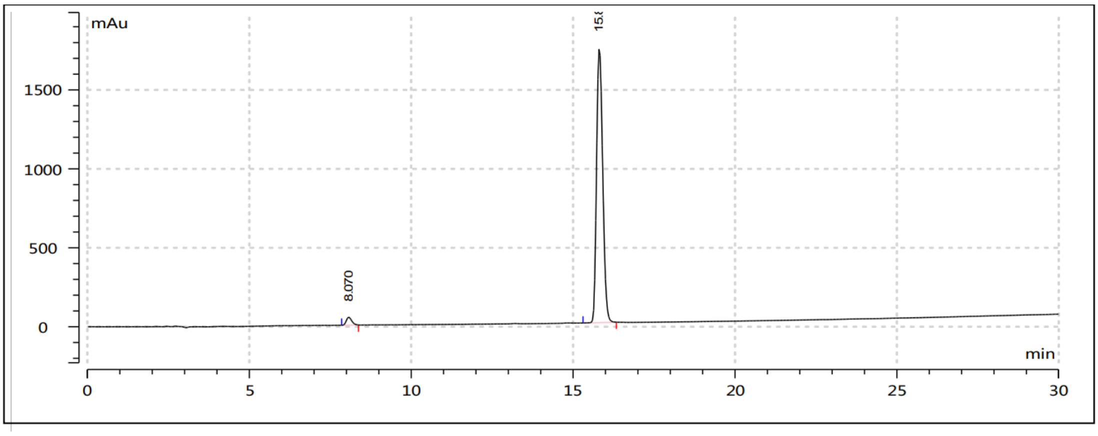

No	Ret. Time (min)	Area (mAu*min)	Rel. Area (%)	Height(mAu)
1	8.070	584.01758	2.471	50.415
2	15.808	23050.30222	97.529	1742.366

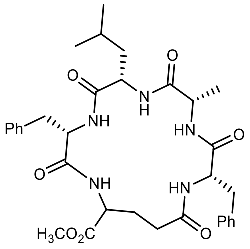

11b

methyl (2*S*,5*S*,8*S*,11*S*)-2,11-dibenzyl-8-isobutyl-5-methyl-3,6,9,12,17-pentaoxo-1,4,7,10,13-pentaazacycloheptadecane-14-carboxylate. Prepared following the general procedure outlined using 1-((7*S*,10*S*,13*S*,16*S*)-7,16-dibenzyl-10-isobutyl-13-methyl-4-methylene-3,6,9,12,15,18-hexaoxo-2-oxa-5,8,11,14,17-pentaazanonadecan-19-yl)-2,4,6-triphenylpyridin-1-ium tetrafluoroborate (50.7 mg, 0.05 mmol, 1.0 equiv), Et_3N (10.1 mg, 0.1 mmol, 2 equiv), K_2CO_3 (6.9 mg, 0.05 mmol, 1.0 equiv), H_2O (22.5 µL, 1.25 mmol, 25 equiv) and CH_2Cl_2 (2.5 mL). After 12 h, the reaction mixture was removed from light irradiation, purified by using Semi preparative HPLC, conditions: HPLCONE (Daiso, C_{18}, 10µ, 100Å), length 20 mm*250 mm, 30-88% CH_3CN/H_2O + 0.1% trifluoroacetic acid. The product was obtained as a white solid (12.4 mg, isolated yield: 40%, d.r. = 8:1), and subjected to HPLC analysis, conditions: HPLCONE (Daiso, C_{18}, 5µ, 100Å), length 4.6 mm*250 mm, 90% CH_3CN/H_2O + 0.1% trifluoroacetic acid. **^{1}H NMR** (600MHz, CD_3OD) δ 7.24-7.09 (m, 10 H), 4.46-4.44 (m, 0.12H), 4.41-4.38 (m, 0.13H), 4.36-4.34 (m, 0.88H), 4.33-4.30 (m, 0.15H), 4.30-4.27 (m, 0.87H), 4.18-4.15 (m, 0.86H), 4.08-4.05 (m, 0.12H), 3.98-3.95 (m, 0.89H), 3.66-3.63 (m, 1H), 3.60 (s, 3H), 3.30-3.24 (m, 0.32H), 3.19-3.18 (m, 0.65H), 3.13-3.09 (m, 1H), 2.97-2.95 (m ,1.81H), 2.90-2.86 (m, 0.16H), 2.37-2.29 (m, 1H), 2.27-2.22 (m, 1H), 2.13-2.07 (m, 1H), 2.01-1.96 (m, 1H), 1.75-1.70 (m, 1H), 1.54-1.50 (m ,1H), 1.48-1.41 (m, 1H), 1.21 (d, J = 6.0 Hz, 2.70H), 1.11 (d, J = 6.0 Hz, 0.35H), 0.80-0.73 (m, 6H) **^{13}C NMR** (150 MHz, CD_3OD) δ174.23, 173.18, 173.14, 172.52, 172.38, 171.86, 138.20, 136.39, 129.04, 129.01, 128.24, 128.01, 126.67, 126.17, 56.29, 56.17, 53.54, 53.22, 51.44, 50.56, 37.81, 36.81, 35.96, 31.18, 24.61, 24.45, 22.22, 20.11, 14.43. **HRMS (ESI)** $C_{33}H_{44}N_5O_7$ $[M+H]^+$ calcd: 622.3235, found: 622.3208.

Preparative reverse phase HPLC data for purified peptide

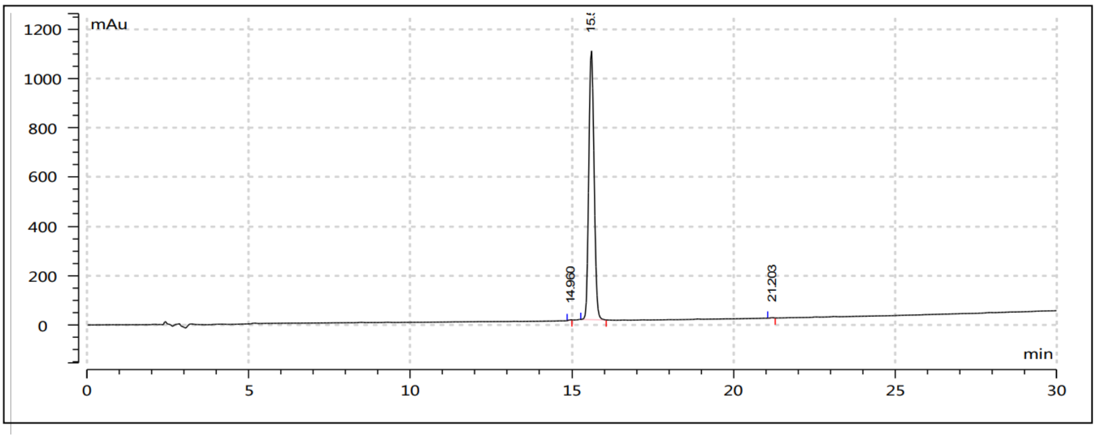

No	Ret. Time (min)	Area (mAu*min)	Rel. Area (%)	Height(mAu)
1	14,960	6.32244	0.050	0.927

2	15,590	12506.58675	99.867	1100.913
3	21.203	10.30974	0.082	1.339

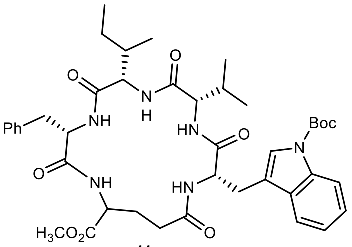

11c

tert-butyl 3-(((2*S*,5*S*,8*S*,11*S*)-11-benzyl-8-((*R*)-sec-butyl)-5-isopropyl-14-(methoxycarbonyl)-3,6,9,12,17-pentaoxo-1,4,7,10,13-pentaazacycloheptadecan-2-yl)methyl)-1H-indole-1-carboxylate. Prepared following the general procedure outlined using 1-((7*S*,10*S*,13*S*,16*S*)-7-benzyl-16-((1-(tert-butoxycarbonyl)-1H-indol-3-yl)methyl)-10-((*S*)-sec-butyl)-13-isopropyl-4-methylene-3,6,9,12,15,18-hexaoxo-2-oxa-5,8,11,14,17-pentaazanonadecan-19-yl)-2,4,6-triphenylpyridin-1-ium tetrafluoroborate (23.6 mg, 0.02 mmol, 1.0 equiv), Et_3N (4.1 mg, 0.04 mmol, 2 equiv), K_2CO_3 (2.8 mg, 0.02 mmol, 1.0 equiv), H_2O (9.0 µL, 0.5 mmol, 25 equiv), and CH_2Cl_2 (1.0 mL), After 12 h, the reaction mixture was removed from light irradiation, purified by using Semi preparative HPLC, conditions: HPLCONE (Daiso, C_{18}, 10µ, 100Å), length 20 mm*250 mm, 30-88% CH_3CN/H_2O + 0.1% trifluoroacetic acid. The product was obtained as a white solid (4.5 mg, isolated yield: 29%), and subjected to HPLC analysis, Conditions: HPLCONE (Daiso, C_{18}, 5µ, 100Å), length 4.6 mm*250 mm, 60% $MeCN/H_2O$ + 0.1% trifluoroacetic acid. The d.r. = 15.5:1 under Condition A (d.r. were measured by HPLC, IA, hexane:ethanol = 2:1). **HRMS (ESI)** $C_{42}H_{56}N_6NaO_9$ $[M+Na]^+$ calcd: 811.4001, found: 811.4014.

Preparative reverse phase HPLC data for purified peptide

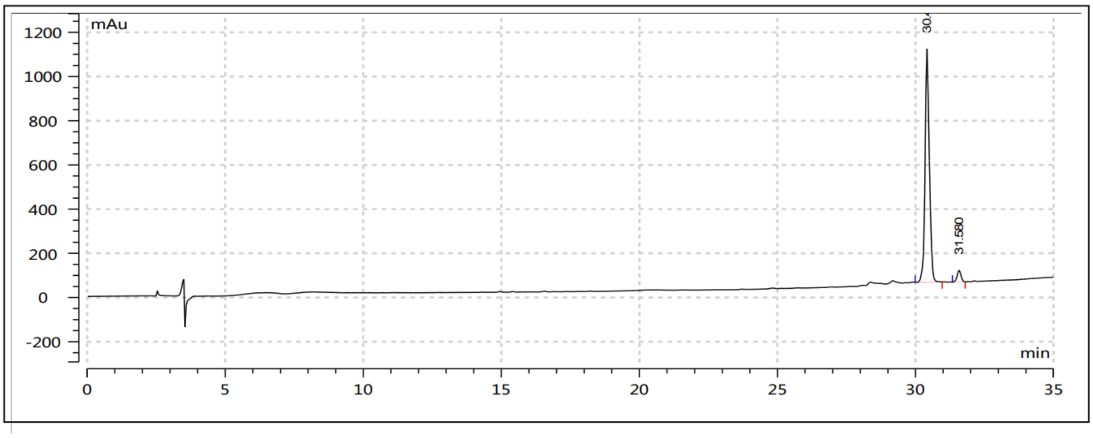

No	Ret. Time (min)	Area (mAu*min)	Rel. Area (%)	Height(mAu)
1	30.413	11328.19372	95.660	1057.441
2	31.580	513.96827	4.340	53.500

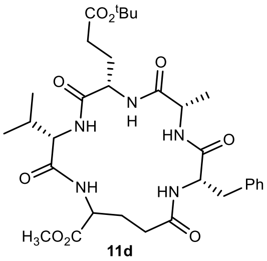

11d

methyl (2*S*,5*S*,8*S*,11*S*)-2-benzyl-8-(3-(tert-butoxy)-3-oxopropyl)-11-isopropyl-5-methyl-3,6,9,12,17-pentaoxo-1,4,7,10,13-pentaazacycloheptadecane-14-carboxylate. Prepared following the general procedure outlined using 1-((7*S*,10*S*,13*S*,16*S*)-16-benzyl-10-(3-(tert-butoxy)-3-oxopropyl)-7-isopropyl-13-methyl-4-methylene-3,6,9,12,15,18-hexaoxo-2-oxa-5,8,11,14,17-pentaazanonadecan-19-yl)-2,4,6-triphenylpyridin-1-ium tetrafluoroborate (20.8 mg, 0.02 mmol, 1.0 equiv), Et_3N (4.1 mg, 0.04 mmol, 2 equiv), K_2CO_3 (2.8 mg, 0.02 mmol, 1.0 equiv), H_2O (9.0 µL, 0.5 mmol, 25 equiv) and CH_2Cl_2 (1.0 mL), After 12 h, the reaction mixture was removed from light irradiation, purified by using Semi preparative HPLC, conditions: HPLCONE (Daiso, C_{18}, 10μ, 100Å), length 20 mm*250 mm, 30-88% CH_3CN/H_2O + 0.1% trifluoroacetic acid. The product was obtained as a white solid (3.9 mg, isolated yield: 30%), and subjected to HPLC analysis, conditions: HPLCONE (Daiso, C_{18}, 5μ, 100Å), length 4.6 mm*250 mm, 90% CH_3CN/H_2O + 0.1% trifluoroacetic acid. The d.r. >20:1 under Condition A, (d.r. were measured by HPLC, IA, hexane:ethanol = 4:1). **HRMS (ESI)** $C_{32}H_{47}N_5NaO_9$ $[M+Na]^+$ calcd: 668.3266, found: 668.3266.

Preparative reverse phase HPLC data for purified peptide

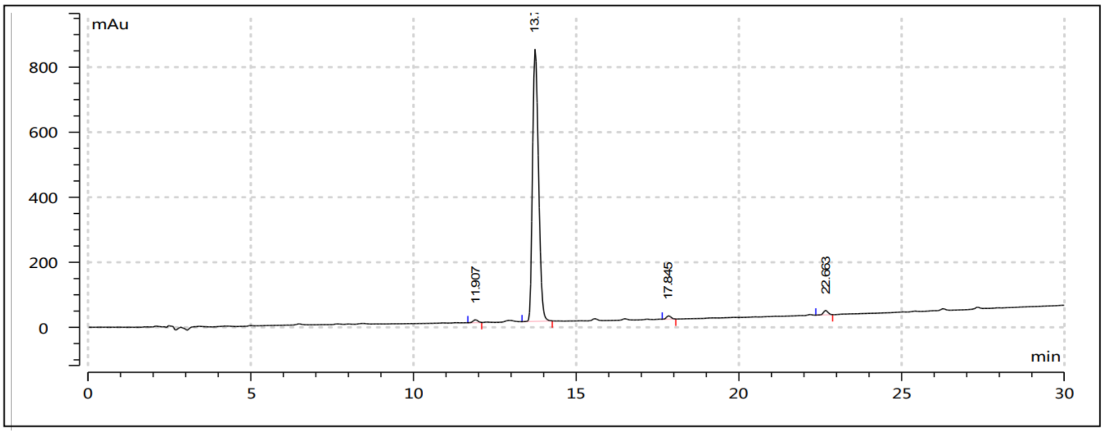

No	Ret. Time (min)	Area (mAu*min)	Rel. Area (%)	Height(mAu)
1	11.907	88.13729	0.794	8.888
2	13.738	10781.96627	97.097	838.408
3	17.845	99.14038	0.893	10.424
4	22.663	135.07195	1.216	13.896

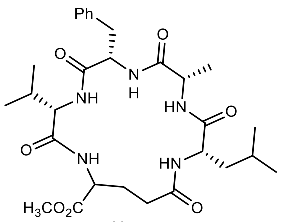

11e

methyl (2*S*,5*S*,8*S*,11*S*)-8-benzyl-2-isobutyl-11-isopropyl-5-methyl-3,6,9,12,17-pentaoxo-1,4,7,10,13-pentaazacycloheptadecane-14-carboxylate. Prepared following the general procedure outlined above using 1-((7*S*,10*S*,13*S*,16*S*)-10-benzyl-16-isobutyl-7-isopropyl-13-methyl-4-methylene-3,6,9,12,15,18-hexaoxo-2-oxa-5,8,11,14,17-pentaazanonadecan-19-yl)-2,4,6-triphenylpyridin-1-ium tetrafluoroborate (19.3 mg, 0.02 mmol, 1.0 equiv), Et_3N (4.1 mg, 0.04 mmol, 2 equiv), K_2CO_3 (2.8 mg, 0.02 mmol, 1.0 equiv), H_2O (9.0 µL, 0.5 mmol, 25 equiv), and CH_2Cl_2 (1.0 mL). After 12 h, the reaction mixture was removed from light irradiation, purified by using Semi preparative HPLC, conditions: HPLCONE (Daiso, C_{18}, 10µ, 100Å), length 20 mm*250 mm, 30-88% CH_3CN/H_2O + 0.1% trifluoroacetic acid. The product was obtained as a white solid (3.8 mg, isolated yield: 33%), and subjected to HPLC analysis, coditions: HPLCONE (Daiso, C_{18}, 5µ, 100Å), length 4.6 mm*250 mm, 90% $MeCN/H_2O$ + 0.1% trifluoroacetic acid. The d.r. = 5.6:1 under Condition A, (d.r. were measured by HPLC, IA, hexane:ethanol = 2:1). **HRMS (ESI)** $C_{29}H_{43}N_5NaO_7$ $[M+Na]^+$ calcd: 596.3055, found: 596.3059.

Preparative reverse phase HPLC data for purified peptide

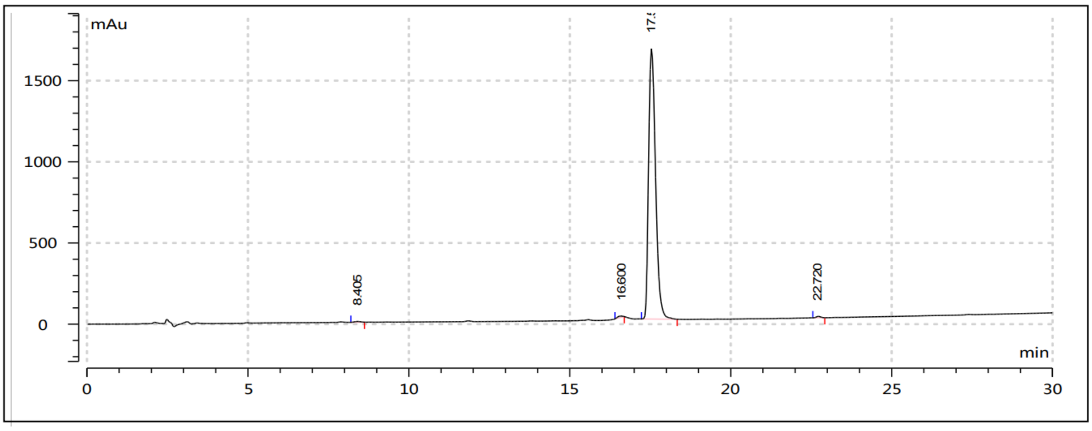

No	Ret. Time (min)	Area (mAu*min)	Rel. Area (%)	Height(mAu)
1	8.405	64.73185	0.256	5.126
2	16.600	100.03972	0.396	7.621
3	17.540	25010.50586	99.034	1667.212
4	22.720	79.30661	0.314	8.665

5. The mechanistic studies.

5.1 Radical trapping experiments

In the presence of TEMPO, the reaction was completely suppressed and the yield of **5a** was 0% (Supplementary Figure 1, eq 1 and 2).

Furthermore, when **1**, basic ionic compound and TEMPO were mixed and irradiated under 420-430 nm LED light, the adduct of alkyl radical and TEMPO was detected by HRMS (Supplementary Figure 1, eq 3). However, without addition of basic ionic compound, the alkyl radical capture product was not detected (Supplementary Figure 1, eq 4).

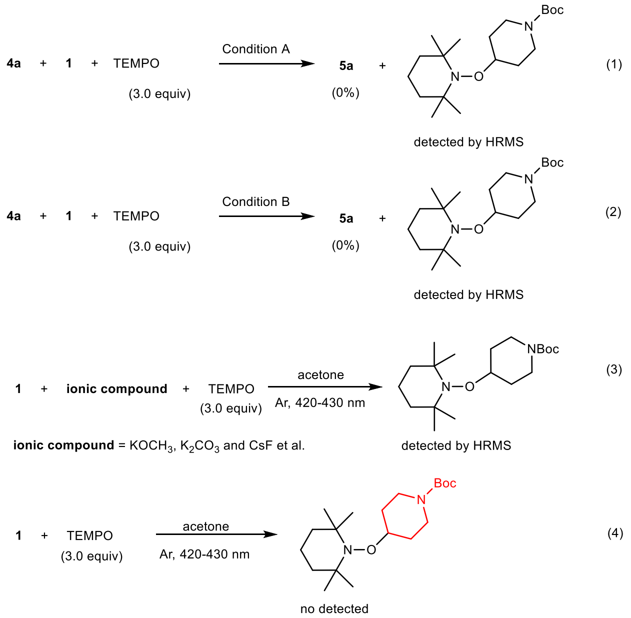

Supplementary Figure 1. Radical trapping experiments.

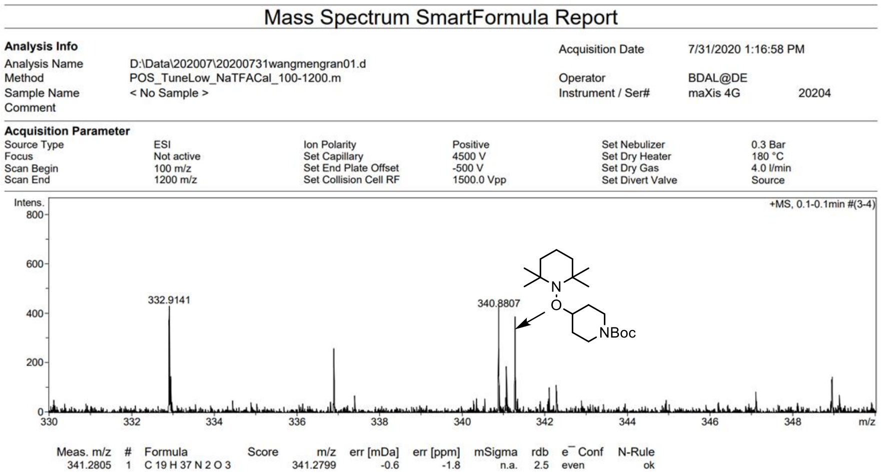

Supplementary Figure 2. The HRMS analysis of the reaction in supplementary figure 1, eq 1.

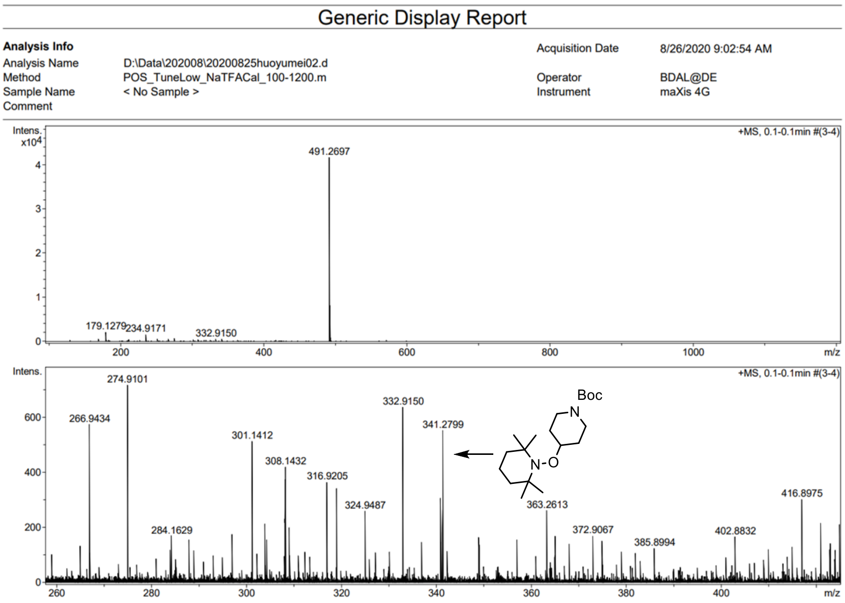

Supplementary Figure 3. The HRMS analysis of the reaction in supplementary figure 1, eq 3.

5.2 Isotope labeling experiments.

H/D labeling experiment.

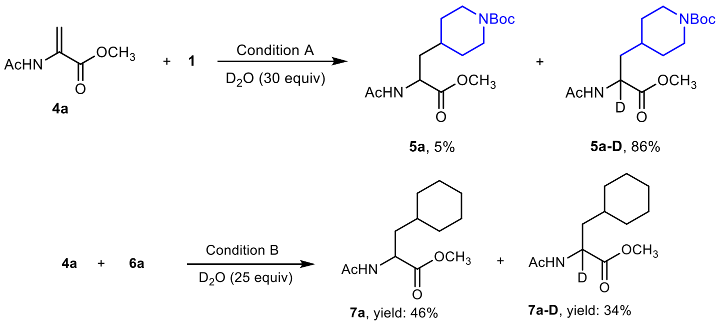

Supplementary Figure 4. H/D labeling experiments.

To an oven-dried 10 mL quartz test tube with a stirring bar was added **4a** (0.1 mmol), pyridinium salt (**1**, 0.25 mmol, 2.5 equiv), Et_3N (0.2 mmol, 2 equiv), and K_2CO_3 (0.1 mmol 1 equiv). Then, air was withdrawn and backfilled with Ar (three times). CH_3CN (1.5 mL) and D_2O (3 mmol, 30 equiv) were added. The mixture was transferred to a violet LED photoreactor (24-W, 410-420 nm), where it was irradiated for 12 h. Then, the reaction was quenched with water (5 mL), extracted with ethyl acetate, washed with brine, dried over anhydrous sodium sulfate, concentrated in vacuo, and purified by column chromatography (hexane/acetone) to afford the product (yield: 91%), the yield of deuterated product **5a-D** was 86% by ^{1}H NMR analysis.

To an oven-dried 10 mL quartz test tube with a stirring bar was added **4a** (0.1 mmol), **6a** (0.2 mmol, 2.0 equiv), PPh_3 (0.24 mmol, 2.4 equiv), and $KOCH_3$ (0.30 mmol, 3.0 equiv). Air was withdrawn and backfilled with Ar (three times). Then, acetone (1.5 mL) and D_2O (2.5 mmol, 25 equiv) were added. The mixture was transferred to a violet LED photoreactor (24-W, 420-430 nm), where it was irradiated for 12 h. Then, the reaction was quenched with water (5 mL), extracted with

ethyl acetate, washed with brine, dried over anhydrous sodium sulfate, concentrated in vacuo, and purified by chromatography on silica gel to afford the product (yield: 80%), the yield of deuterated product **7a-D** was 34% by ^{1}H NMR analysis..

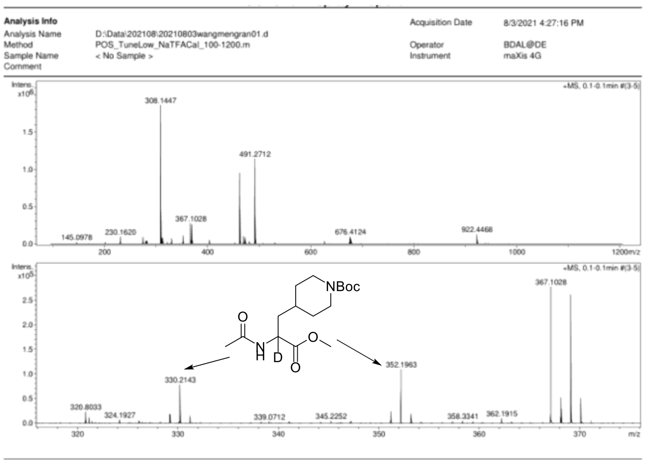

Supplementary Figure 5. The HRMS of **5a-D.**

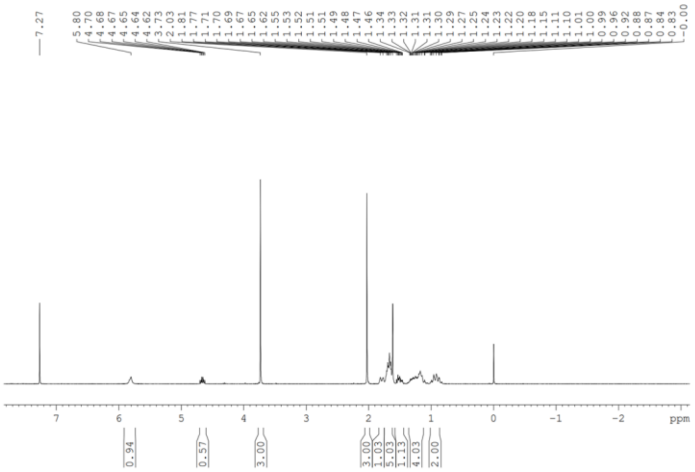

Supplementary Figure 6. The ^{1}H NMR of **7a**.

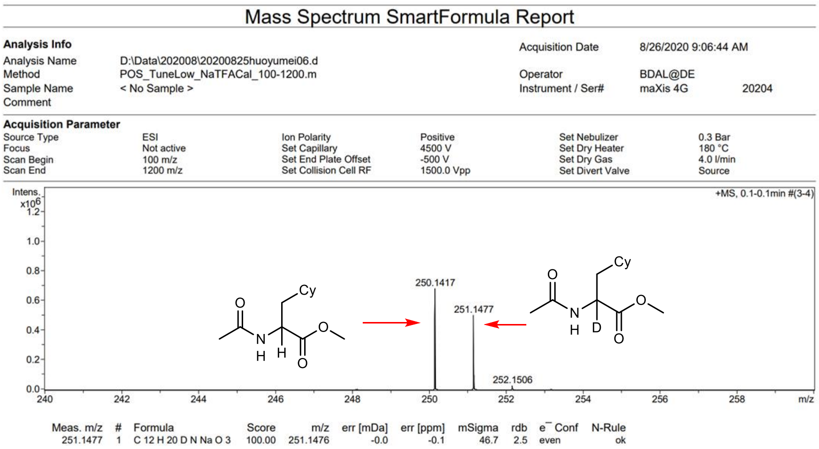

Supplementary Figure 7. The HRMS of **7a** and **7a-D.**

5.3 Byproduct analysis

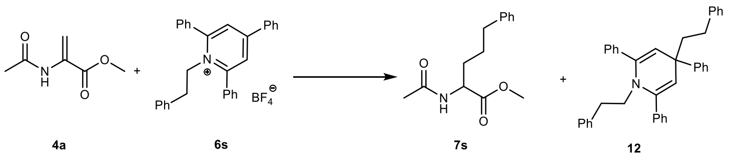

condition	7s (%)	12 (%)
A	0	18
B	0	24

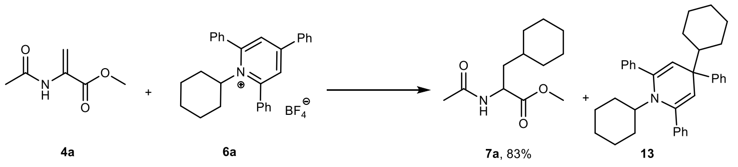

condition	7a (%)	13 (%)
A	83	5
B	80	7

NOTE: When the primary alkyl substrate **6s** (Katritzky salts derived from phenylethylamine) was used in the reaction, significant amounts of byproduct **12** (18% and 24% yields based on **6s**) was obtained. In contrast, when the secondary alkyl substrate **6a** (Katritzky salts derived from cyclohexylamine) was used, only 5% and 7% (based on **6a**) byproduct **13** was obtained, and the desired product **7a** was isolated in 83% and 80% yields.

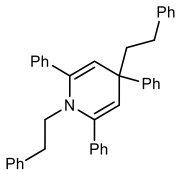

Spectroscopic data were consistent with those previously reported[12].

^{1}H NMR (300 MHz, $CDCl_3$): δ 7.59-7.54(m, 6H), 7.44-7.27 (m, 9H), 7.23-7.10 (m, 5H), 6.99-6.97 (m, 3H), 6.48-6.45 (m, 2H), 5.17 (s, 2H), 3.26-3.20 (m, 2H), 2.69-2.64 (m, 2H), 2.36-2.28 (m, 2H), 2.17-2.11 (m, 2H).

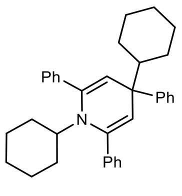

Spectroscopic data were consistent with those previously reported[13].

^{1}H NMR (300 MHz, $CDCl_3$): δ 7.61 (dd, J = 7.9, 1.5 Hz, 4H), 7.45-7.27 (m, 10H), 7.14 (t, J = 7.2 Hz, 1H), 5.27 (s, 2H), 2.87-2.72 (m, 1H), 1.67-1.40 (m, 6H), 1.27 (br, 4H), 1.21-0.91 (m, 6H), 0.87 (dd, J = 13.8, 6.7 Hz, 1H), 0.78-0.60 (m, 4H).

5.4 Analysis of UV-Vis absorption spectra

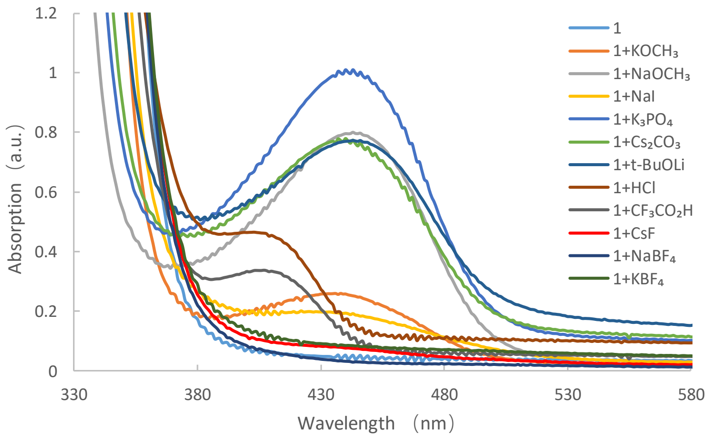

Supplementary Figure 8. The UV-Vis absorption spectrum of **1** and various ionic compounds

In Supplementary Figure 8, the UV-Vis absorption spectrum of **1** (5 x 10^{-3}M), [**1** + $KOCH_3$] (5 x 10^{-3}M, **1**:$KOCH_3$ = 1:1.5), [**1** + $NaOCH_3$] (5 x 10^{-3}M, 1:$NaOCH_3$ = 1:1.5), [**1** + NaI] (5 x 10^{-3}M, **1**:NaI = 1:1.5), [**1** + K_3PO_4] (5 x 10^{-3}M, **1**:K_3PO_4 = 1:1.5), [**1** + Cs_2CO_3] (5 x 10^{-3}M, **1**:Cs_2CO_3 = 1:1.5), [**1** + ^{t}BuOLi] (5 x 10^{-3}M, **1**:^{t}BuOLi = 1:1.5), [**1** + HCl] (5 x 10^{-3}M, **1**: HCl = 1:1.5), [**1** + CF_3CO_2H] (5 x 10^{-3}M, **1**:CF_3CO_2H = 1:1.5), [**1** + CsF] (5 x 10^{-3}M, **1**: CsF = 1:1.5), [**1** + $NaBF_4$] (5 x 10^{-3}M, **1**: $NaBF_4$ = 1:1.5), [**1** + KBF_4] (5 x 10^{-3}M, **1**: KBF_4 = 1:1.5) in acetone were provided, respectively. We found that some ionic compounds, such as Na_2CO_3, NaI, $KOCH_3$, HCl, CF_3CO_2H, and etc., could dramatically increase the absorption of **1** in visible light (400-480 nm) region. The combination [**1** + KBF_4] showed slight absorption in visible light region, whereas [**1** + $NaBF_4$] was not effective. The results indicated that BF_4^- counter anion in Katritzky salt was not crucial for the transformation.

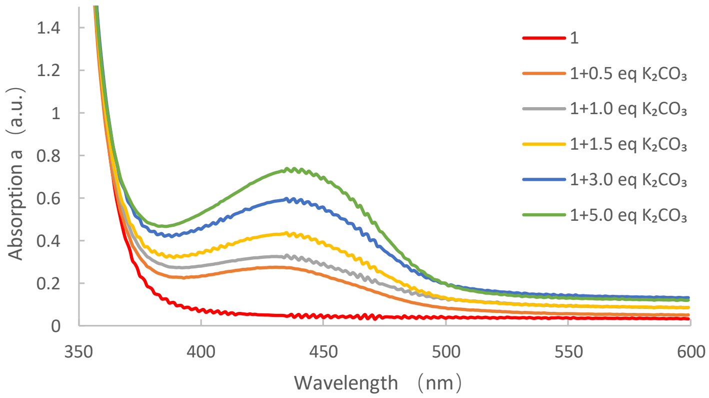

Supplementary Figure 9. The UV-Vis absorption spectrum of **1** and various concentration of K_2CO_3 in acetone.

In Supplementary Figure 9, the UV-Vis absorption spectrum of **1** (5 x 10^{-3}M), [**1** + K_2CO_3] (5 x 10^{-3}M, **1**: K_2CO_3 = 1:0.5), [**1**+ K_2CO_3] (5 x 10^{-3}M, **1**: K_2CO_3 = 1:1), [1 + K_2CO_3] (5 x 10^{-3}M, **1**: K_2CO_3 = 1:1.5), [**1** + K_2CO_3] (5 x 10^{-3}M, **1**: K_2CO_3 = 1:3), [**1**+ K_2CO_3] (5 x 10^{-3}M, **1**: K_2CO_3 = 1:5)

in acetone were provided, respectively. The absorption of **1** was less than 380 nm, whereas the mixture of **1** and K_2CO_3 exhibit new absorptions between 400-480 nm. The loading amount of K_2CO_3 was directly proportional to the light absorption intensity, indicating the key role of ionic additives for increasing the visible light absorption.

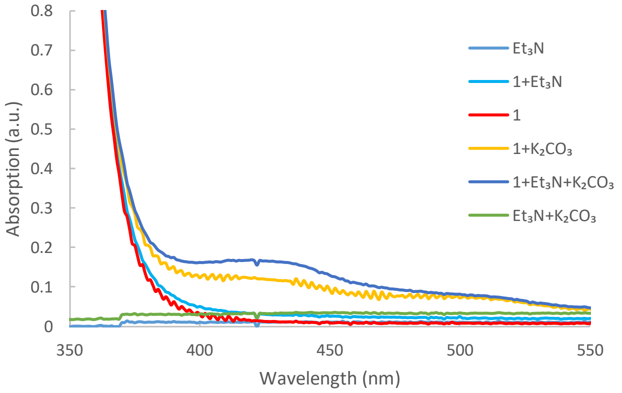

Supplementary Figure 10. The UV-Vis spectrum of the components in Condition A.

In Supplementary Figure 10, the UV-Vis absorption spectrum of Et_3N (5 x 10^{-3}M), [**1** + Et_3N] (5 x 10^{-3}M, **1**: Et_3N = 1:1), [**1**] (5 x 10^{-3}M), [**1** + K_2CO_3] (5 x 10^{-3}M, **1**: K_2CO_3 = 1:1.5), [**1**+Et_3N+K_2CO_3] (5 x 10^{-3}M, **1**:Et_3N:K_2CO_3 = 1:1:1.5), and [Et_3N + K_2CO_3] (5 x 10^{-3}M, Et_3N:K_2CO_3 = 1:1.5) in acetone were provided, respectively. We found the [**1**+K_2CO_3] exhibit a new absorption in 400-480 nm, [**1** + Et_3N + K_2CO_3] exhibit a significantly enhanced absorption in 400-480 nm than [**1** + K_2CO_3], while [**1** + Et_3N] showed a weak red-shift in absorbance.

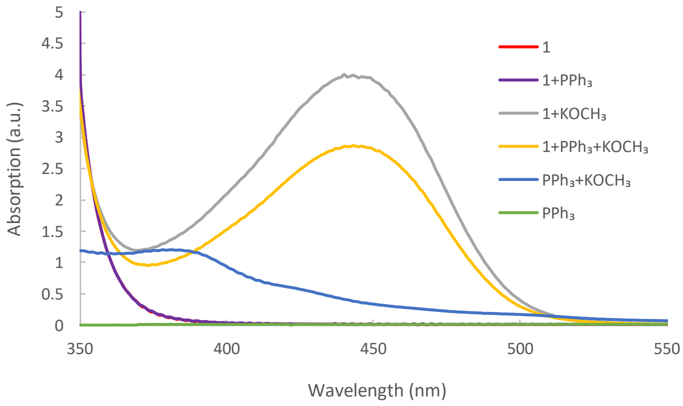

Supplementary Figure 11. The UV-Vis spectrum of the components in Condition B.

In Supplementary Figure 11, the UV-Vis absorption spectrum of **1** (5 x 10^{-3}M), [**1** + PPh_3] (5 x 10^{-3}M, **1**: PPh_3 = 1:1), [**1** + $KOCH_3$] (5 x 10^{-3}M, **1**: $KOCH_3$ = 1:1.5), [**1** + PPh_3 + $KOCH_3$] (5 x 10^{-3}M, **1**:PPh_3: $KOCH_3$ = 1:1:1.5), [PPh_3 + $KOCH_3$] (5 x 10^{-3}M, PPh_3: $KOCH_3$ = 1:1.5), and PPh_3 (5 x 10^{-3}M) in acetone were provided, respectively. We found the [**1**+$KOCH_3$] exhibit a new absorption in 400-480 nm, and [**1** + PPh_3 + $KOCH_3$] have a little weak absorption than [**1** + $KOCH_3$], while [**1** + PPh_3] have no absorption at all in 400-480 nm region.

Furthermore, in Supplementary Figure 10 and 11, a very weak red-shift in UV-Vis absorbance

was observed when Katritzky salt (**1**) was combined with Et_3N. However, there was no changes in UV-Vis absorbance when **1** was combined with PPh_3. Although we cannot completely rule out the possibility of EDA complexes in our reaction, it seems unlikely be involved in the main operating pathway.

5.5 Stern-Volmer fluorescence quenching study

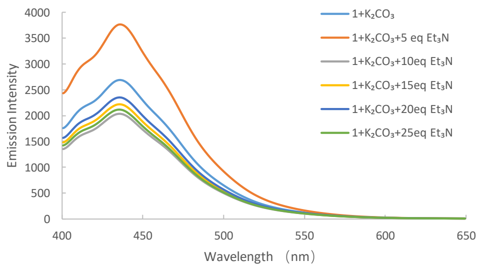

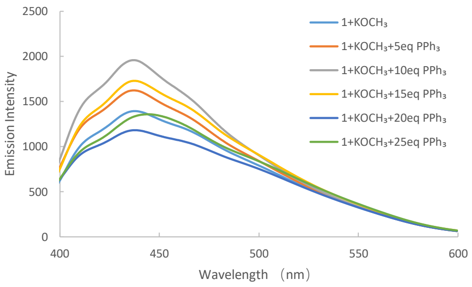

Supplementary Figure 12. Stern-Volmer plots for the emission quenching of [**1** + K_2CO_3] and [**1** + $KOCH_3$] by various concentrations of quenchers Et_3N and PPh_3.

In Supplementary Figure 12, we conducted Stern-Volmer quenching experiments based on condition A and condition B. However, the excited mixture of [**1**+K_2CO_3] (5 x 10^{-5} M, **1**: K_2CO_3 = 1:1.5) or [**1**+$KOCH_3$] (5 x 10^{-5} M, **1**: $KOCH_3$ = 1:1.5) could not been quenched by Et_3N or PPh_3, respectively. The results showed that the mixture of [**1**+K_2CO_3] or [**1**+$KOCH_3$] were not photosensitive species in the reaction.

5.6 Job's plots

A Job's plot of absorbance was performed to determine the binding stoichiometry of the mixture between the Katritzky salt **1** and ionic compounds (K_2CO_3 in condition A, $KOCH_3$ in condition B) according to the previous report[14]. We measured the absorption of acetone solutions of **1** and ionic compound having a constant total concentration of 0.005 M but different donor/acceptor ratios. The absorbance values are plotted against the molar fraction (%) of **1**. The maximum absorbance of [**1**+K_2CO_3] mixture was observed when the ratio was 1:1, whereas the maximum absorbance of [**1**+$KOCH_3$] was obtained when the ratio was 1:4. The variation of the molar ratio between **1** and ionic compound at maximum absorbance indicated that **1** and ionic compound unlikely formed an EDA complex in our reaction.

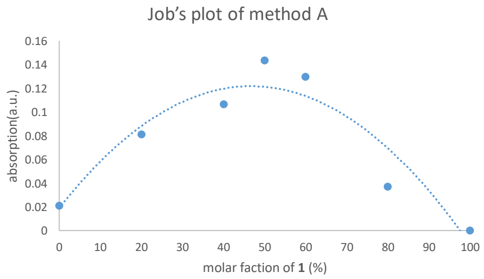

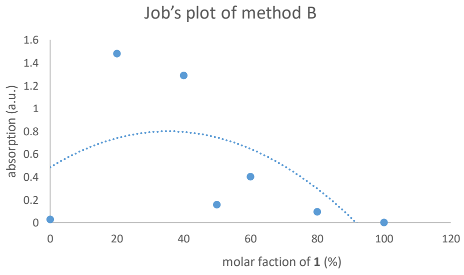

Supplementary Figure 13. Job's plot of method A and method B.

6. NMR spectra of products

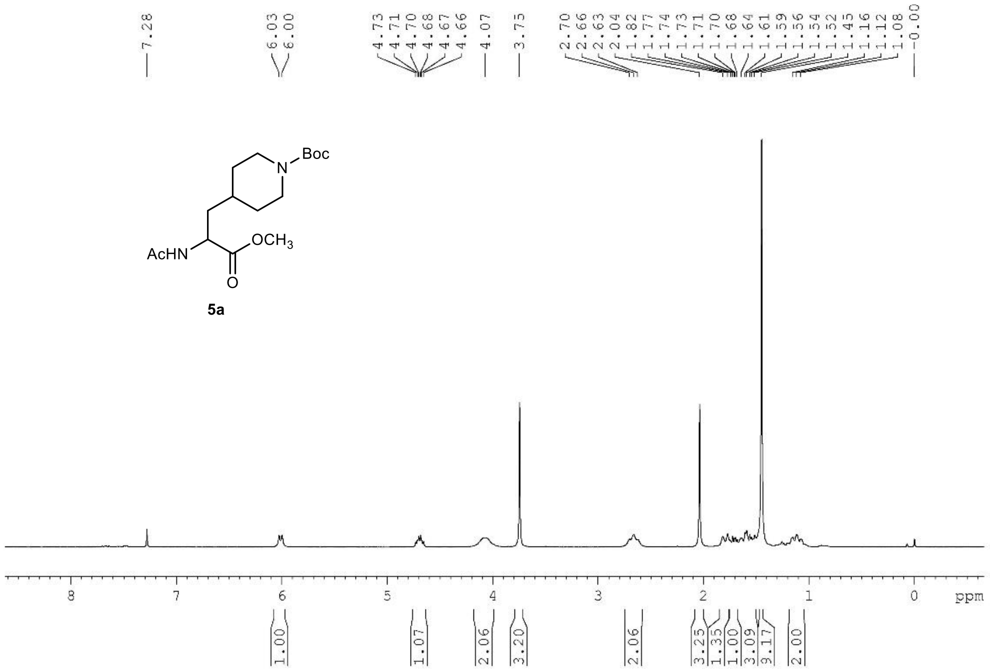

Supplementary Figure 14. ^{1}H NMR spectrum of compound **5a.**

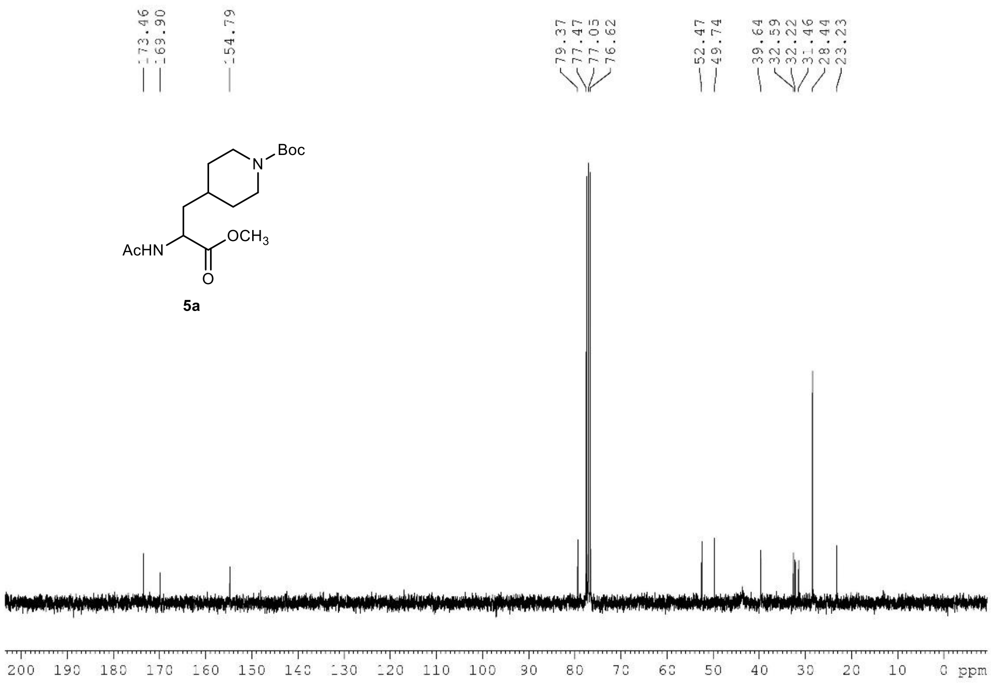

Supplementary Figure 15. ^{13}C NMR spectrum of compound **5a.**

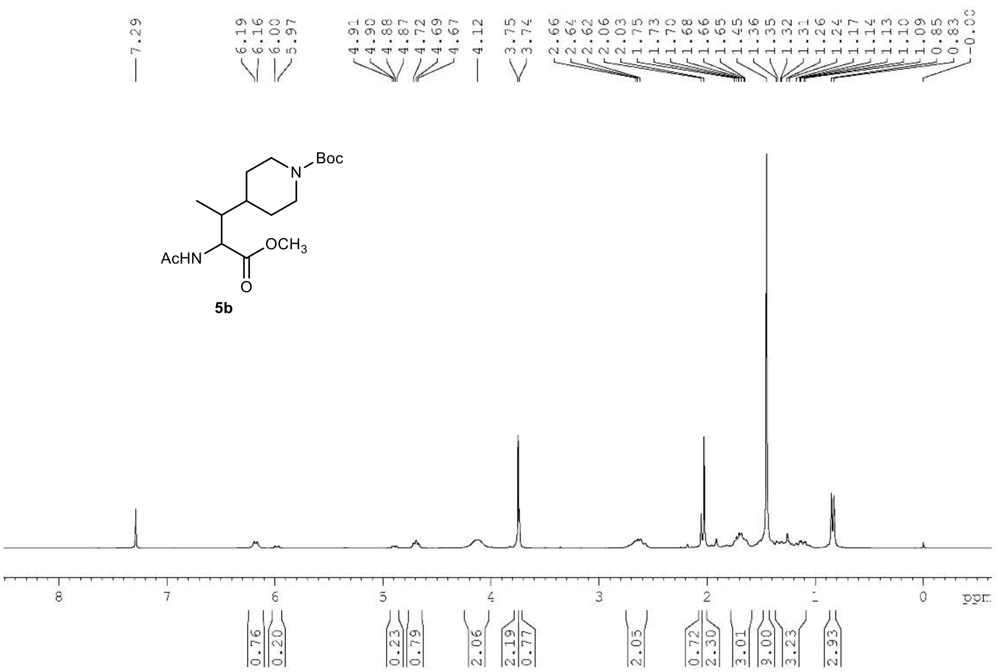

Supplementary Figure 16. ^{1}H NMR spectrum of compound **5b.**

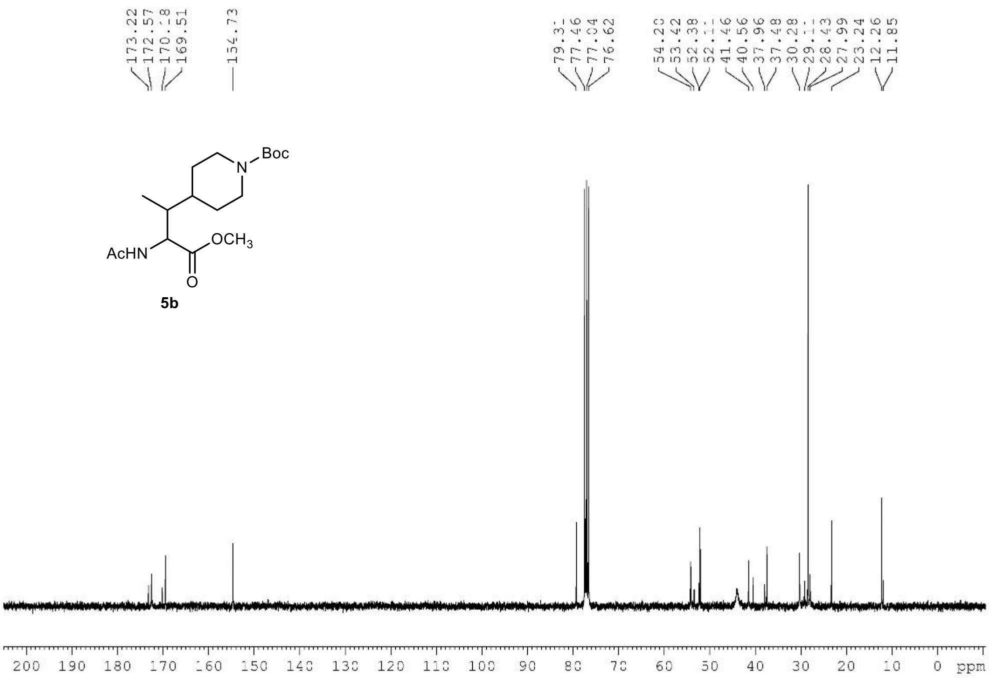

Supplementary Figure 17. ^{13}C NMR spectrum of compound **5b.**

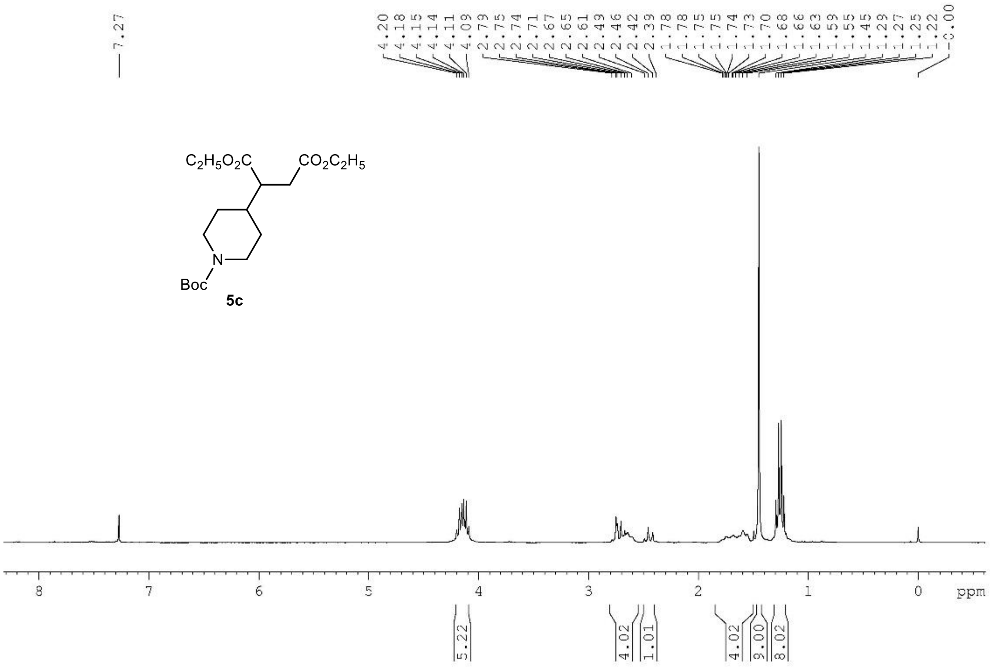

Supplementary Figure 18. ^{1}H NMR spectrum of compound **5c.**

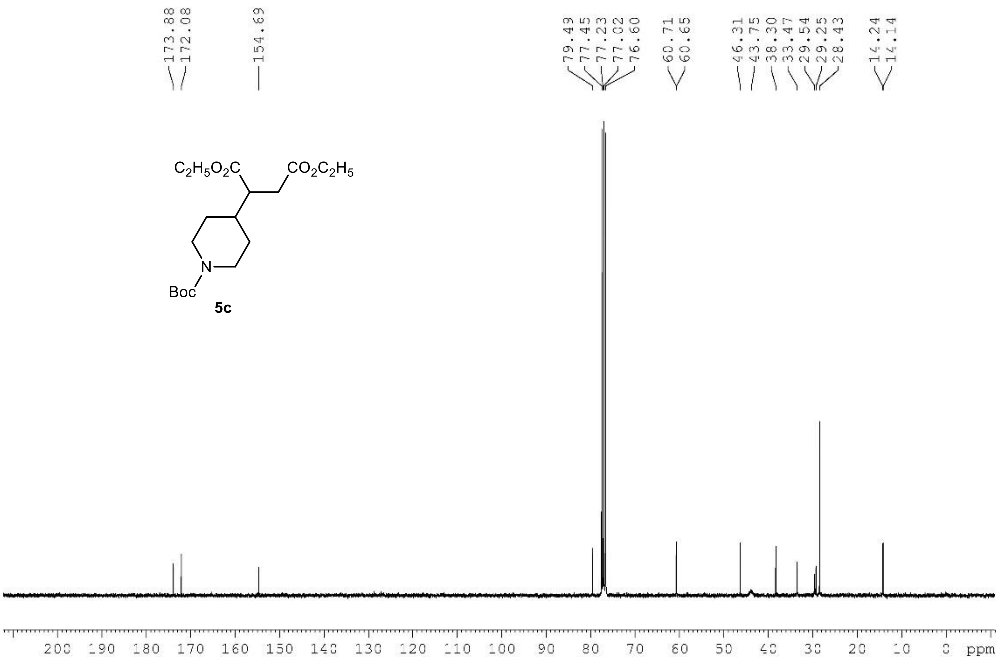

Supplementary Figure 19. ^{13}C NMR spectrum of compound **5c.**

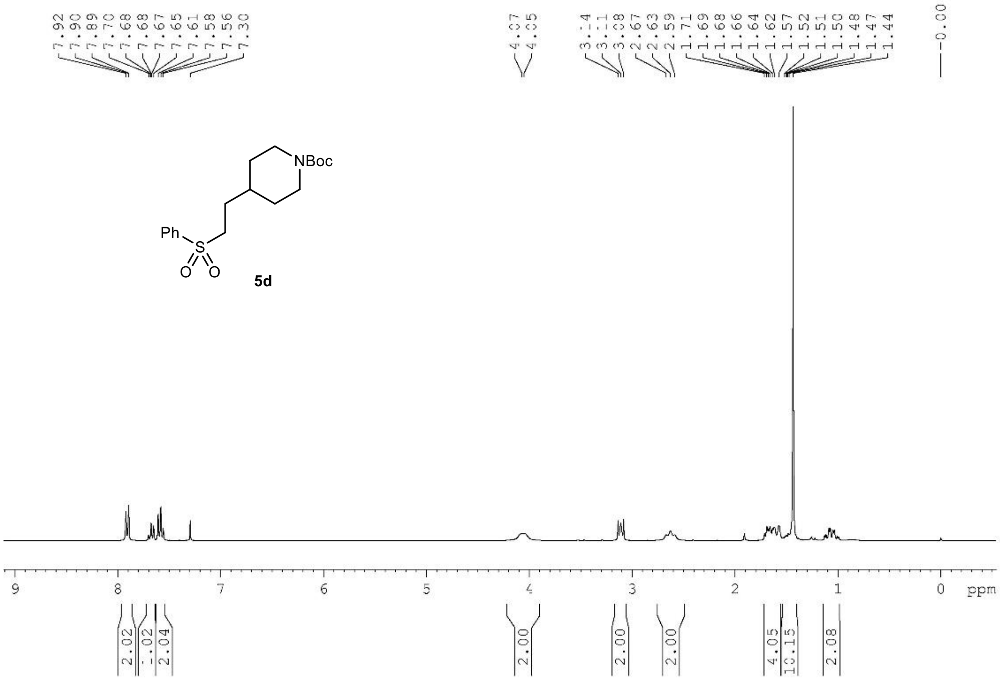

Supplementary Figure 20. ^{1}H NMR spectrum of compound **5d.**

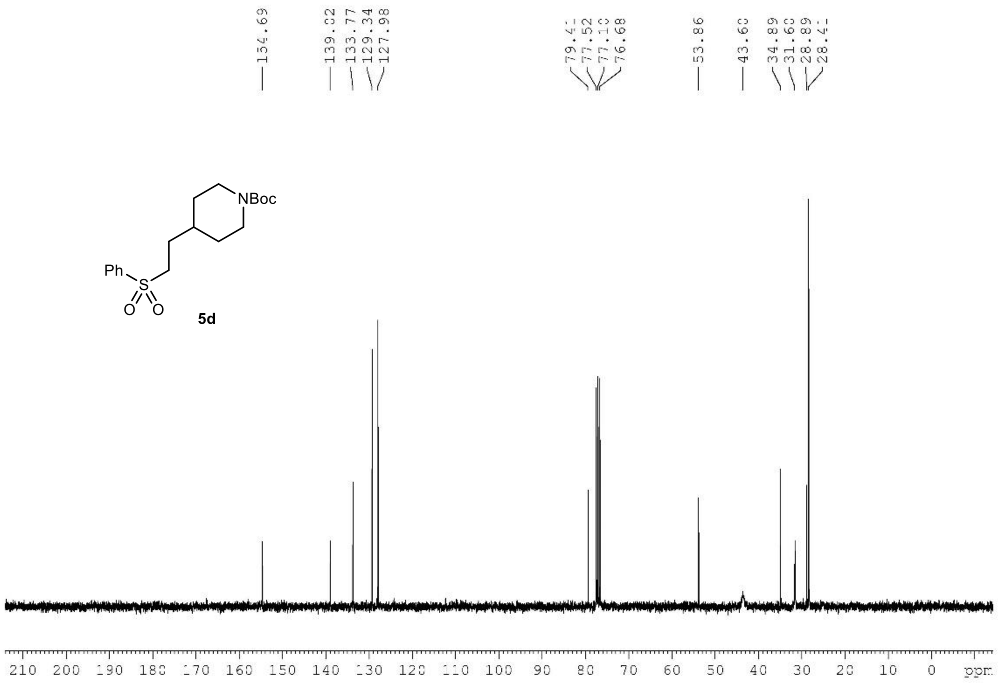

Supplementary Figure 21. ^{13}C NMR spectrum of compound **5d.**

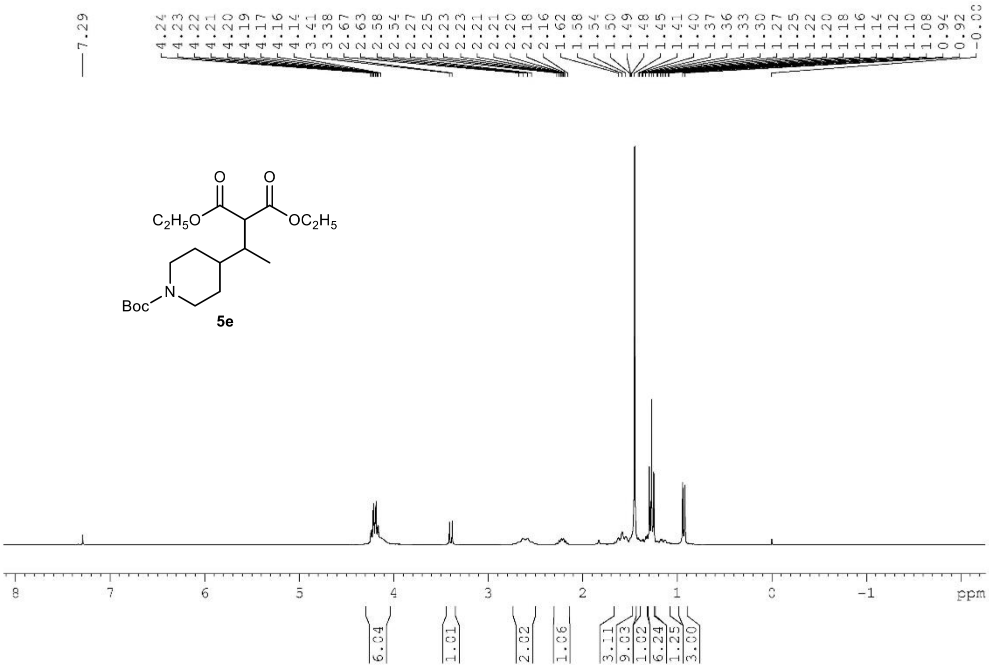

Supplementary Figure 22. ^{1}H NMR spectrum of compound **5e.**

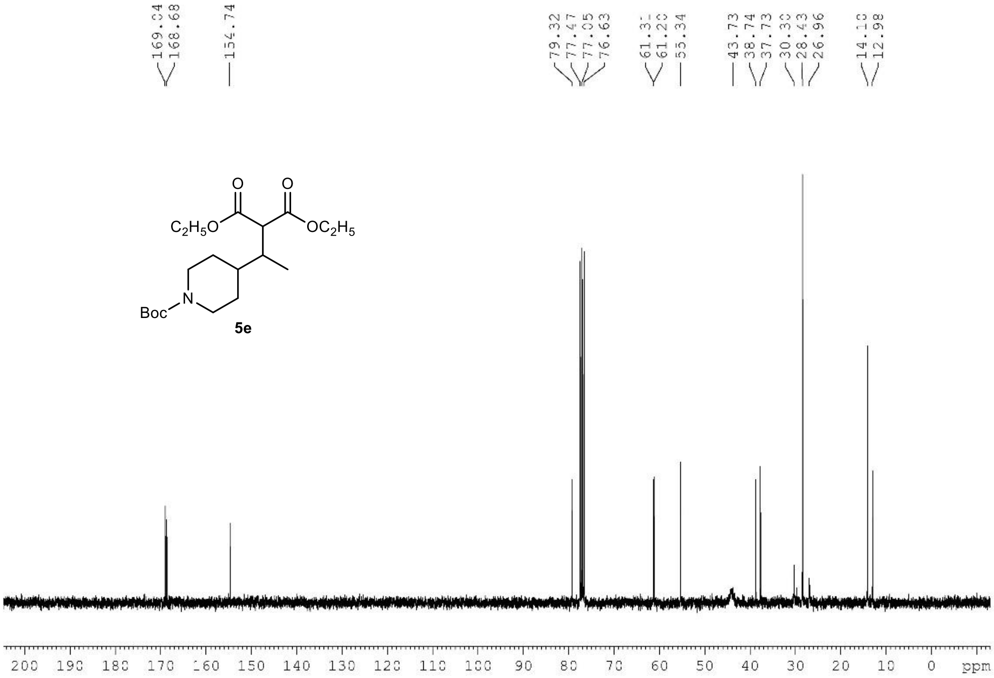

Supplementary Figure 23. ^{13}C NMR spectrum of compound **5e.**

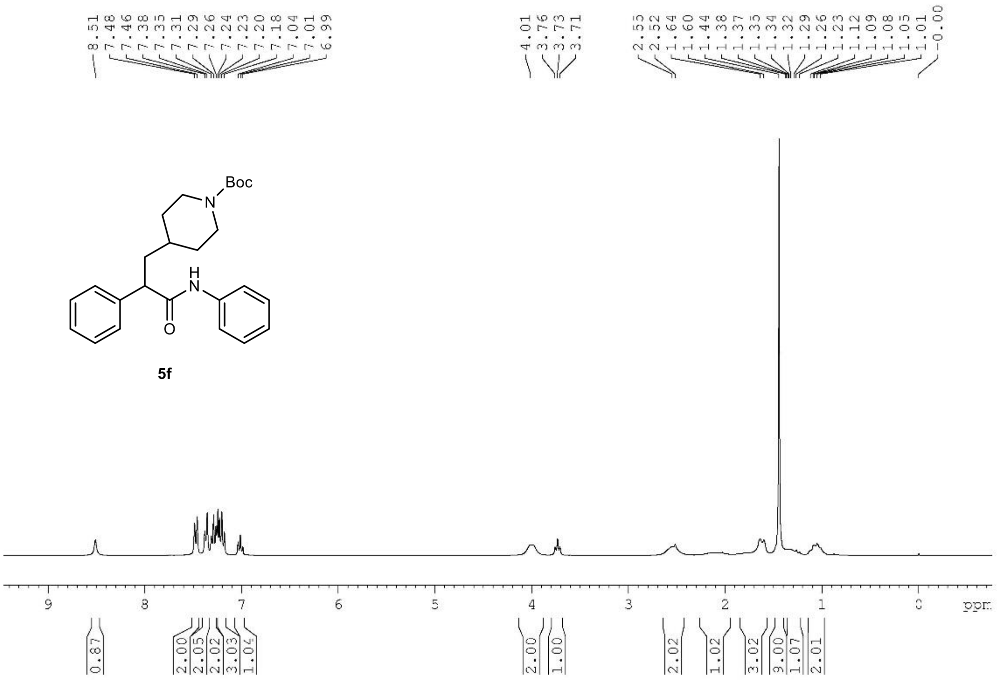

Supplementary Figure 24. ^{1}H NMR spectrum of compound **5f.**

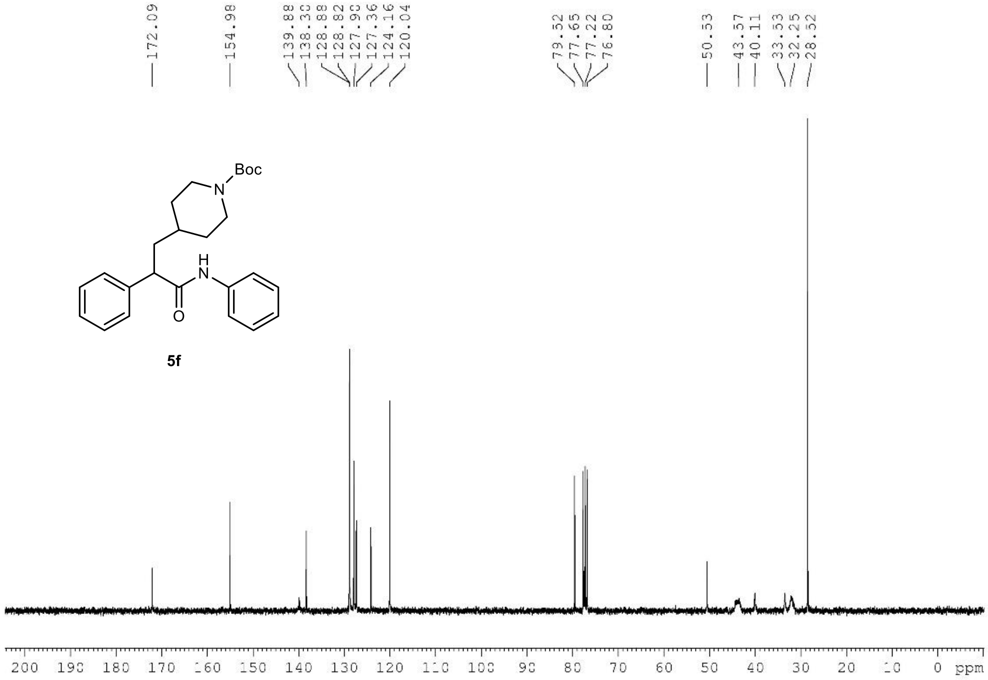

Supplementary Figure 25. ^{13}C NMR spectrum of compound **5f.**

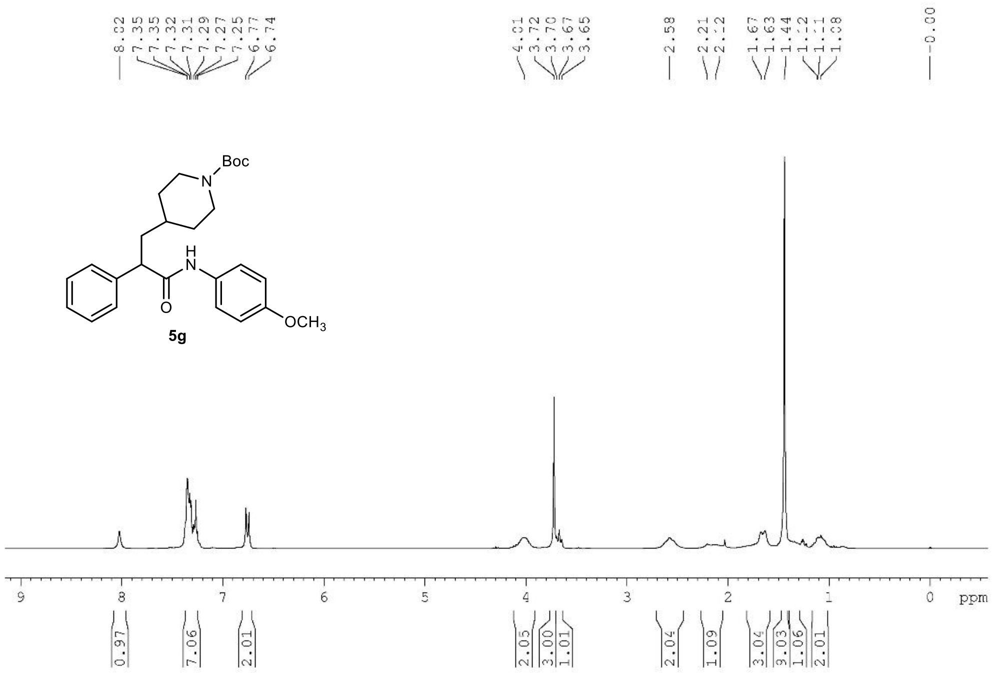

Supplementary Figure 26. ^{1}H NMR spectrum of compound **5g.**

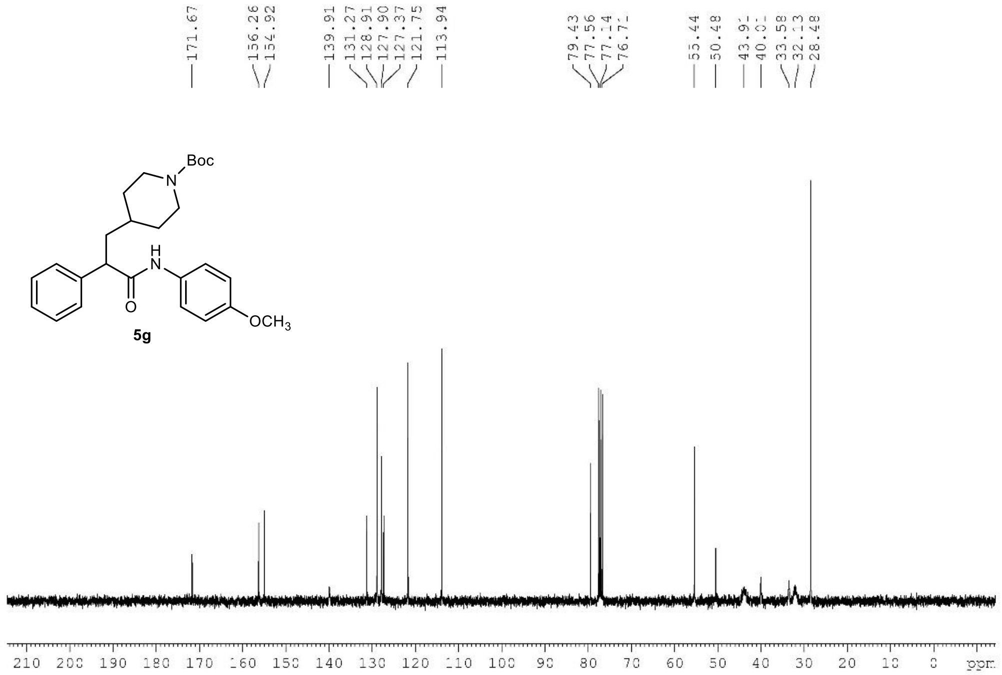

Supplementary Figure 27. ^{13}C NMR spectrum of compound **5g.**

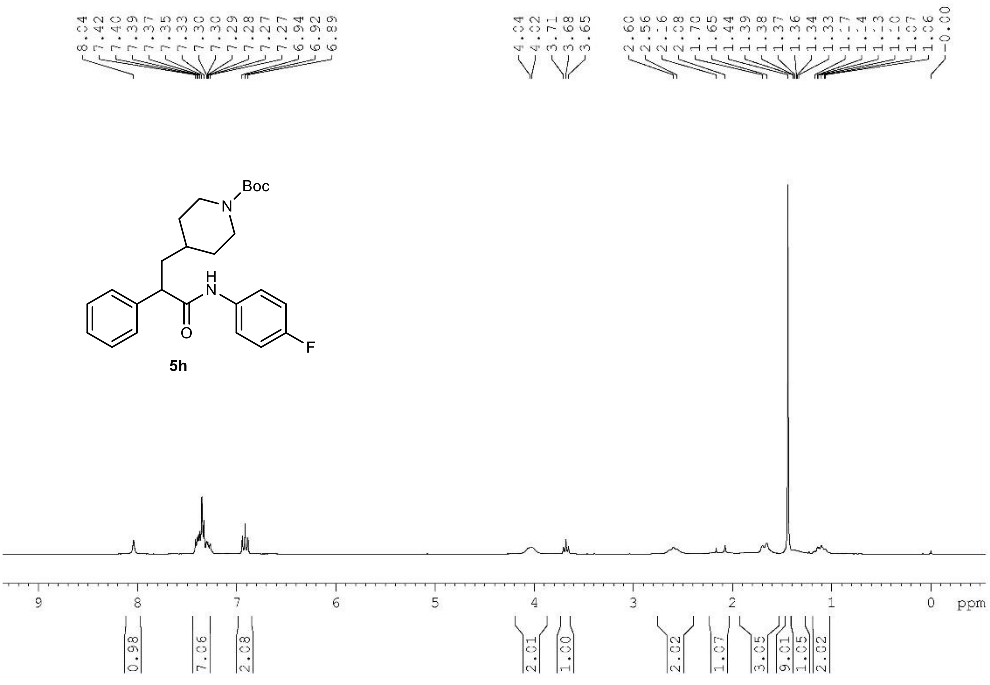

Supplementary Figure 28. ^{1}H NMR spectrum of compound **5h.**

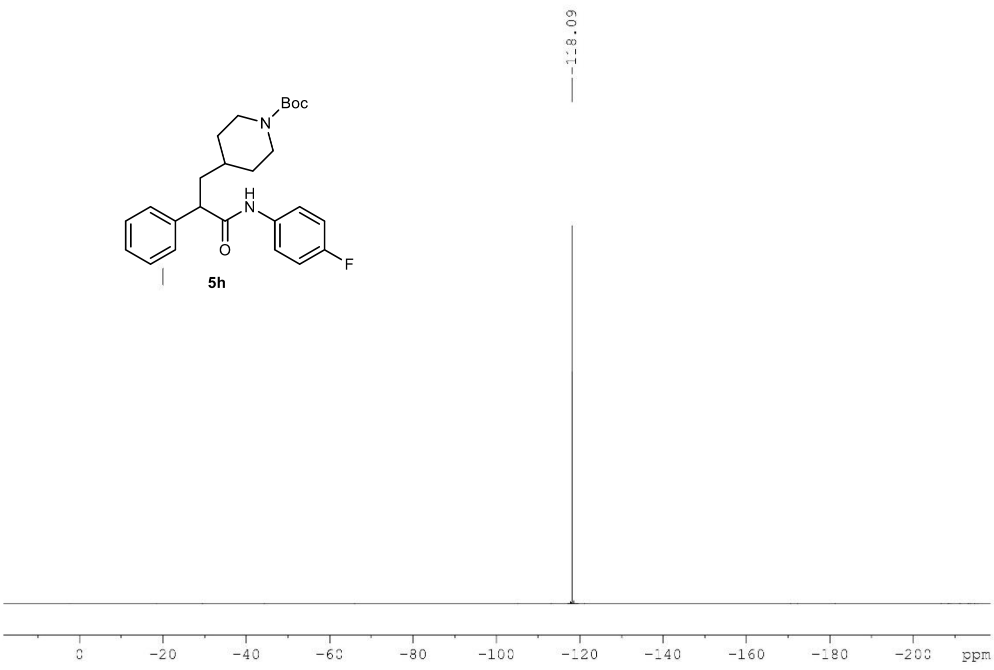

Supplementary Figure 29. ^{19}F NMR spectrum of compound **5h.**

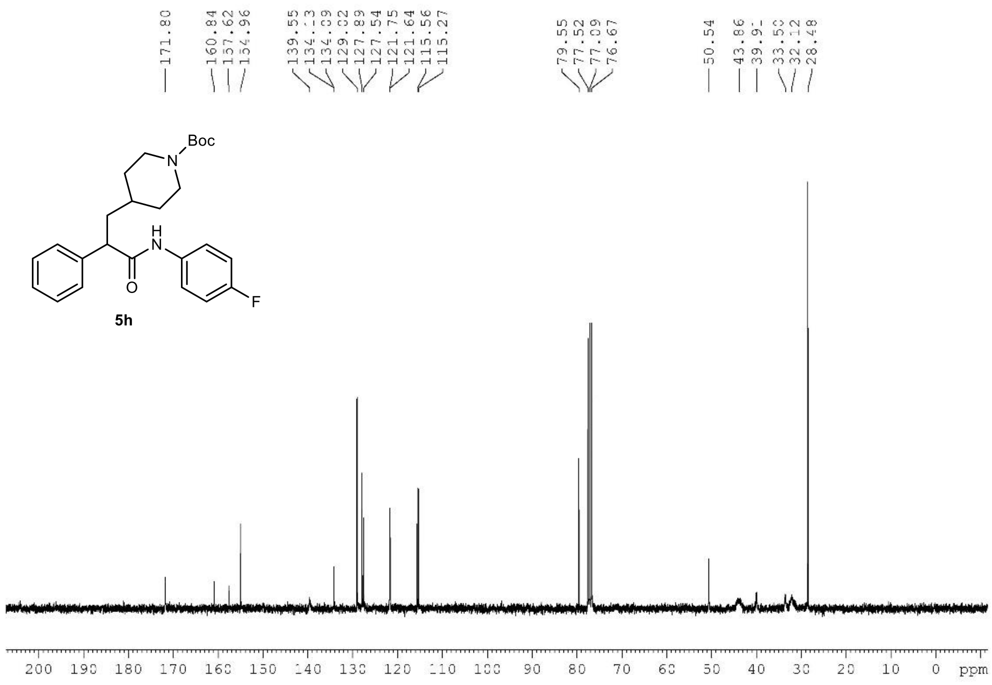

Supplementary Figure 30. ^{13}C NMR spectrum of compound **5h.**

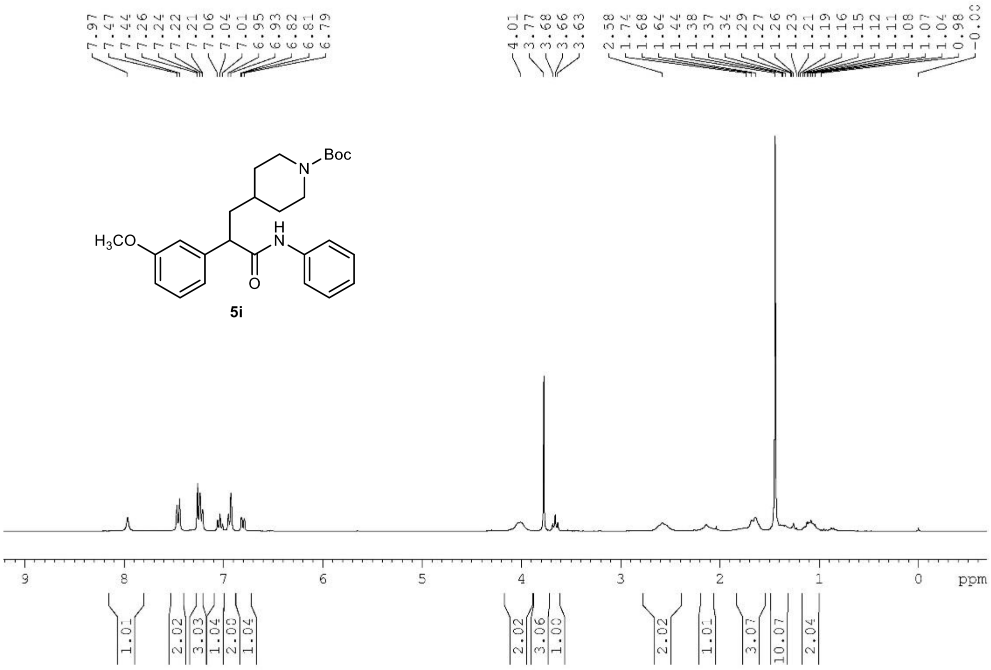

Supplementary Figure 31. ^{1}H NMR spectrum of compound **5i.**

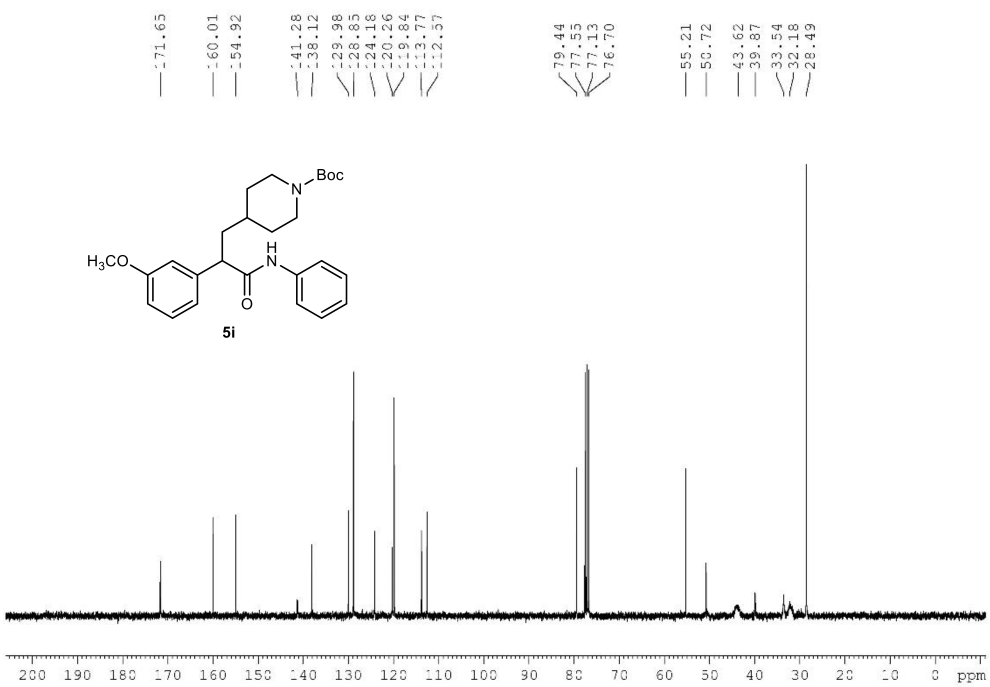

Supplementary Figure 32. ^{13}C NMR spectrum of compound **5i.**

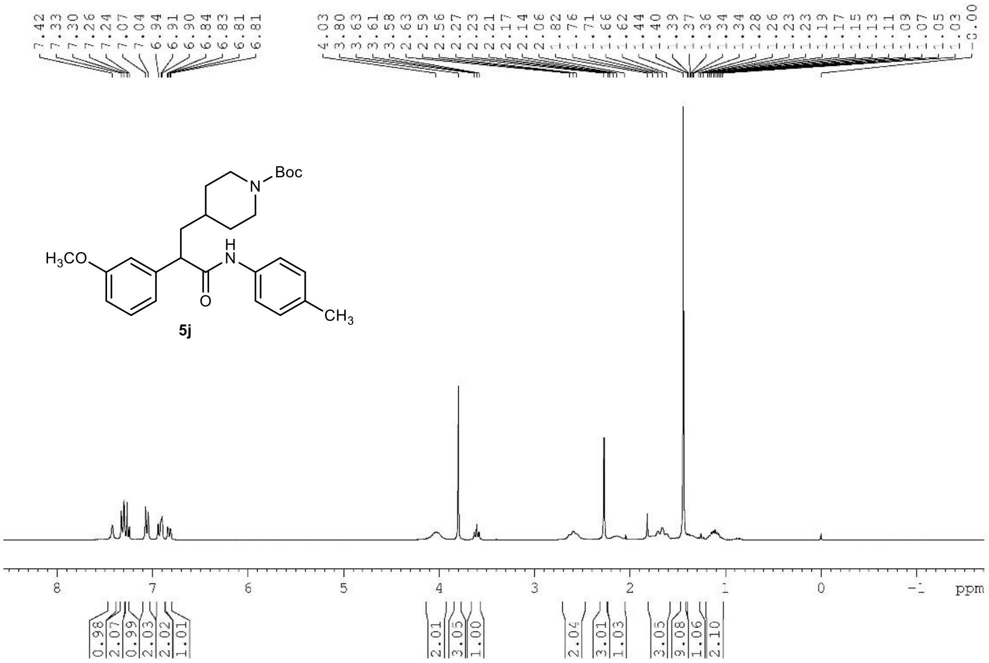

Supplementary Figure 33. ^{1}H NMR spectrum of compound **5j.**

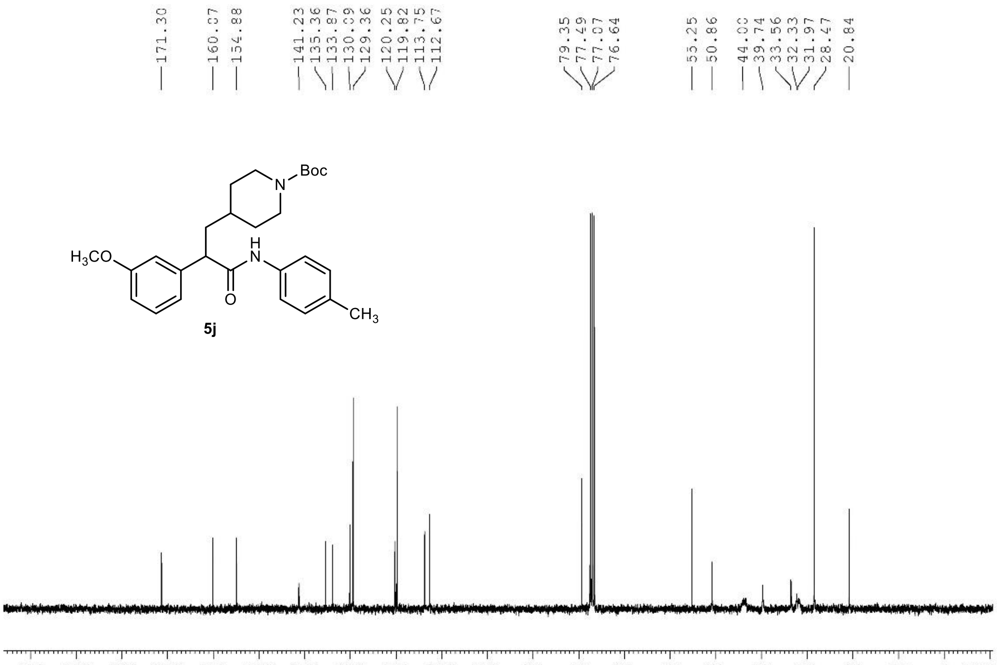

Supplementary Figure 34. ^{13}C NMR spectrum of compound **5j.**

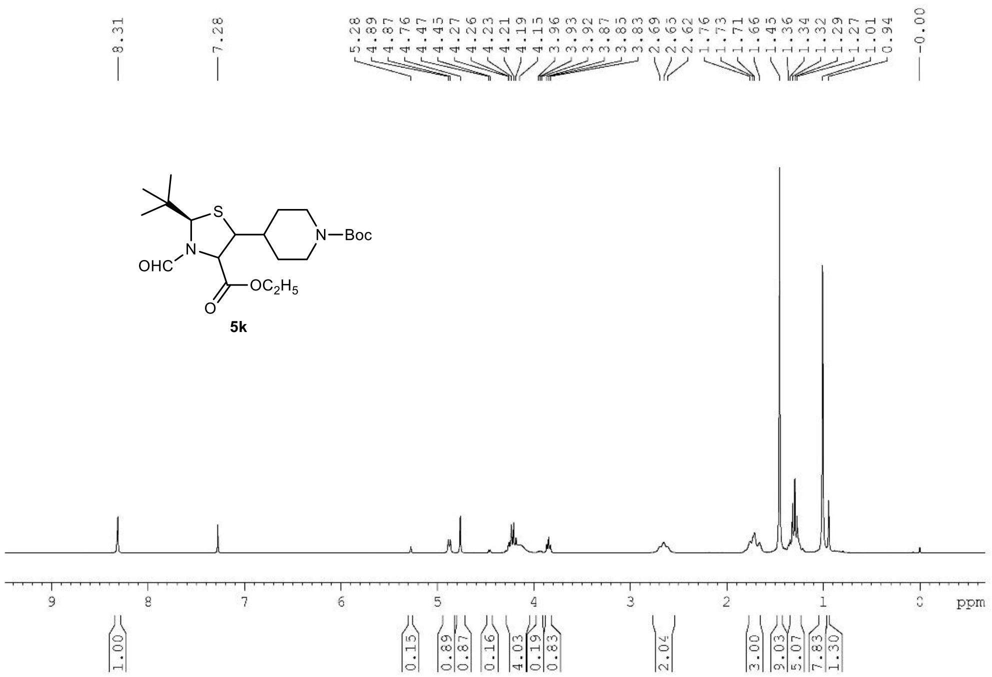

Supplementary Figure 35. ^{1}H NMR spectrum of compound **5k.**

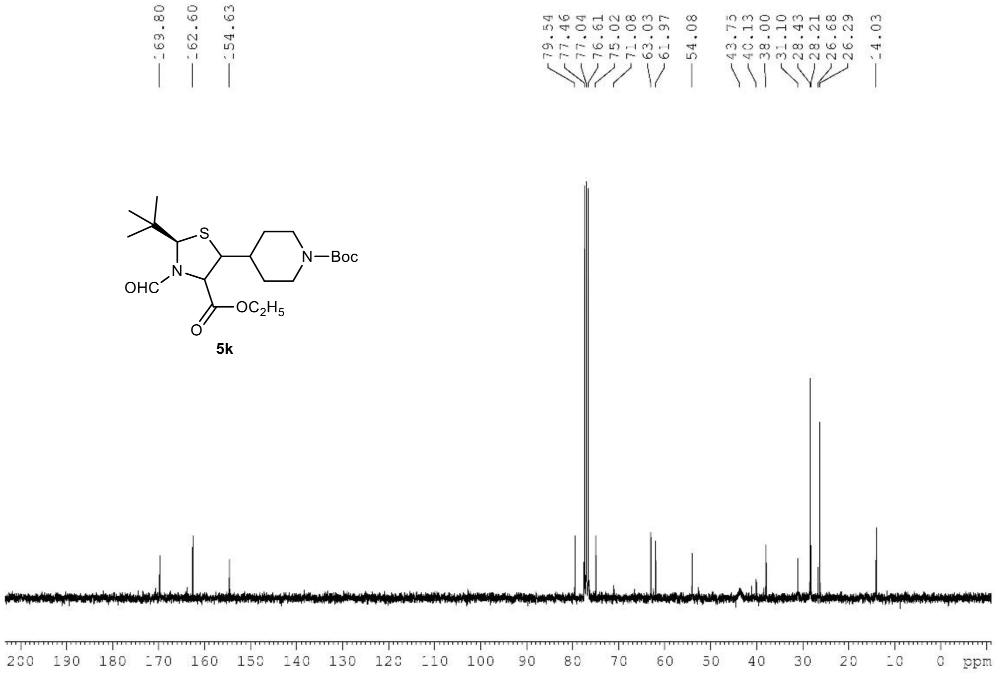

Supplementary Figure 36. ^{13}C NMR spectrum of compound **5k.**

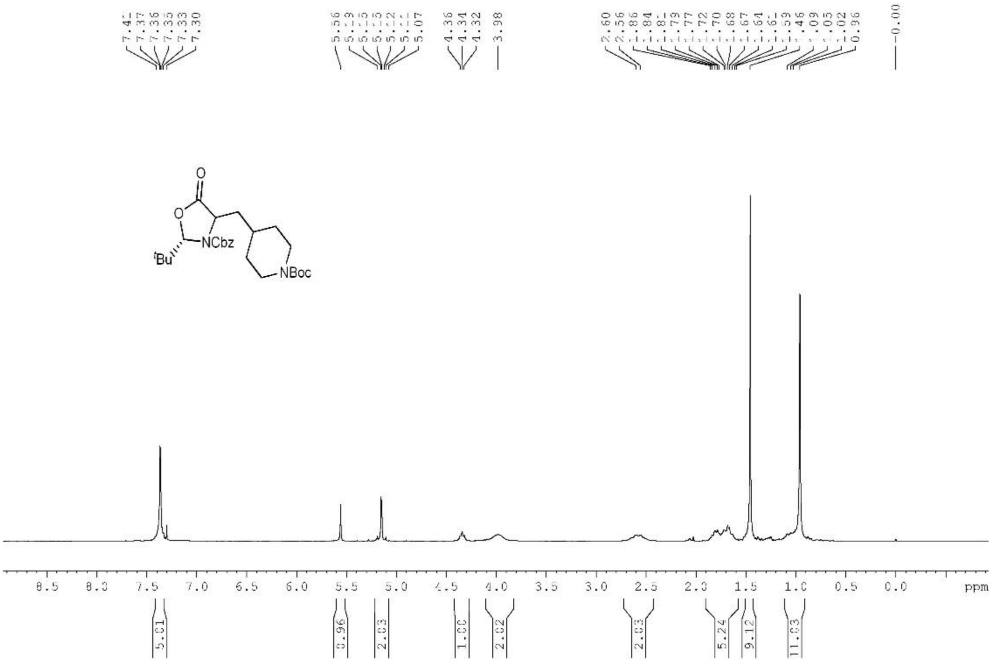

Supplementary Figure 37. ^{1}H NMR spectrum of compound **5l.**

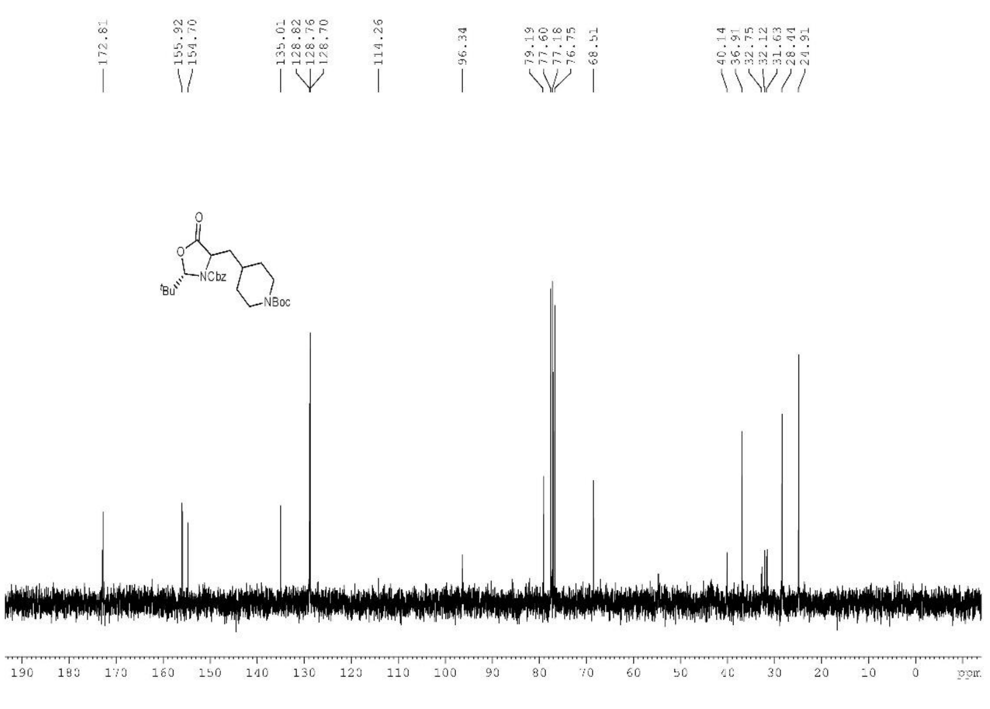

Supplementary Figure 38. ^{13}C NMR spectrum of compound **5l.**

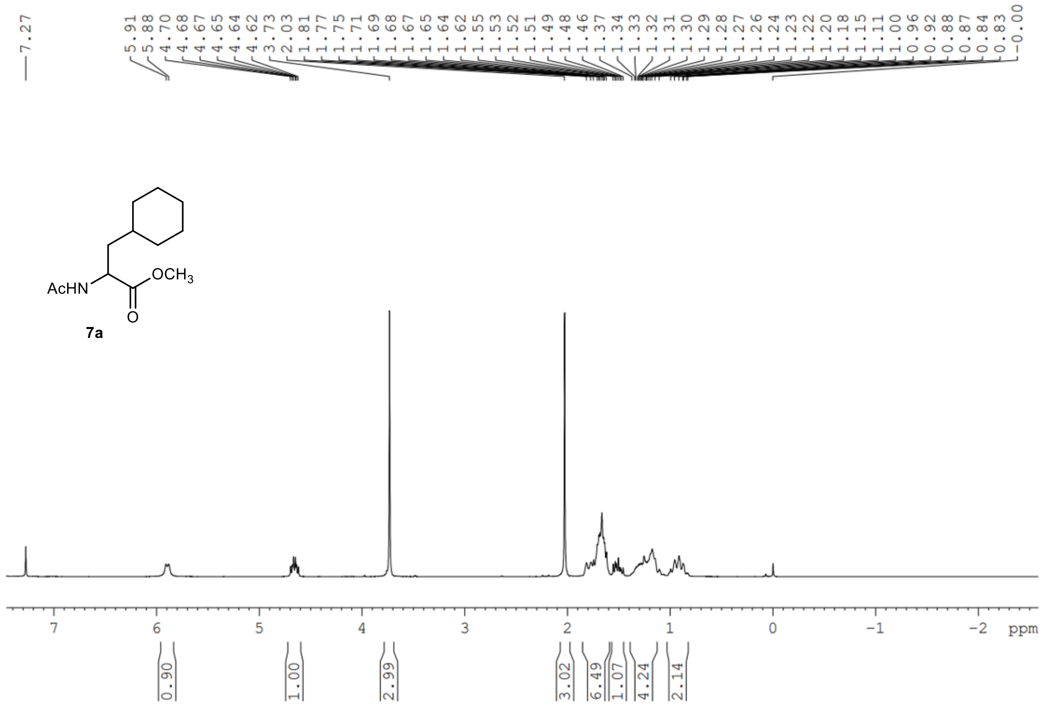

Supplementary Figure 39. ^{1}H NMR spectrum of compound **7a.**

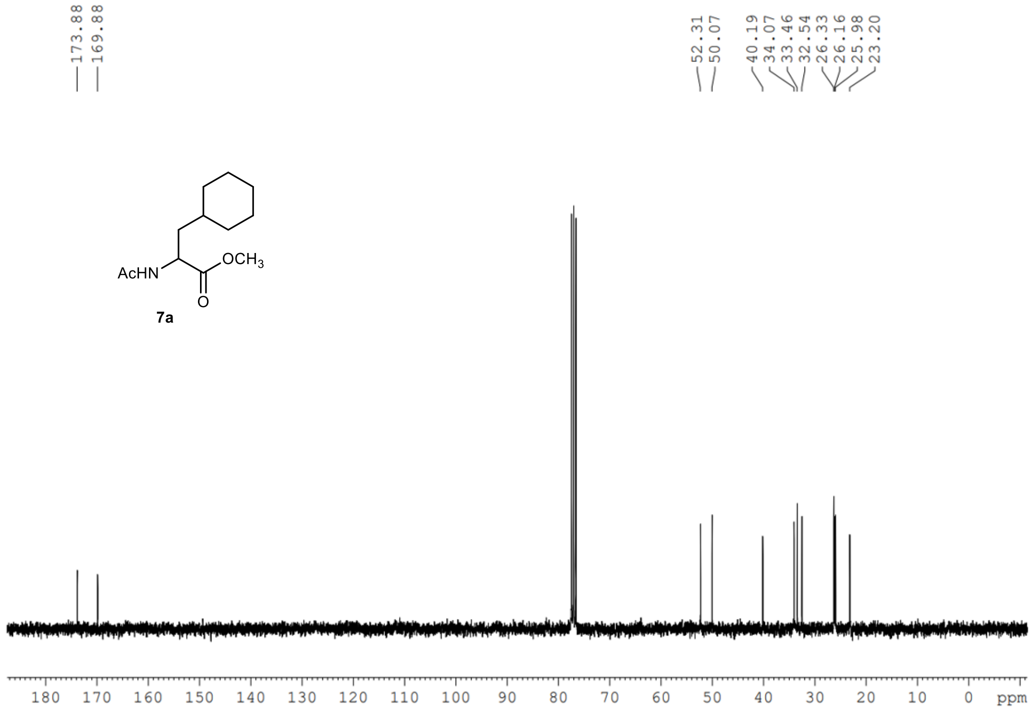

Supplementary Figure 40. ^{13}C NMR spectrum of compound **7a.**

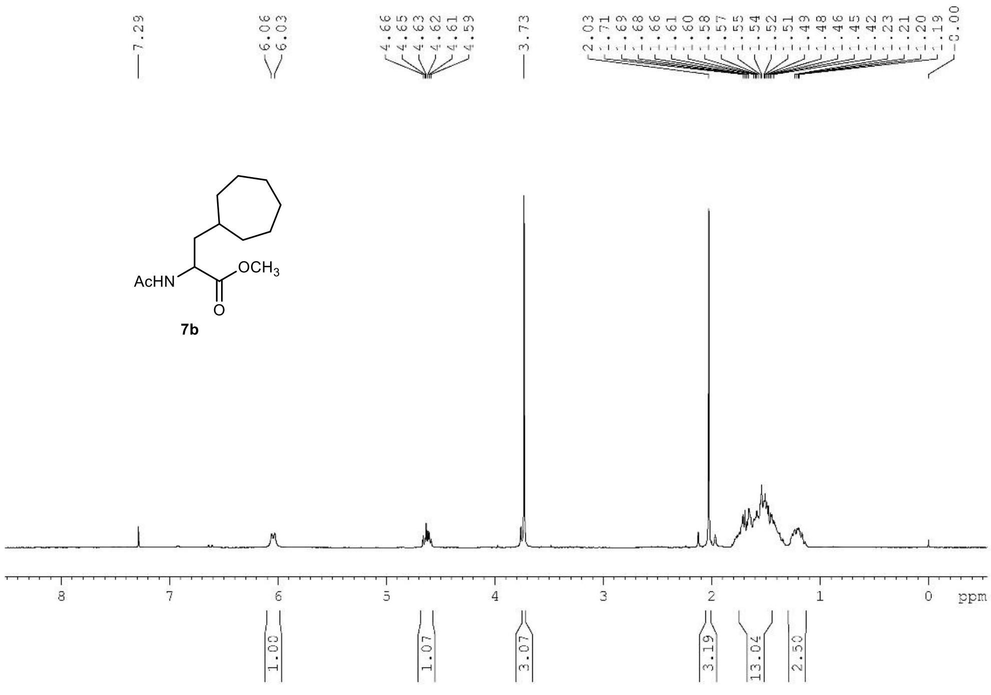

Supplementary Figure 41. ^{1}H NMR spectrum of compound **7b.**

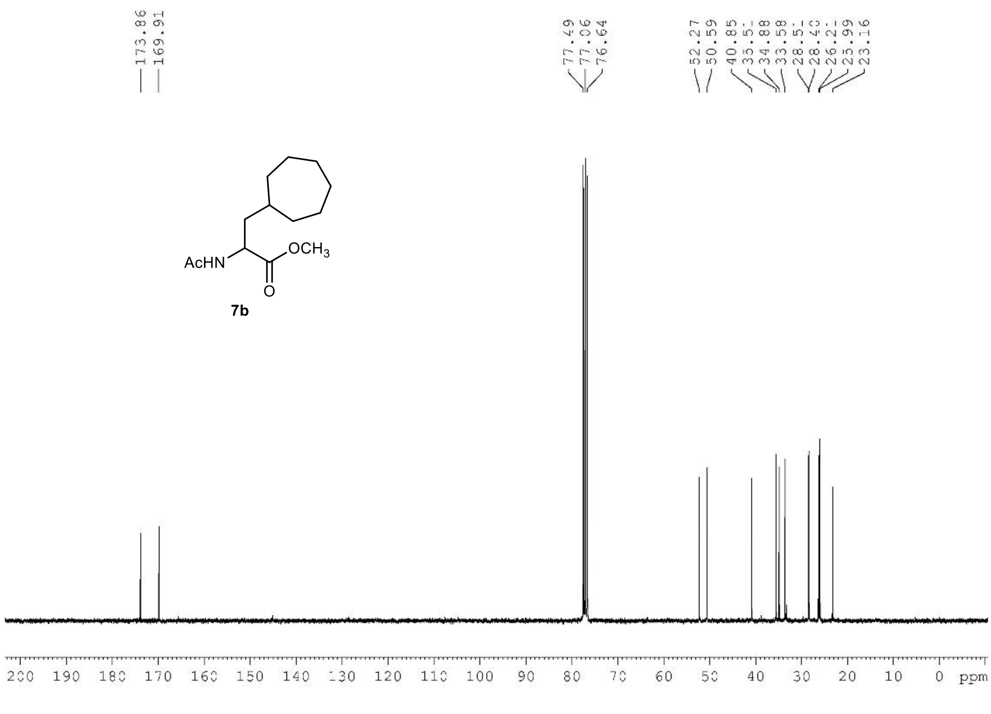

Supplementary Figure 42. ^{13}C NMR spectrum of compound **7b.**

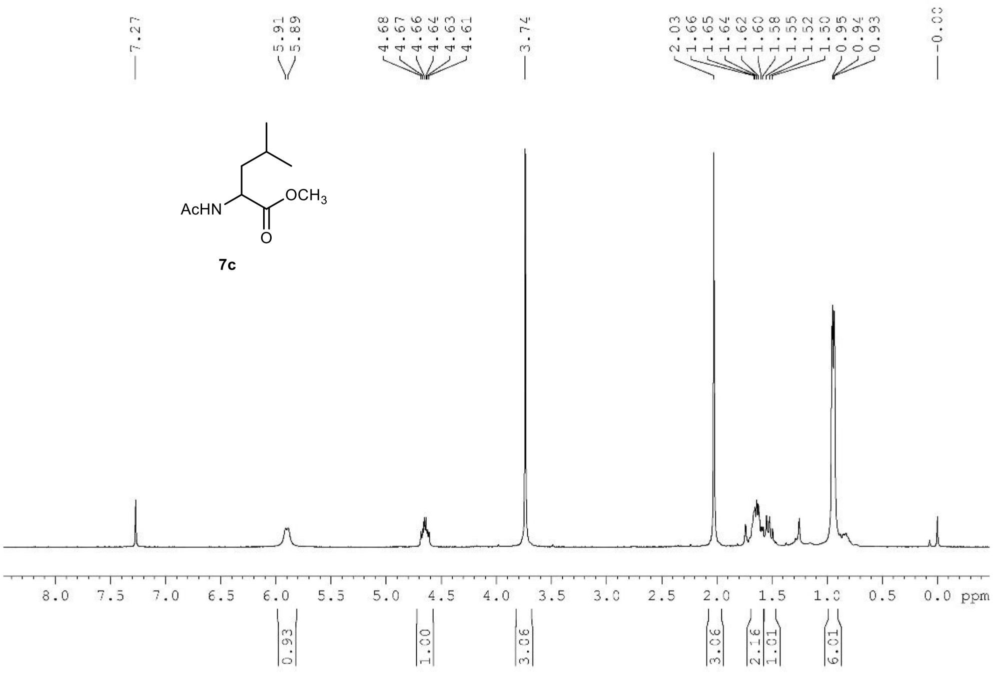

Supplementary Figure 43. ^{1}H NMR spectrum of compound **7c.**

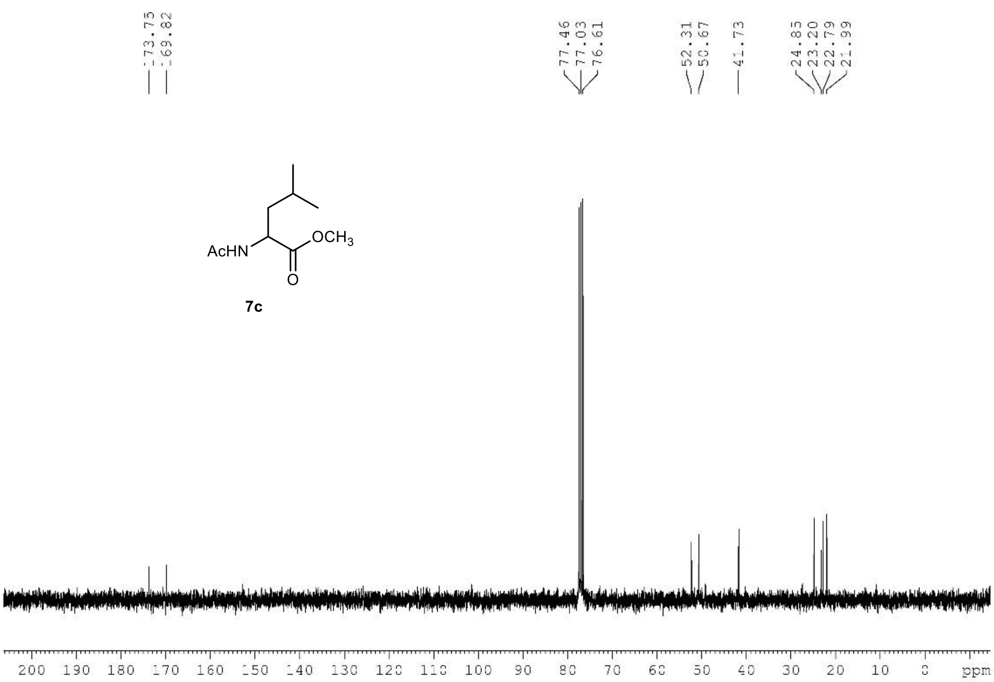

Supplementary Figure 44. ^{13}C NMR spectrum of compound **7c.**

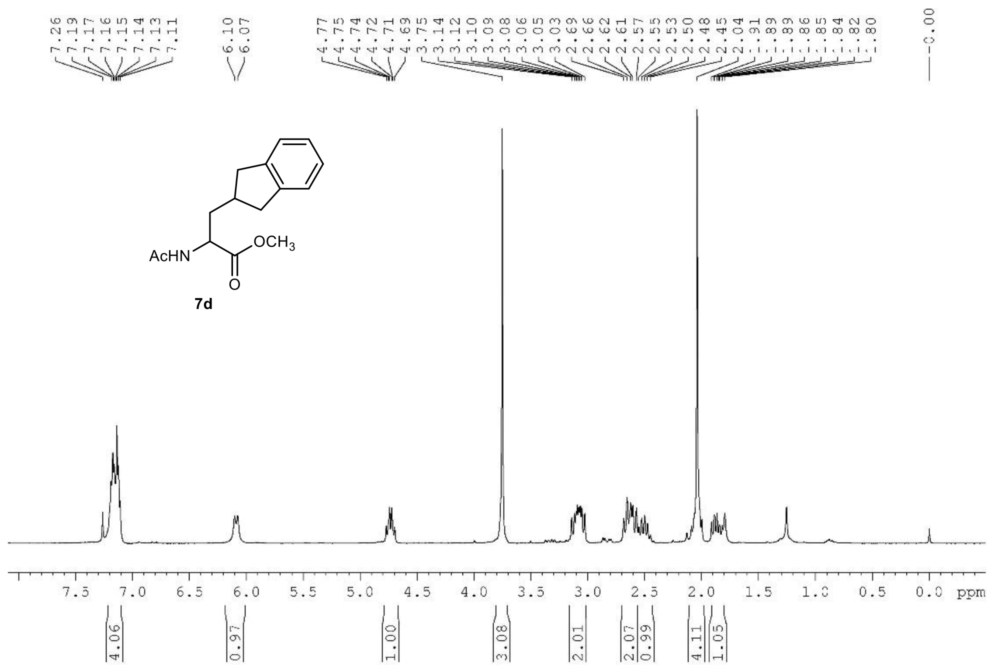

Supplementary Figure 45. ^{1}H NMR spectrum of compound **7d.**

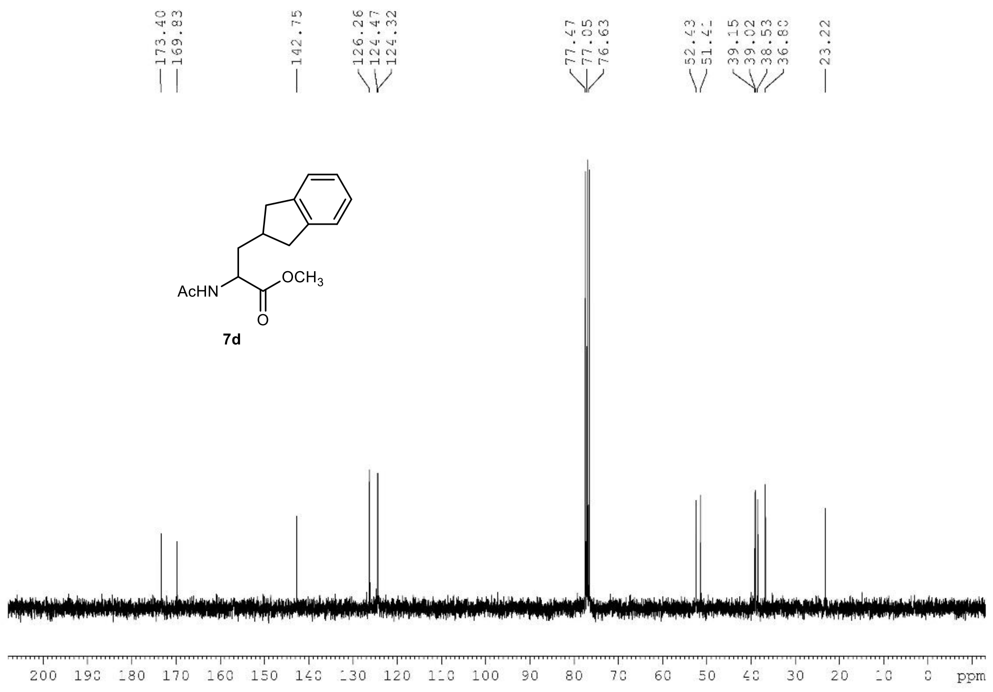

Supplementary Figure 46. ^{13}C NMR spectrum of compound **7d.**

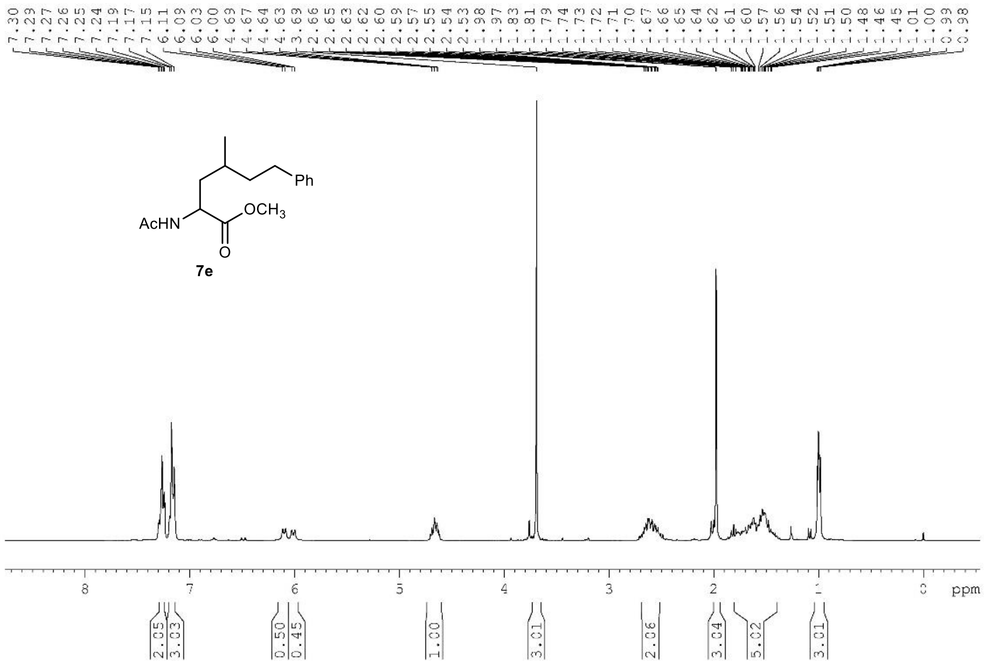

Supplementary Figure 47. ^{1}H NMR spectrum of compound **7e.**

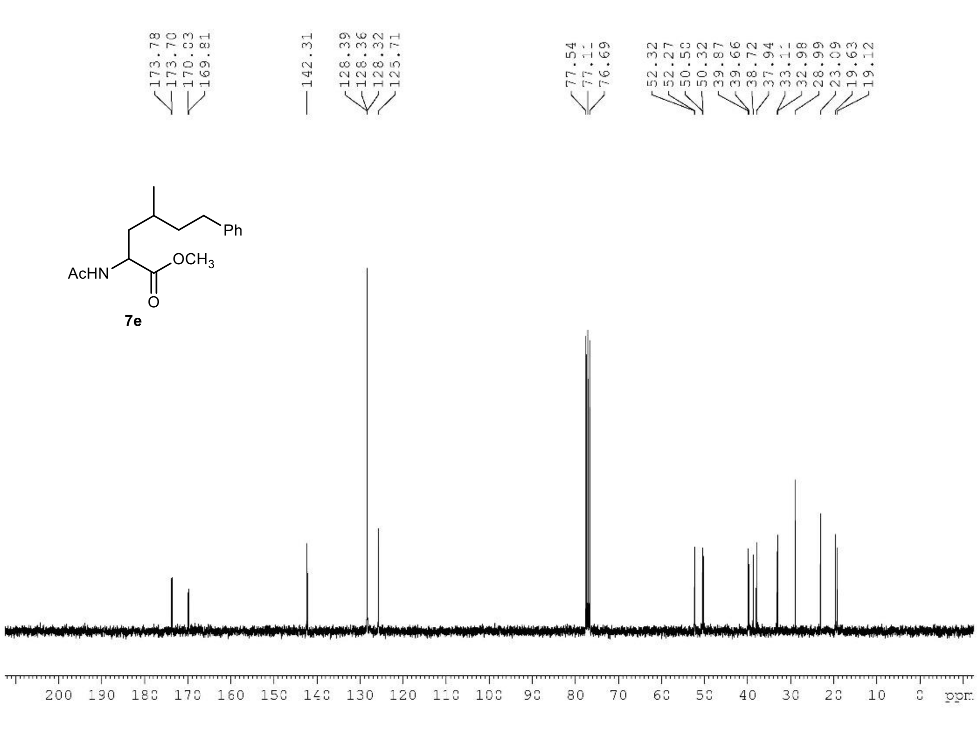

Supplementary Figure 48. ^{13}C NMR spectrum of compound **7e.**

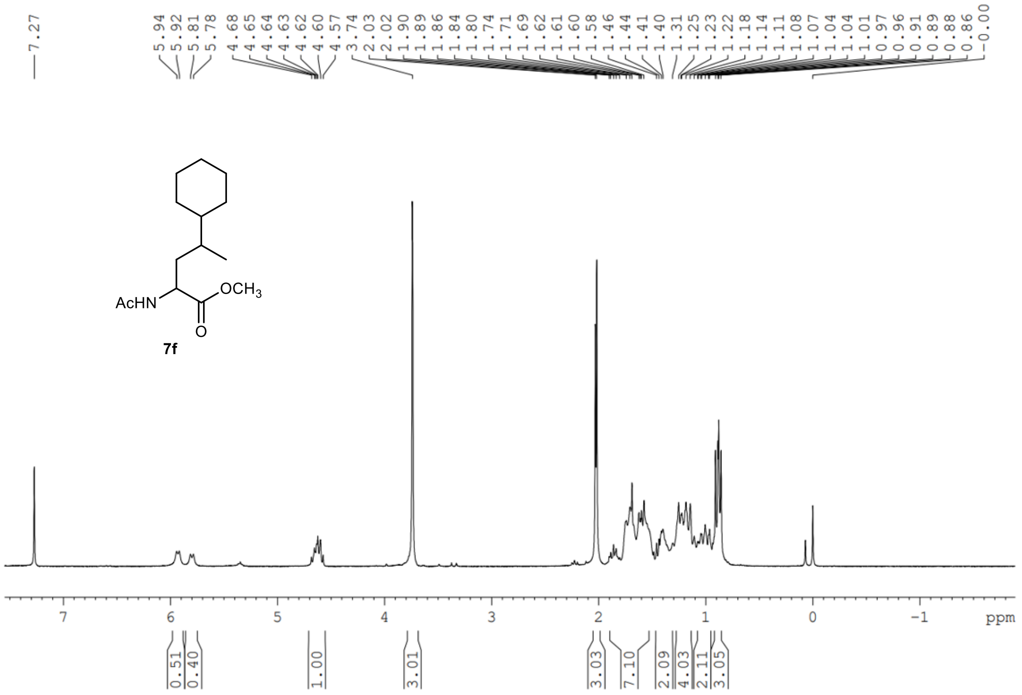

Supplementary Figure 49. ^{1}H NMR spectrum of compound **7f.**

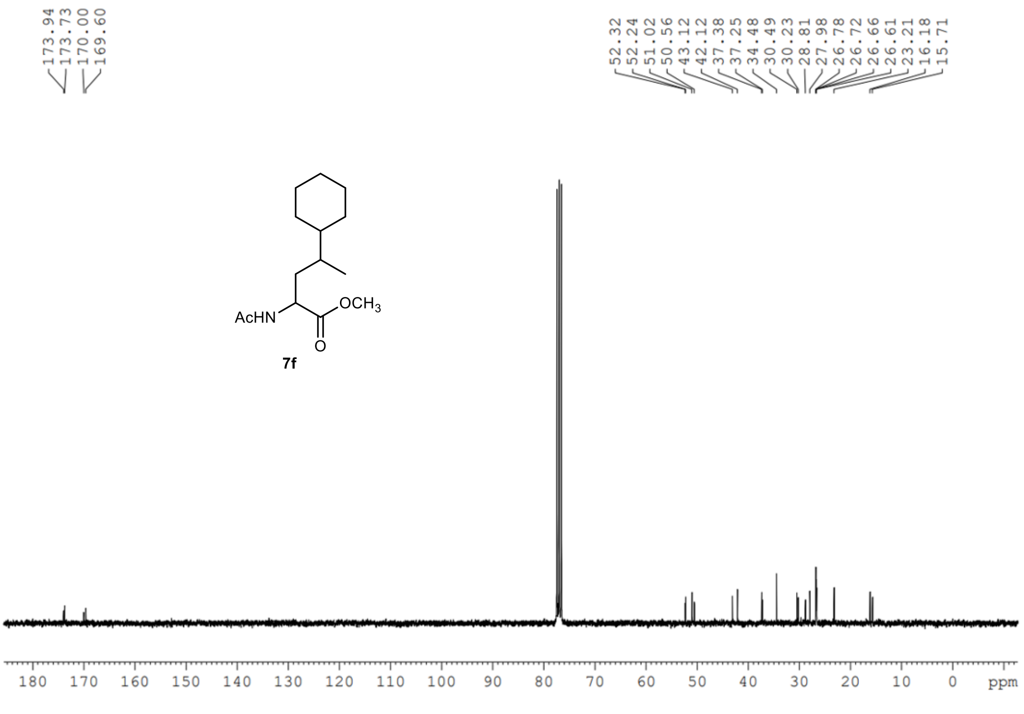

Supplementary Figure 50. ^{13}C NMR spectrum of compound **7f.**

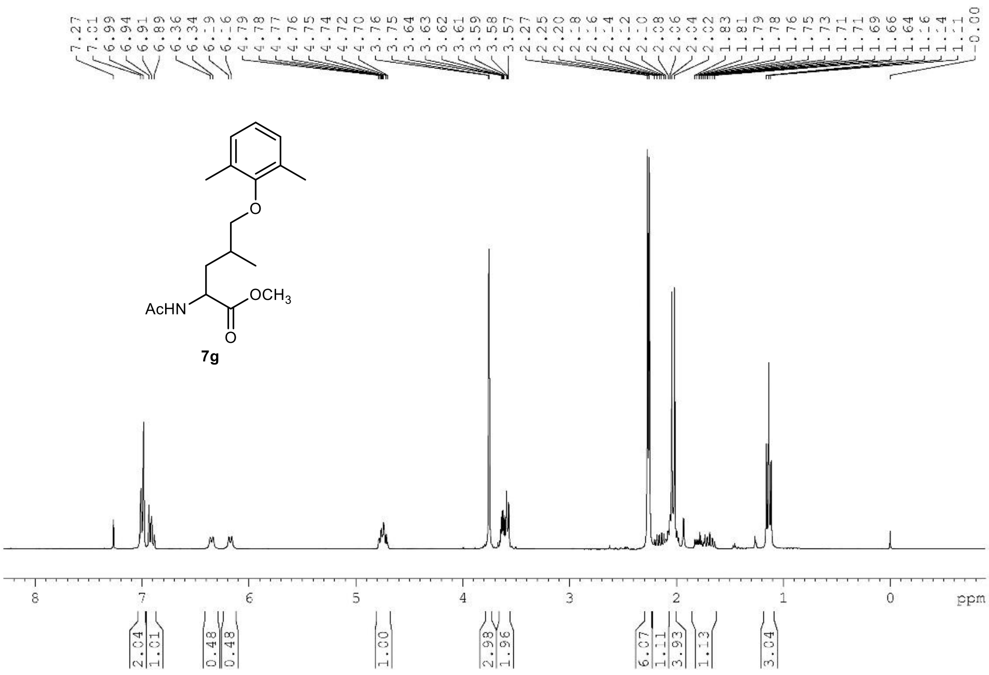

Supplementary Figure 51. ^{1}H NMR spectrum of compound **7g.**

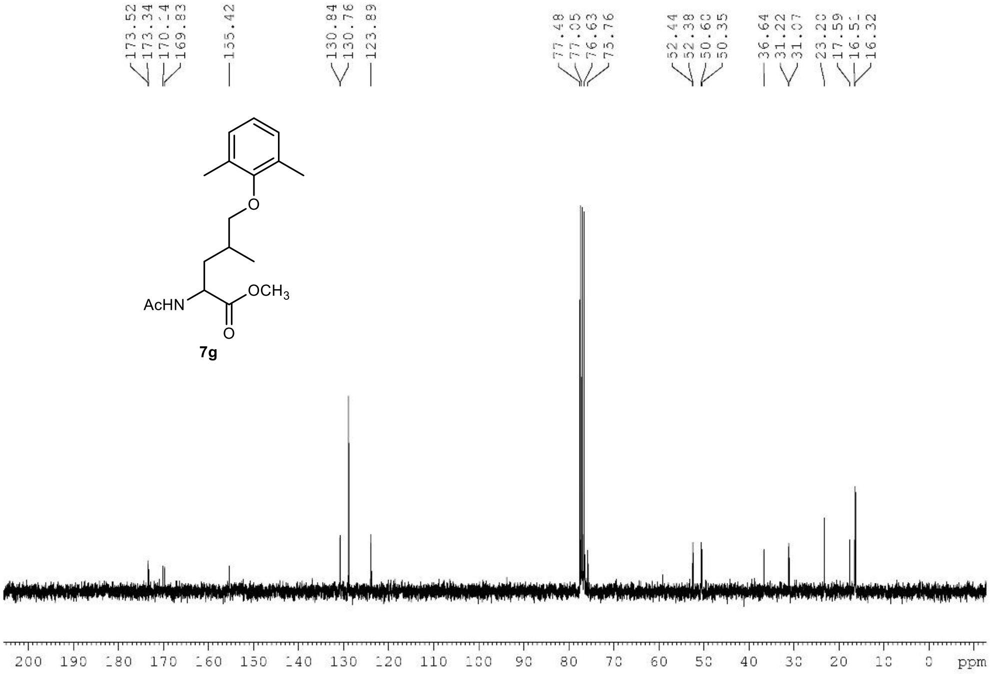

Supplementary Figure 52. ^{13}C NMR spectrum of compound **7g.**

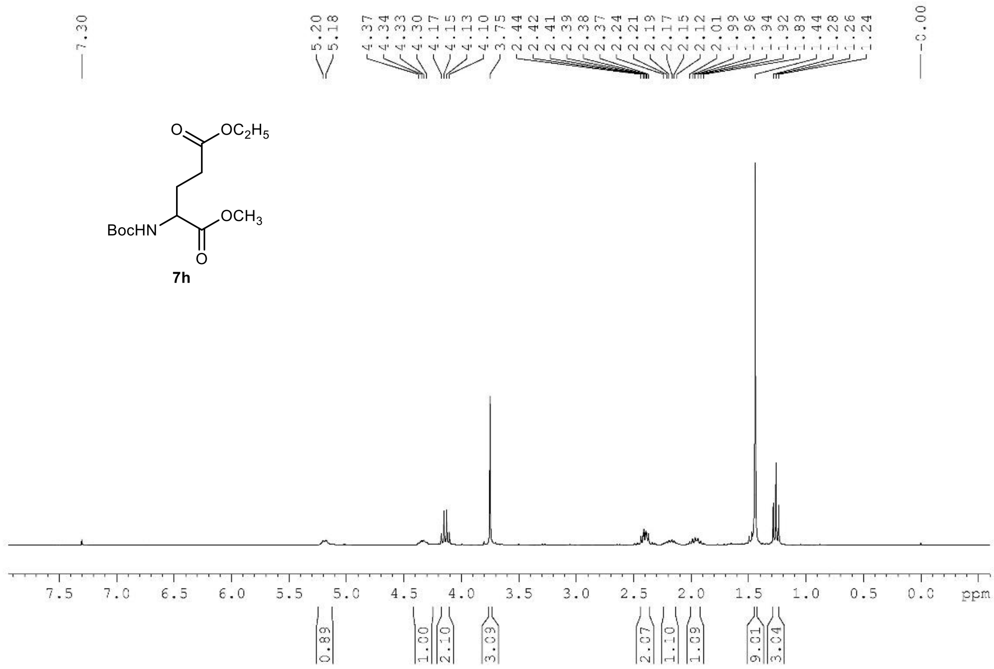

Supplementary Figure 53. ^{1}H NMR spectrum of compound **7h.**

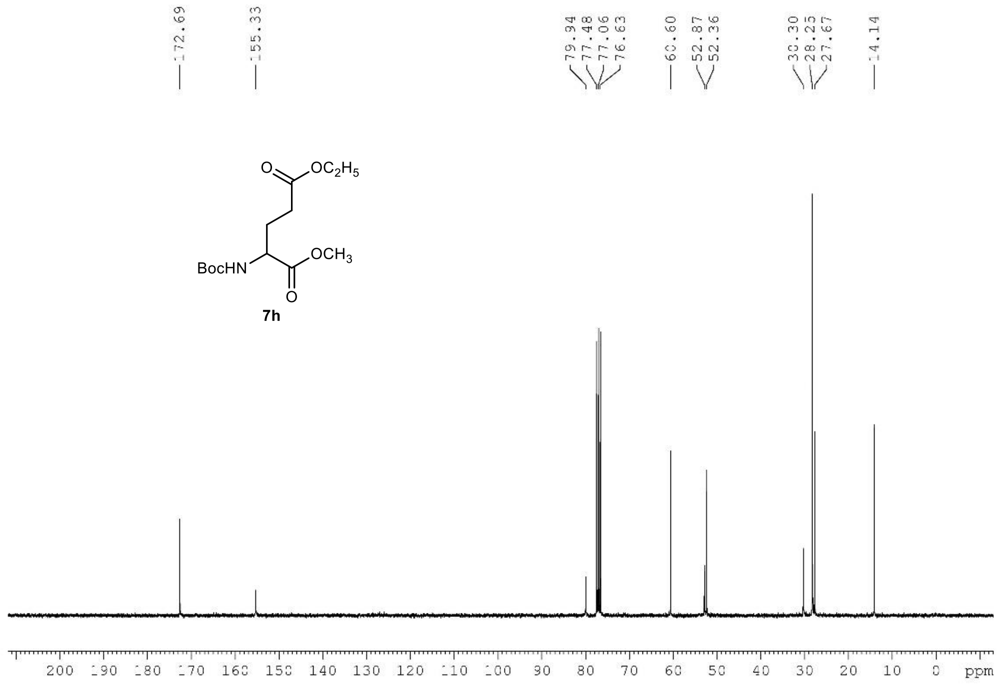

Supplementary Figure 54. ^{13}C NMR spectrum of compound **7h.**

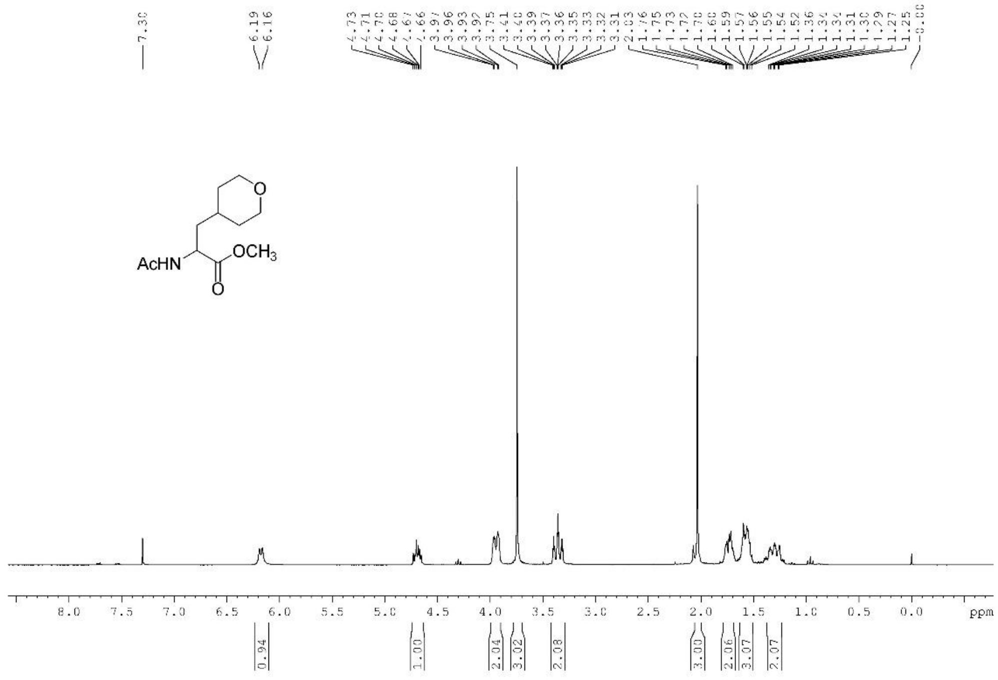

Supplementary Figure 55. ^{1}H NMR spectrum of compound **7i.**

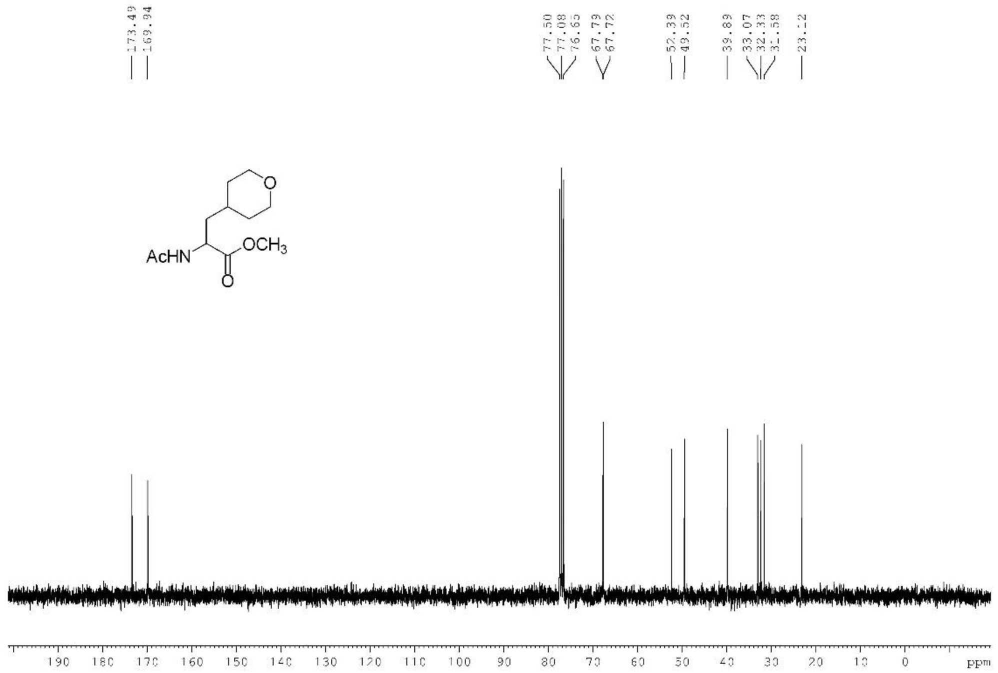

Supplementary Figure 56. ^{13}C NMR spectrum of compound **7i.**

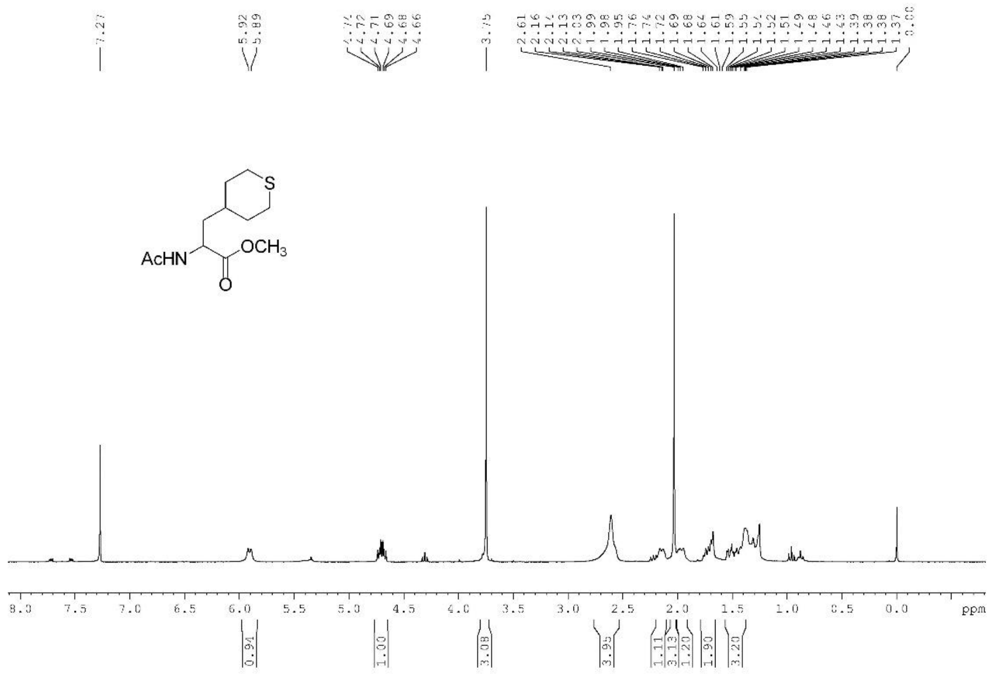

Supplementary Figure 57. ^{1}H NMR spectrum of compound **7j.**

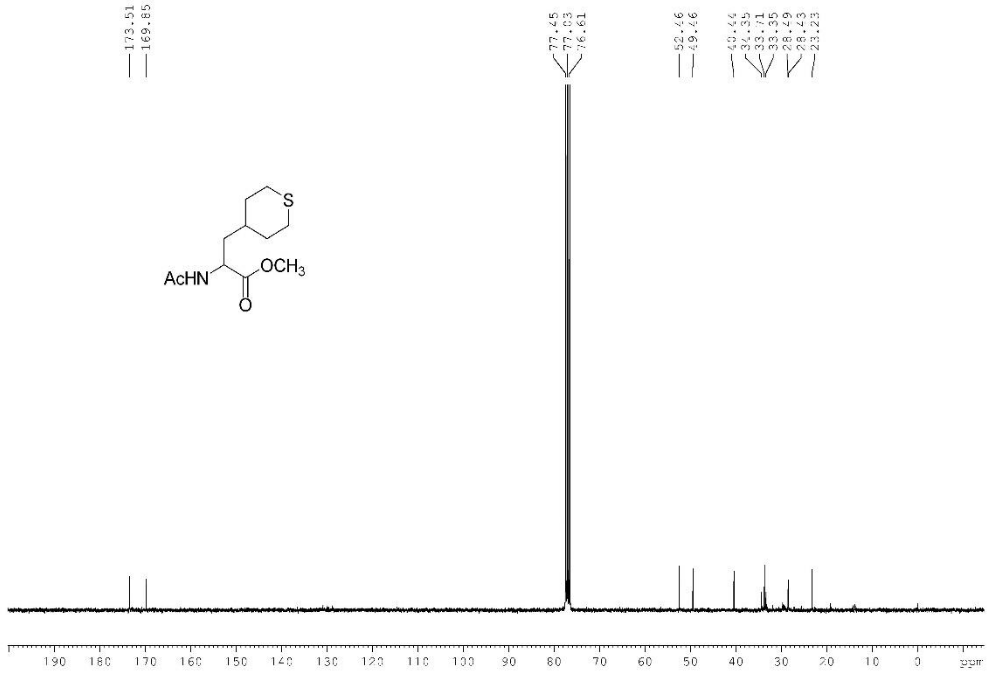

Supplementary Figure 58. ^{13}C NMR spectrum of compound **7j.**

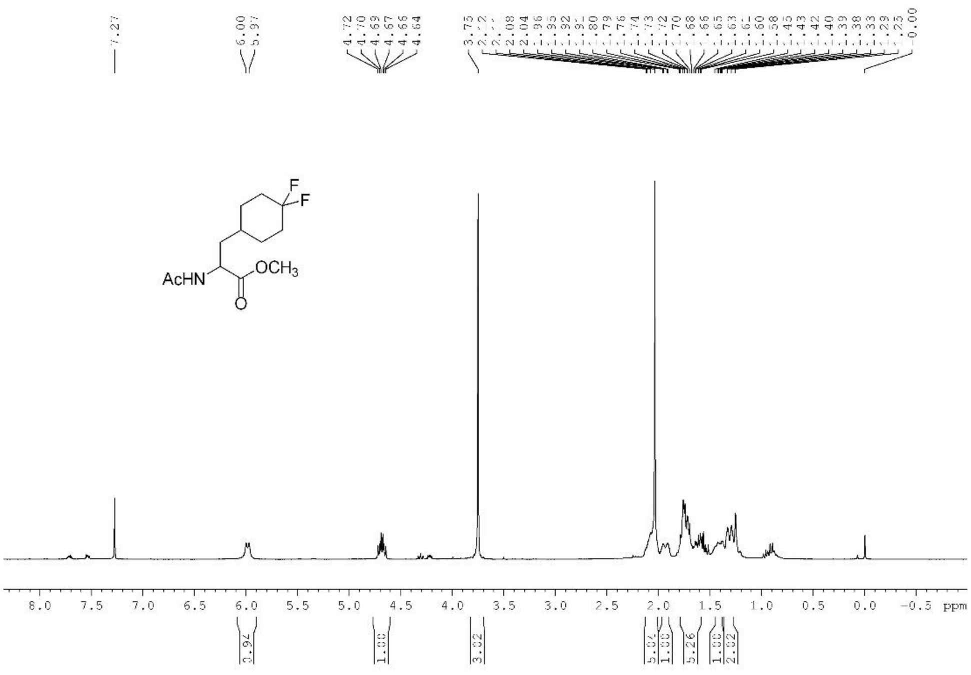

Supplementary Figure 59. ^{1}H NMR spectrum of compound **7k.**

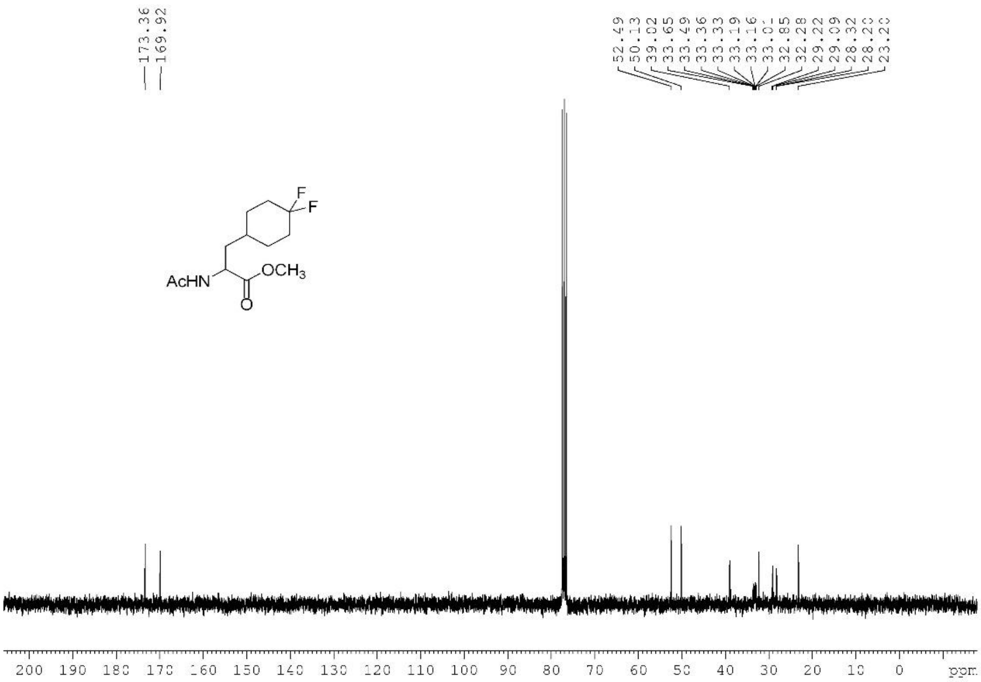

Supplementary Figure 60. ^{13}C NMR spectrum of compound **7k.**

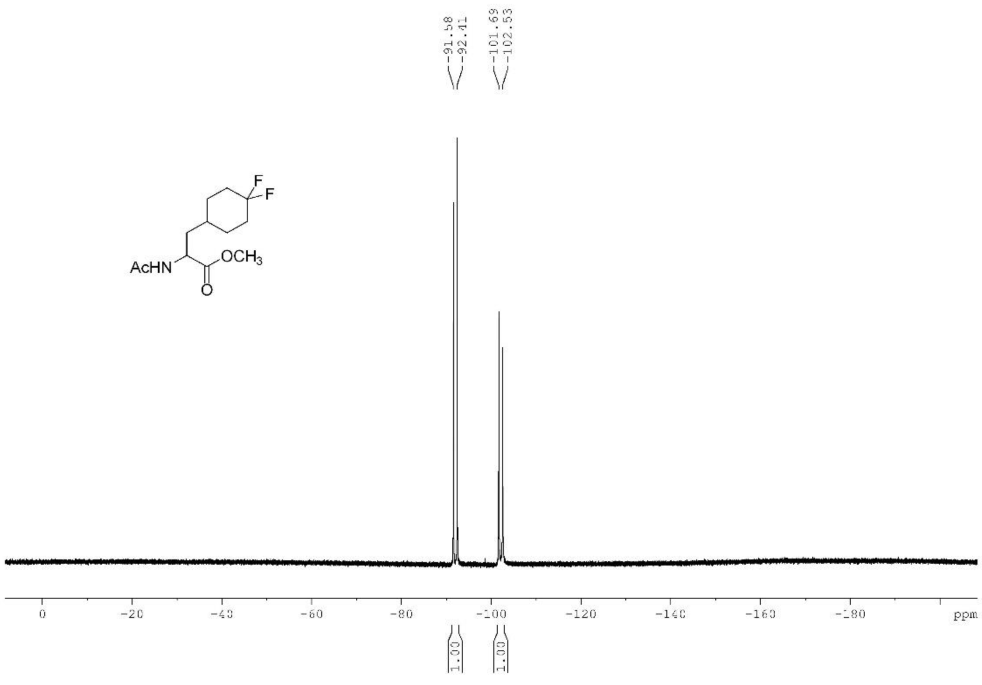

Supplementary Figure 61. ^{19}F NMR spectrum of compound **7k.**

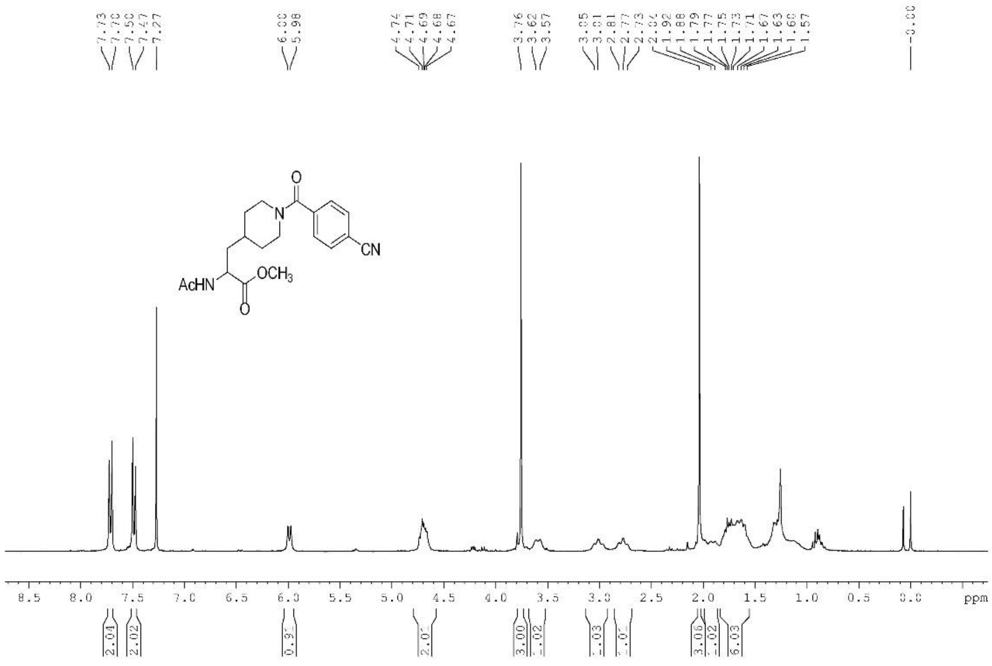

Supplementary Figure 62. ^{1}H NMR spectrum of compound **7l.**

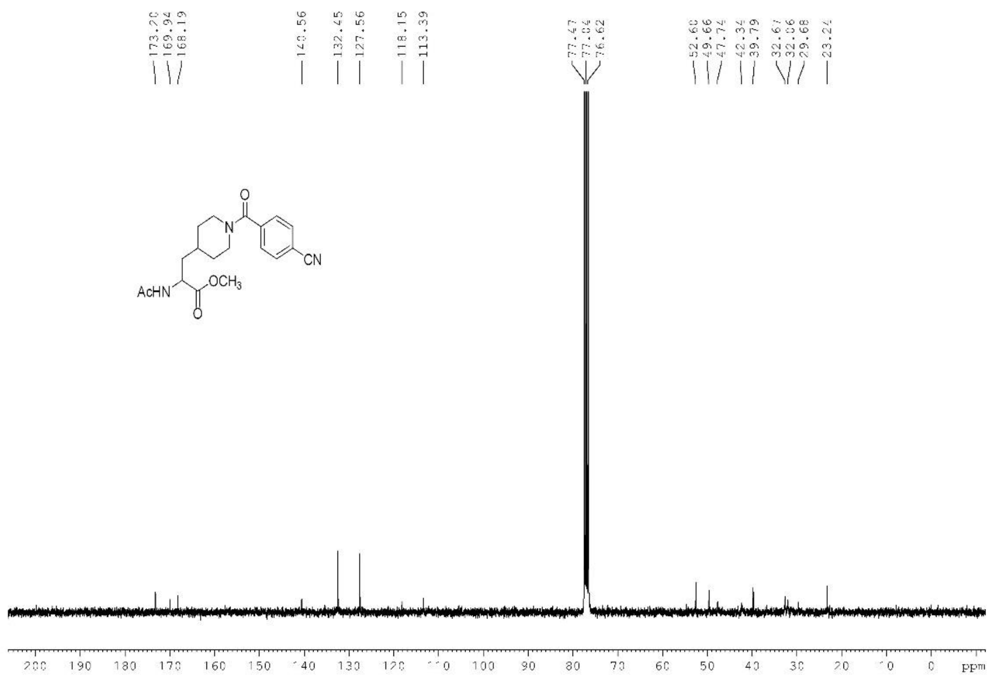

Supplementary Figure 63. ^{13}C NMR spectrum of compound **7l.**

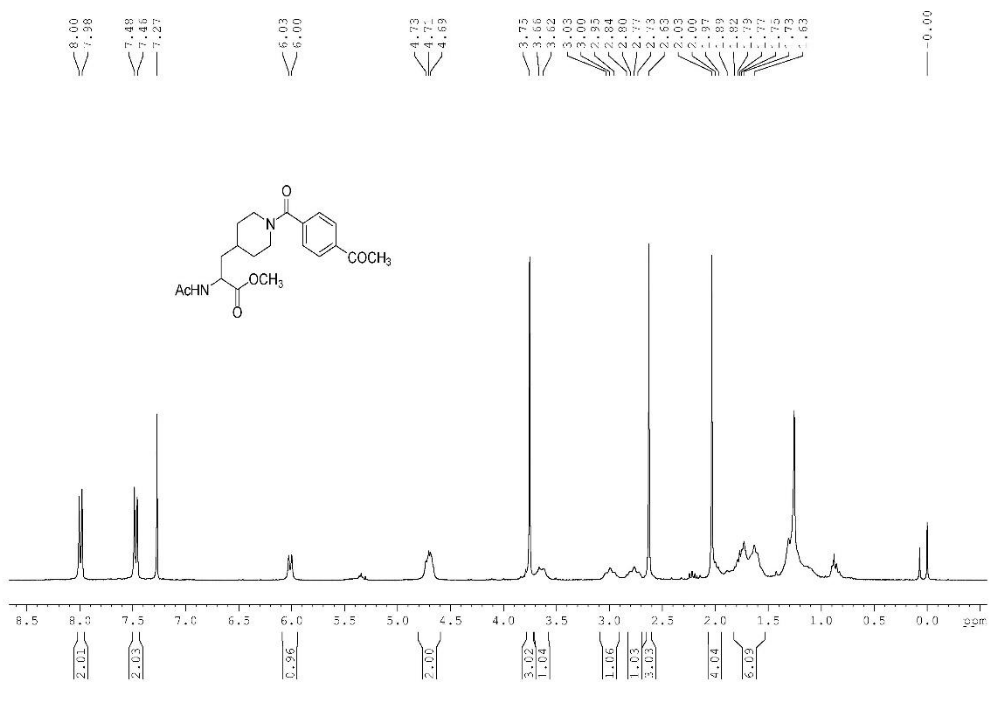

Supplementary Figure 64. ^{1}H NMR spectrum of compound **7m.**

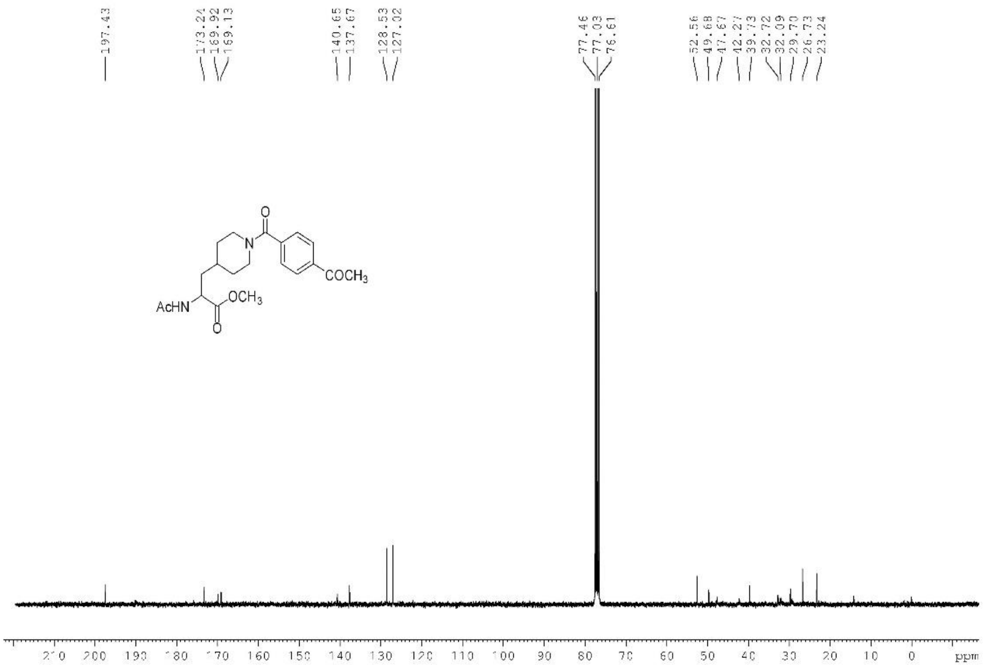

Supplementary Figure 65. ^{13}C NMR spectrum of compound **7m.**

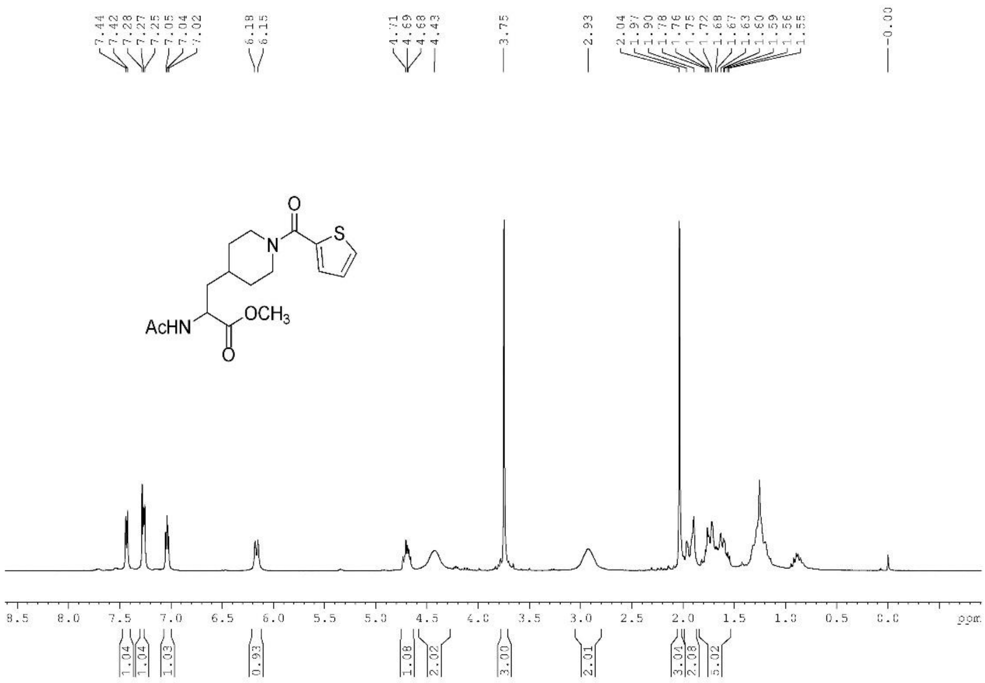

Supplementary Figure 66. ^{1}H NMR spectrum of compound **7n.**

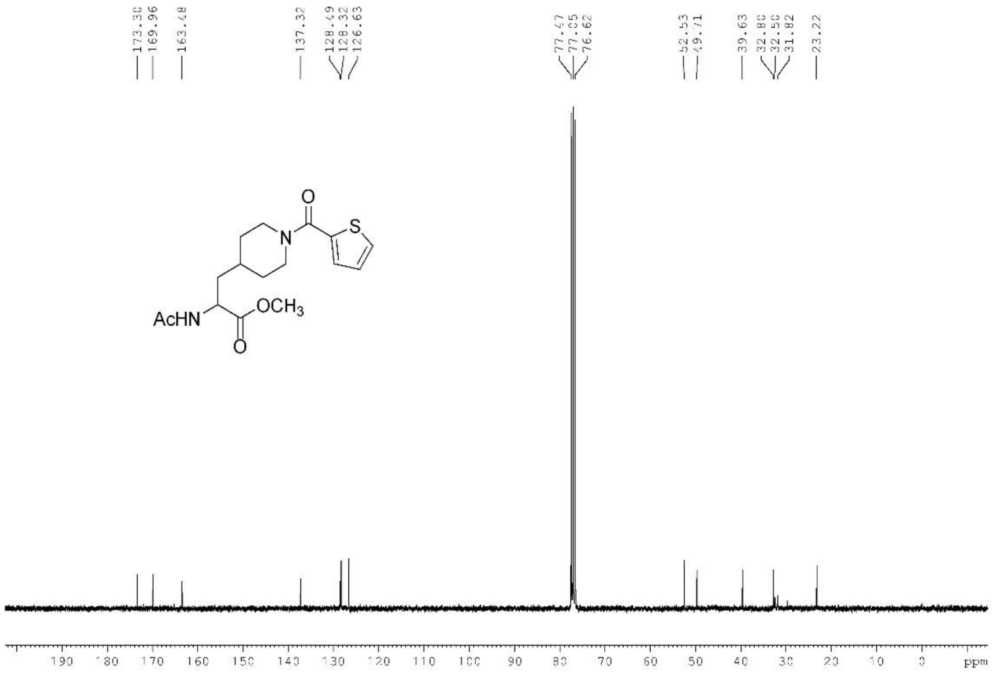

Supplementary Figure 67. ^{13}C NMR spectrum of compound **7n.**

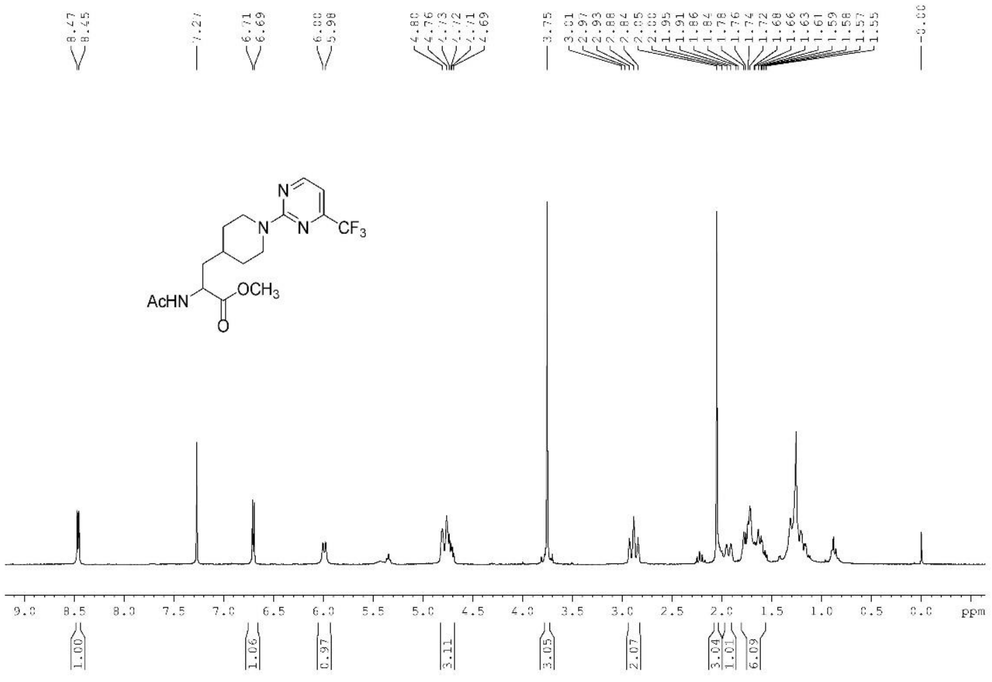

Supplementary Figure 68. ^{1}H NMR spectrum of compound **7o.**

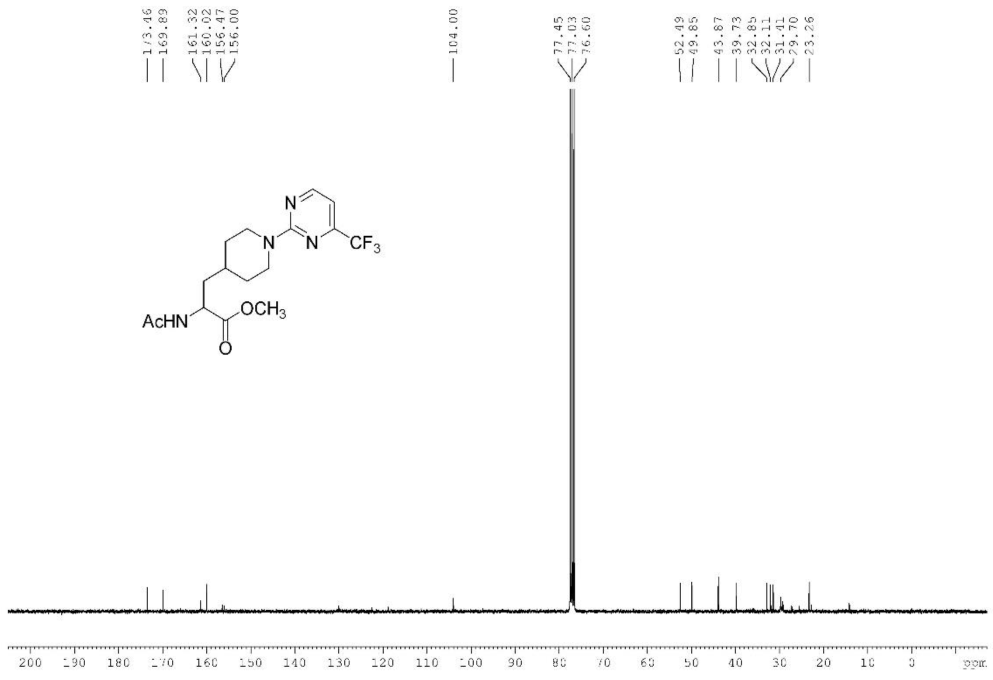

Supplementary Figure 69. ^{13}C NMR spectrum of compound **7o.**

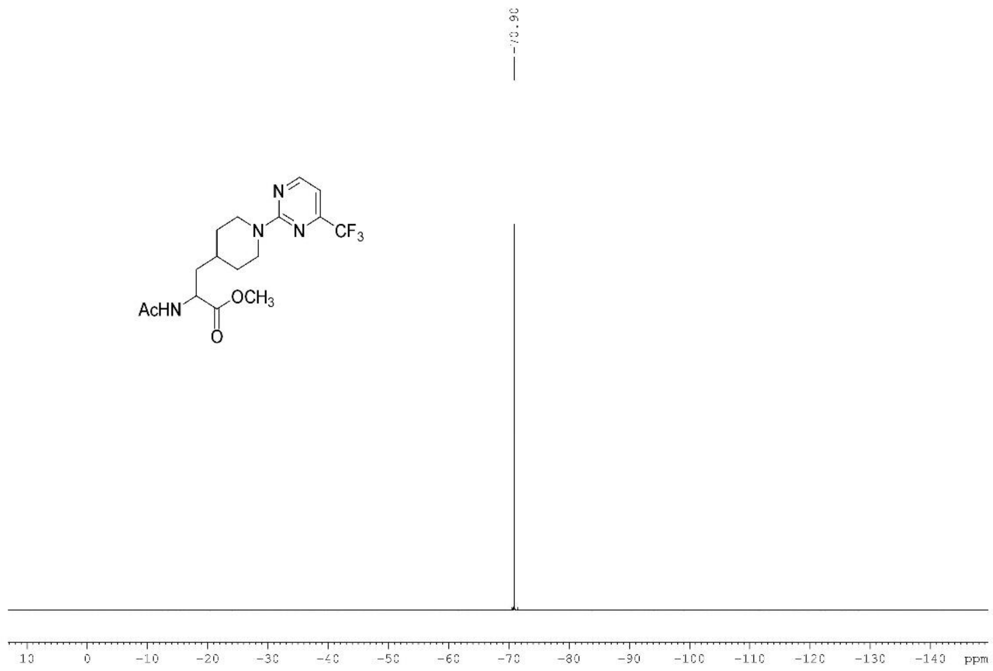

Supplementary Figure 70. ^{19}F NMR spectrum of compound **7o.**

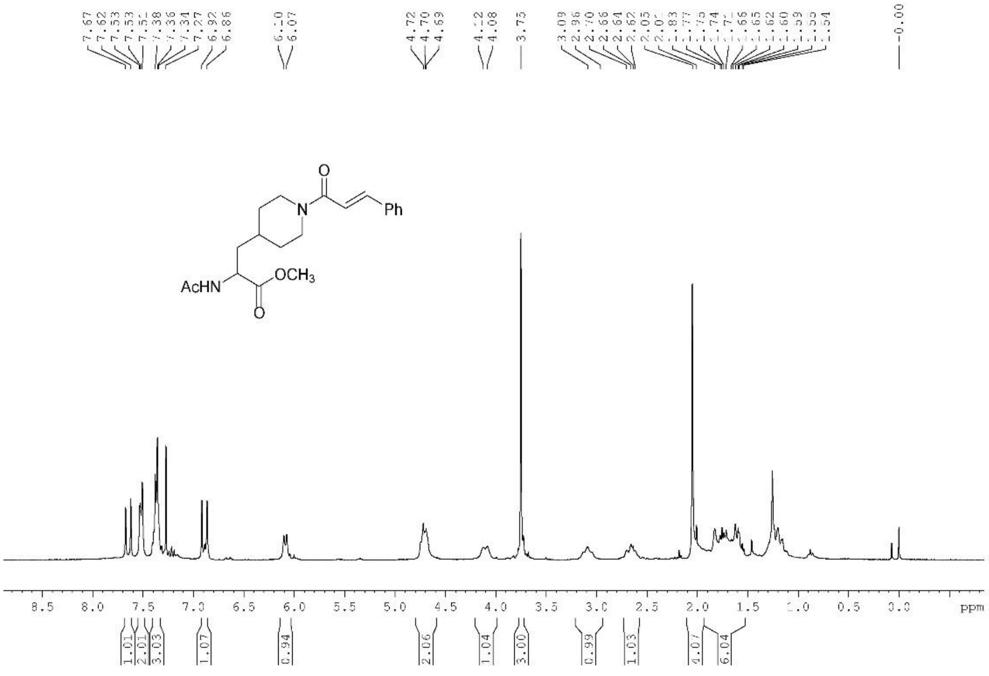

Supplementary Figure 71. ^{1}H NMR spectrum of compound **7p.**

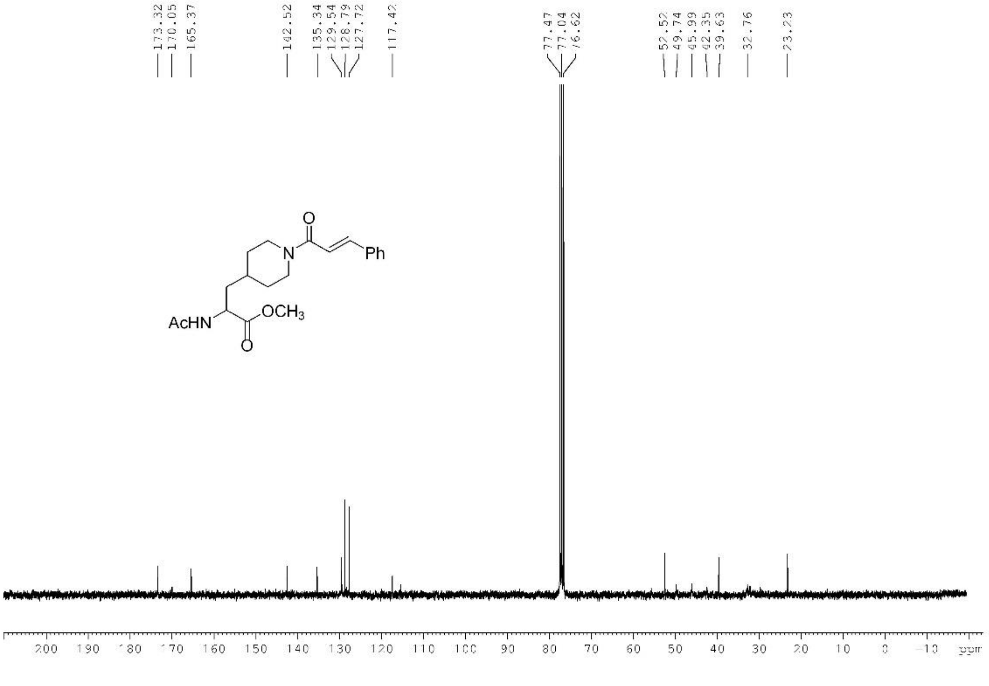

Supplementary Figure 72. ^{13}C NMR spectrum of compound **7p.**

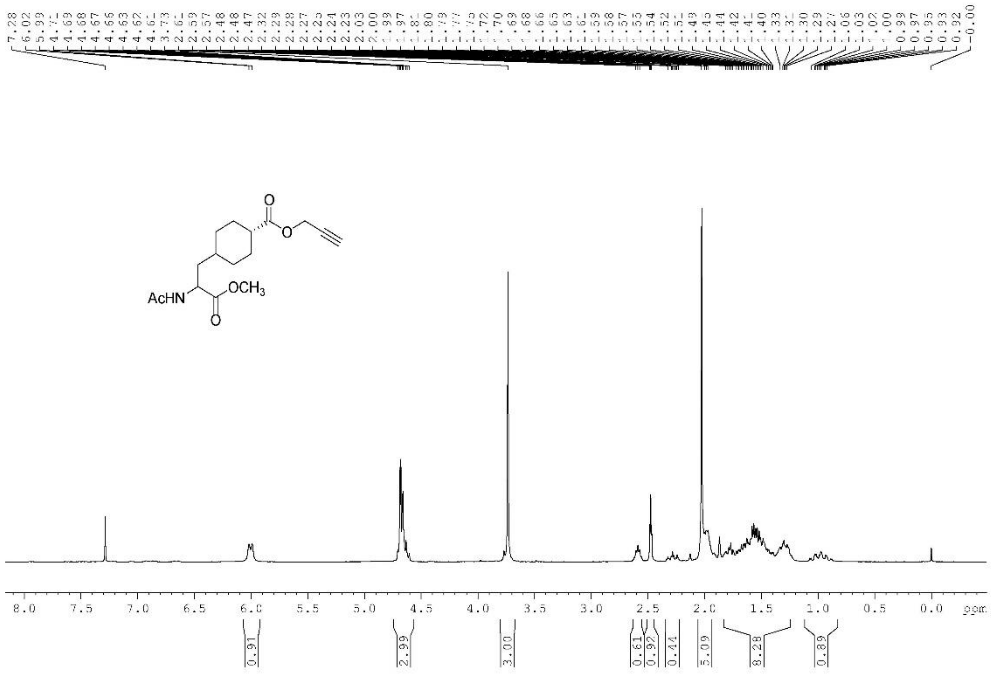

Supplementary Figure 73. ^{1}H NMR spectrum of compound **7q.**

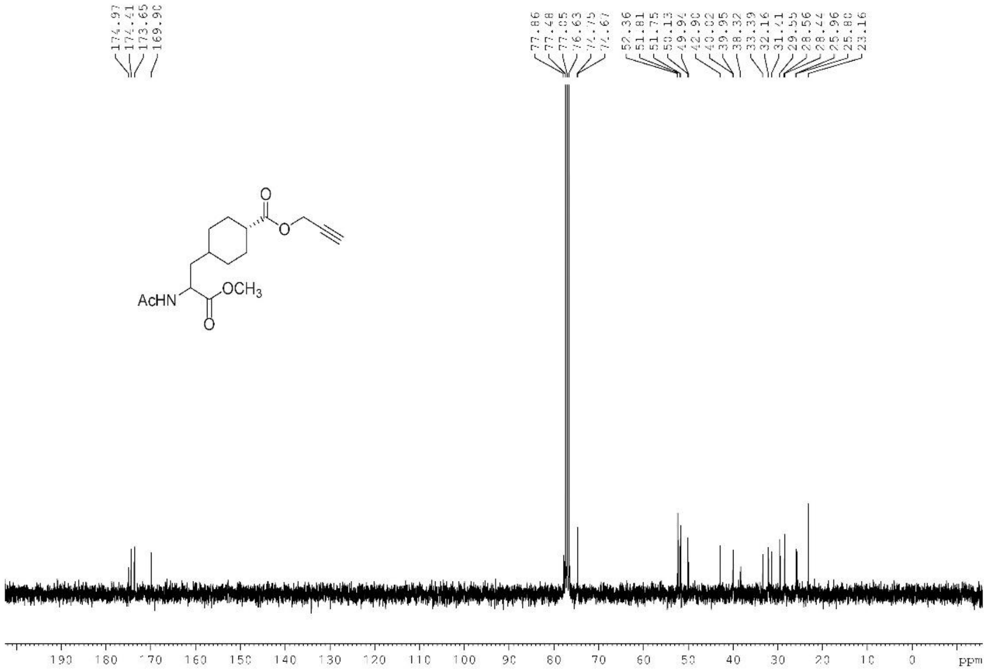

Supplementary Figure 74. ^{13}C NMR spectrum of compound **7q.**

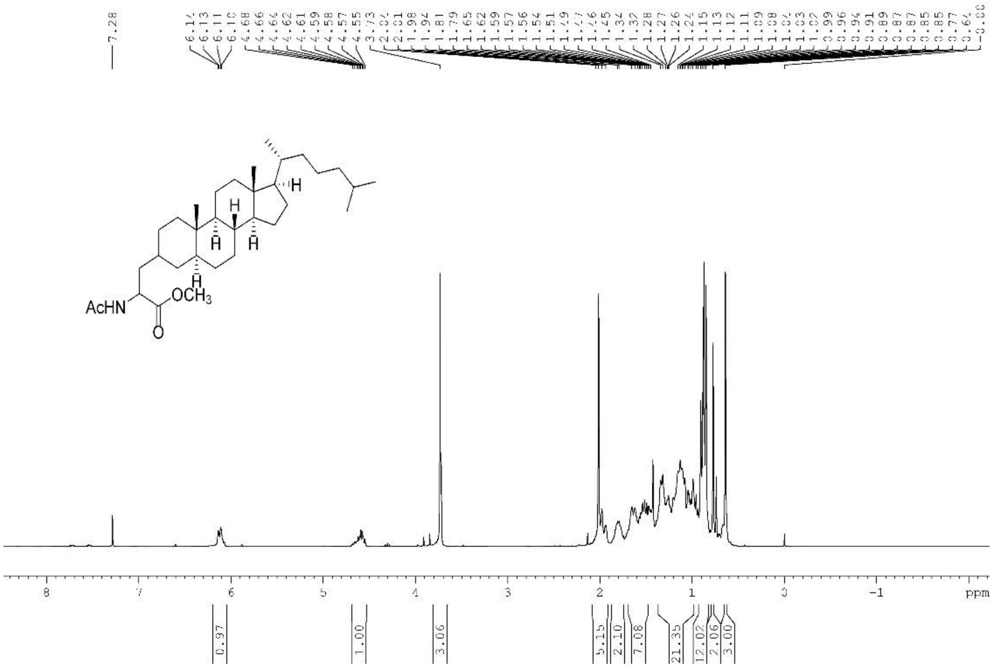

Supplementary Figure 75. ^{1}H NMR spectrum of compound **7r.**

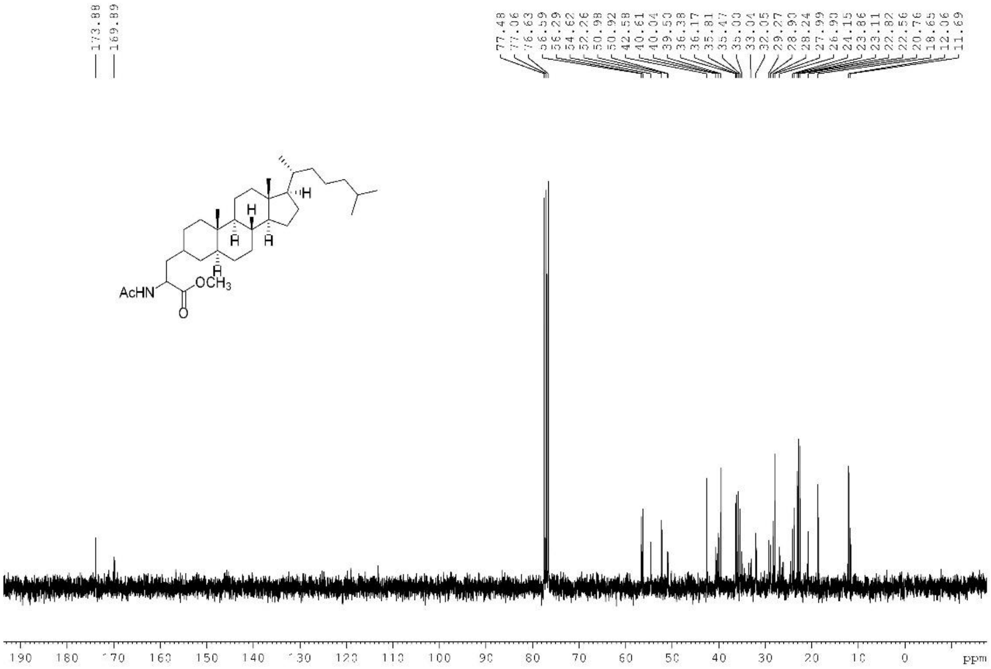

Supplementary Figure 76. ^{13}C NMR spectrum of compound **7r.**

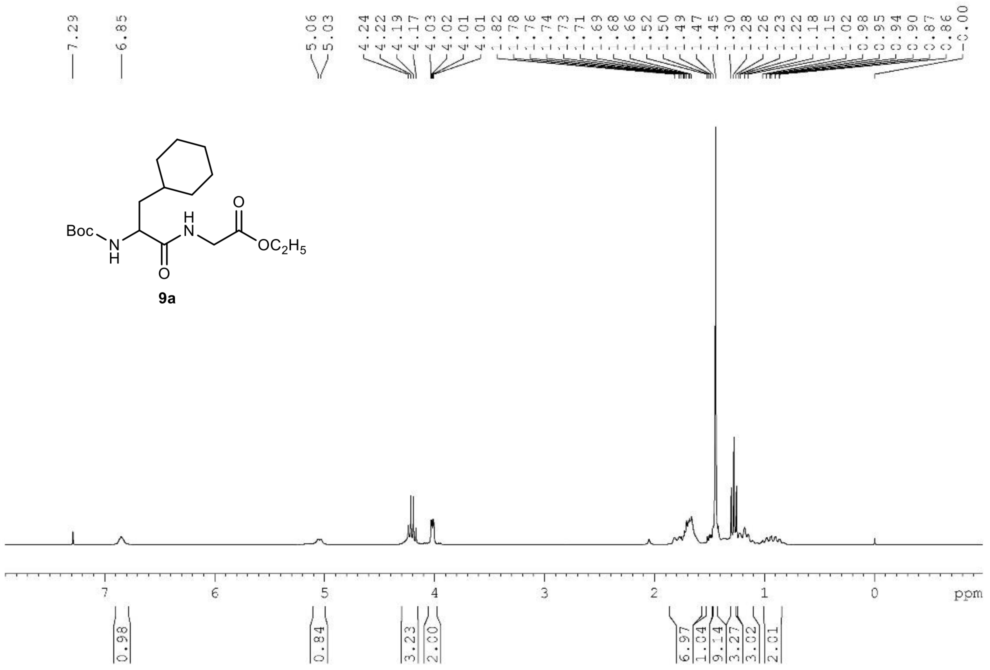

Supplementary Figure 77. ^{1}H NMR spectrum of compound **9a.**

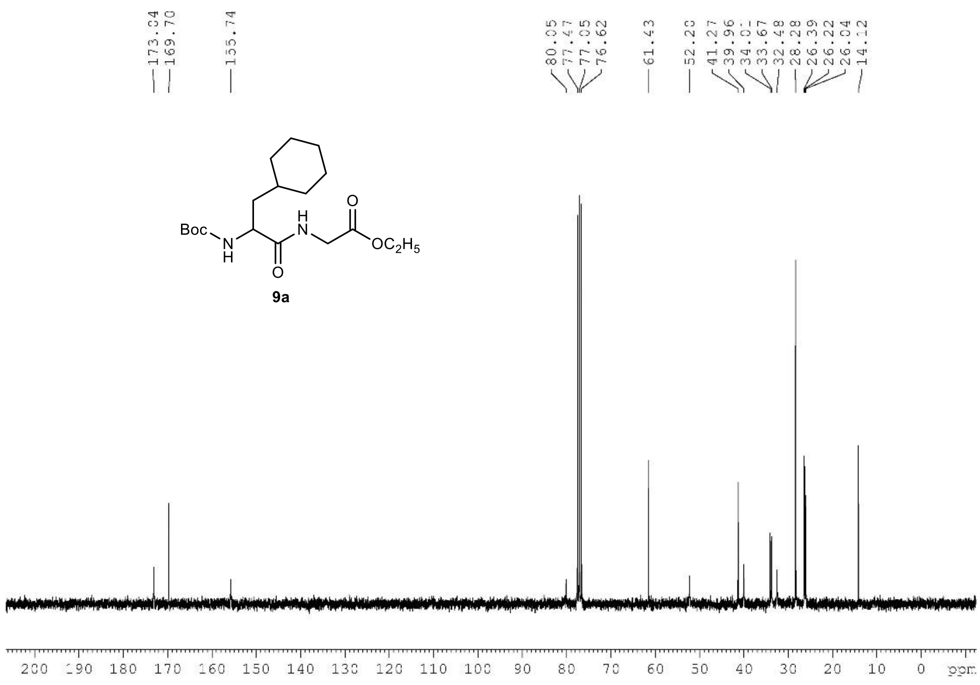

Supplementary Figure 78. ^{13}C NMR spectrum of compound **9a.**

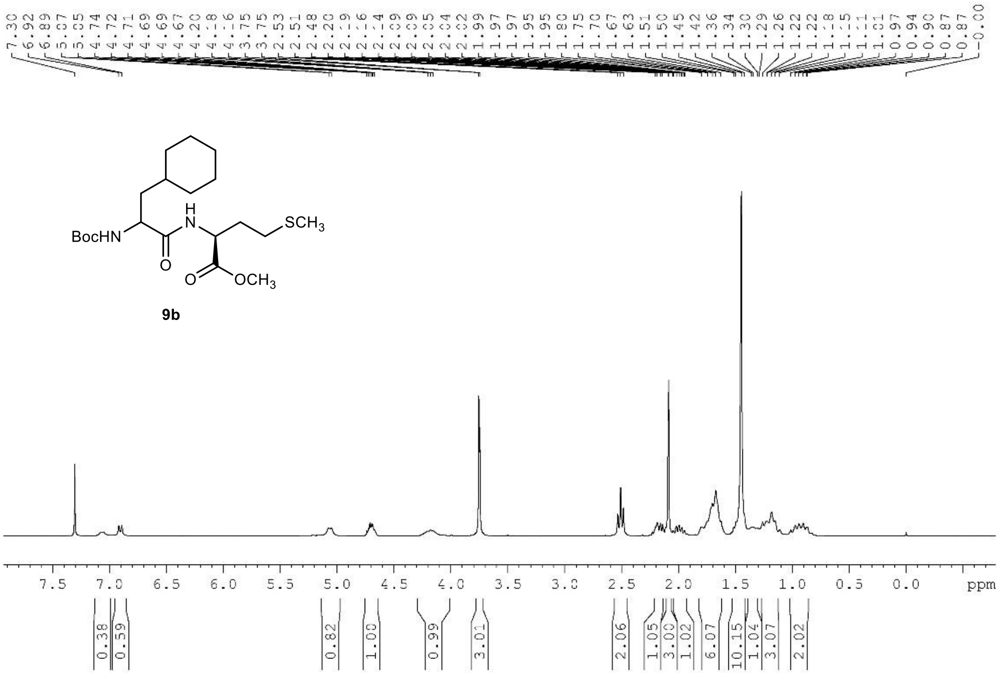

Supplementary Figure 79. ^{1}H NMR spectrum of compound **9b.**

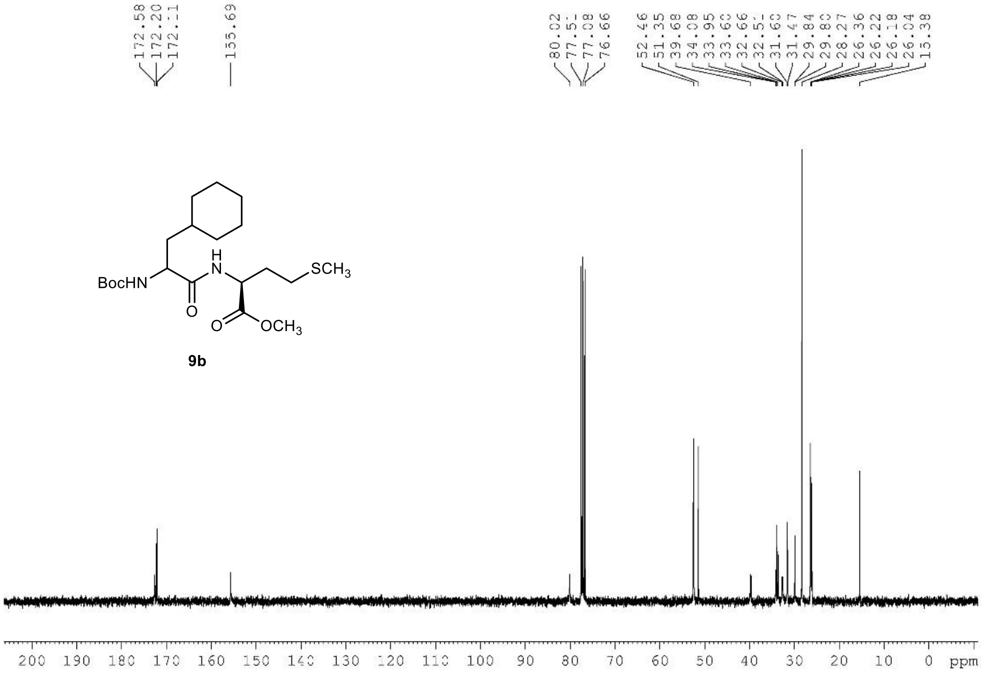

Supplementary Figure 80. ^{13}C NMR spectrum of compound **9b.**

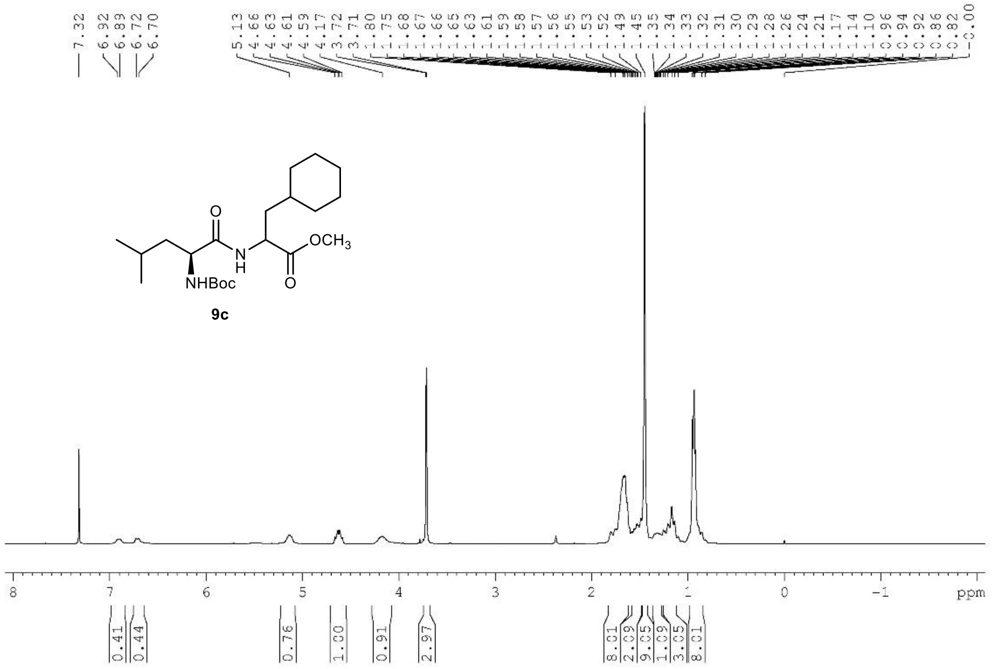

Supplementary Figure 81. ^{1}H NMR spectrum of compound **9c.**

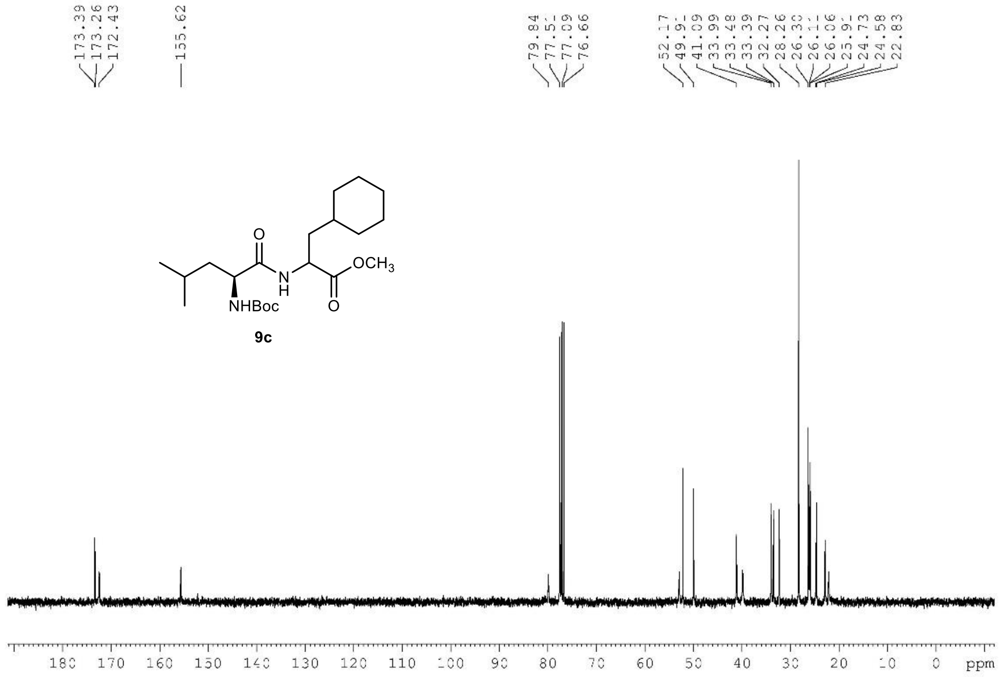

Supplementary Figure 82. ^{13}C NMR spectrum of compound **9c.**

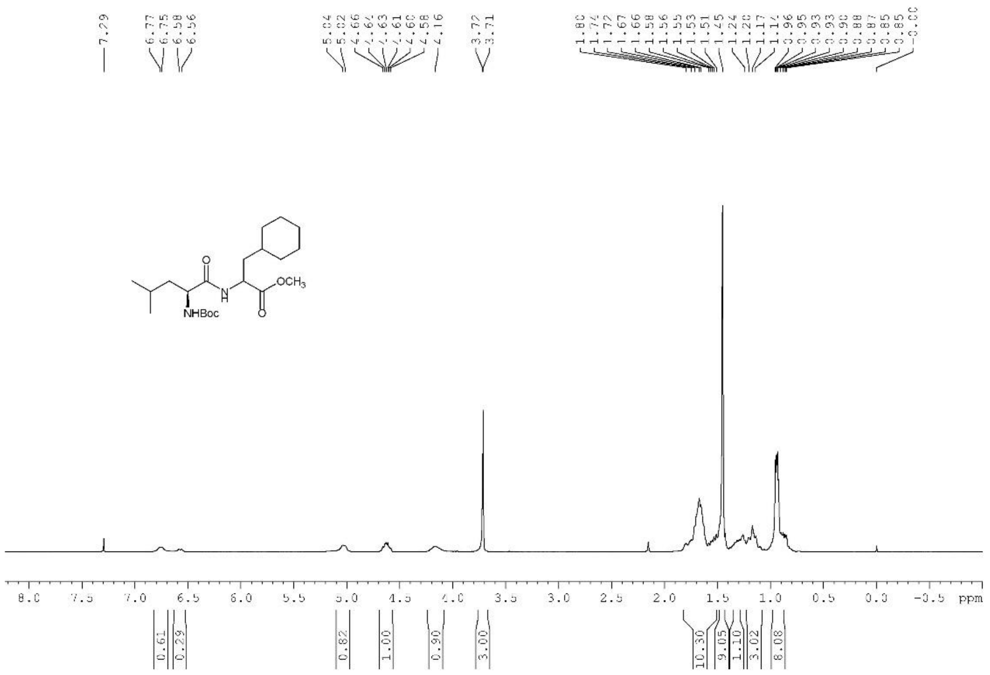

Supplementary Figure 83. ^{1}H NMR spectrum of compound **9c** (Condition B).

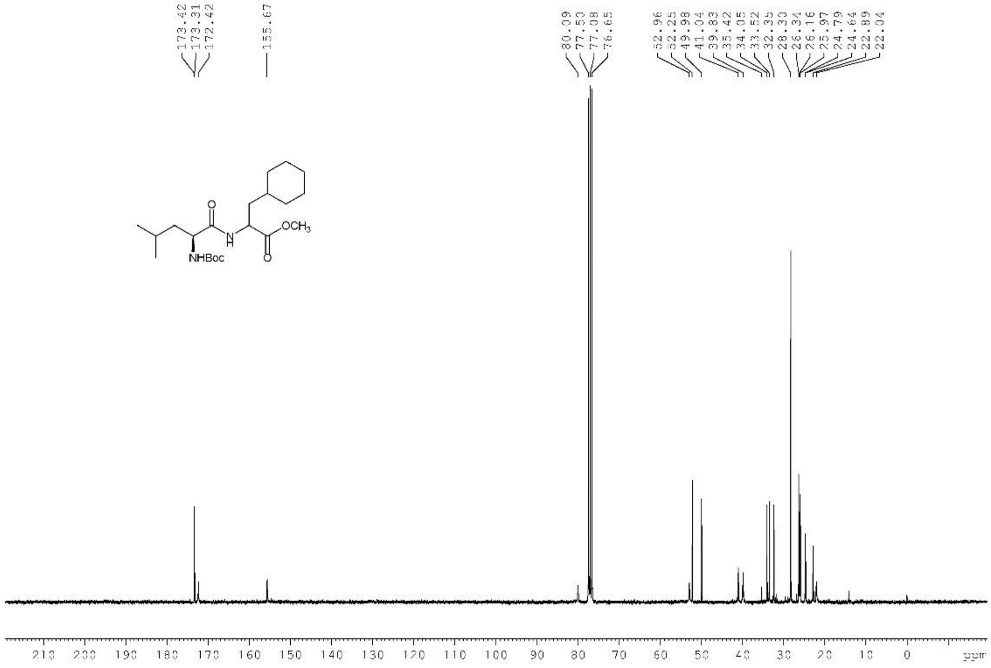

Supplementary Figure 84. ^{13}C NMR spectrum of compound **9c** (Condition B).

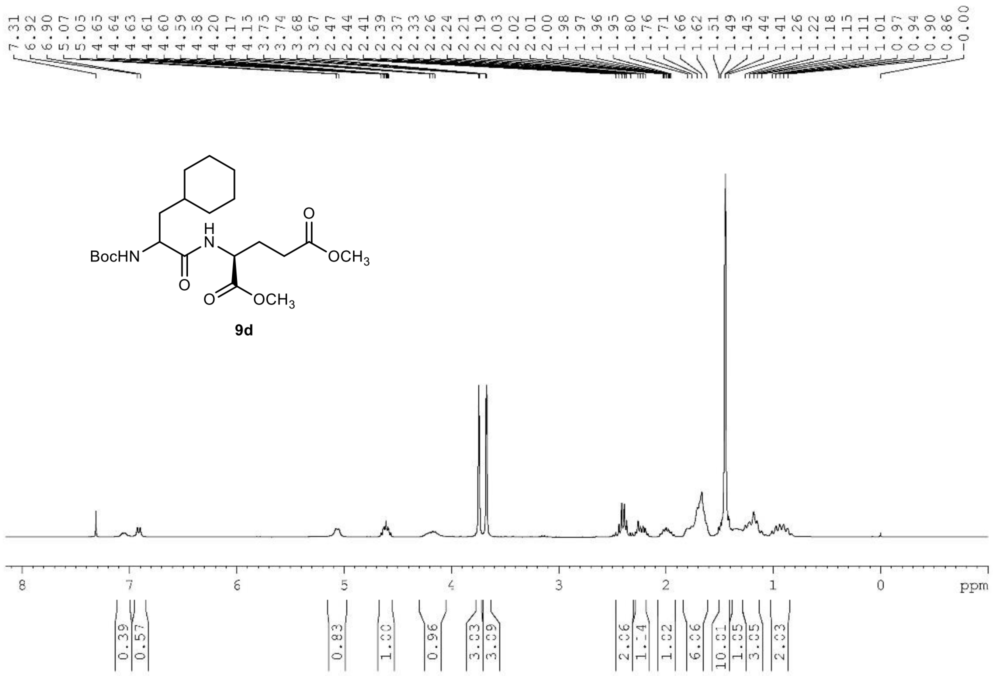

Supplementary Figure 85. ^{1}H NMR spectrum of compound **9d.**

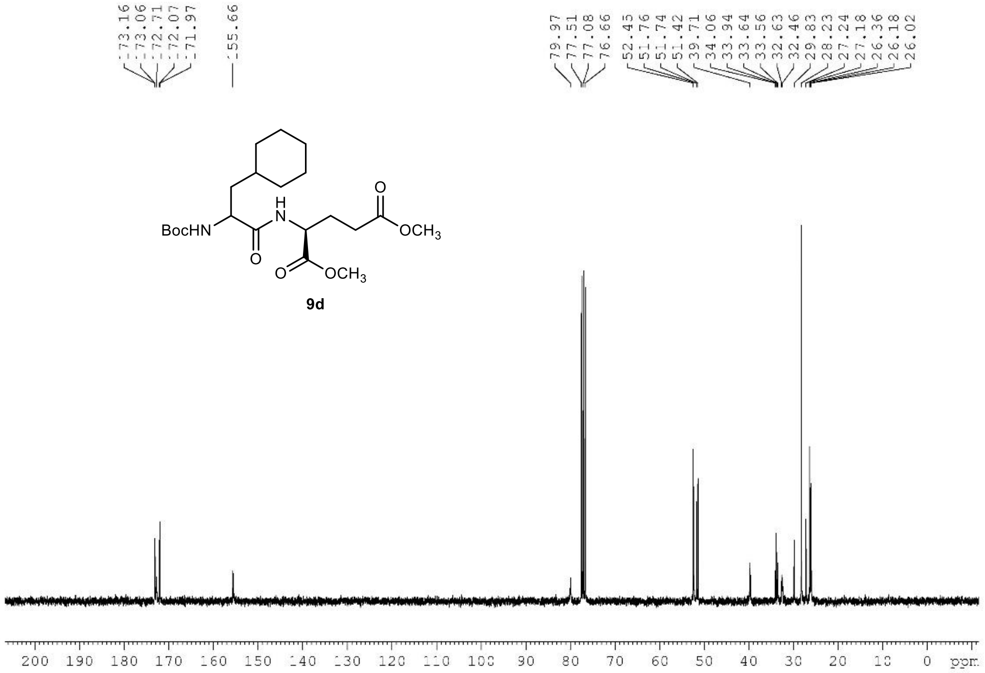

Supplementary Figure 86. ^{13}C NMR spectrum of compound **9d.**

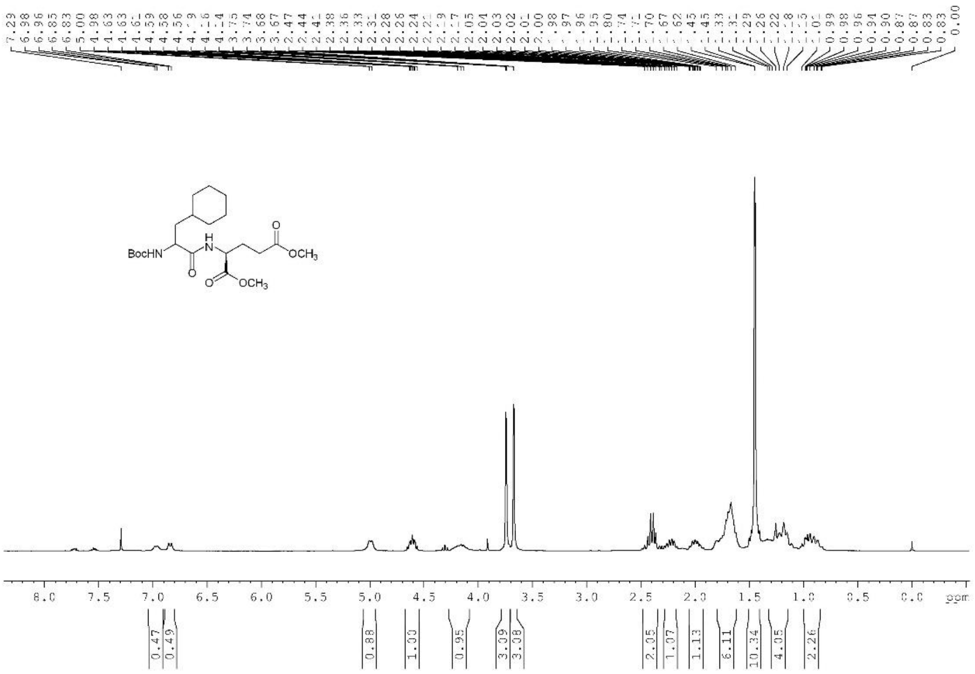

Supplementary Figure 87. ^{1}H NMR spectrum of compound **9d** (Condition B).

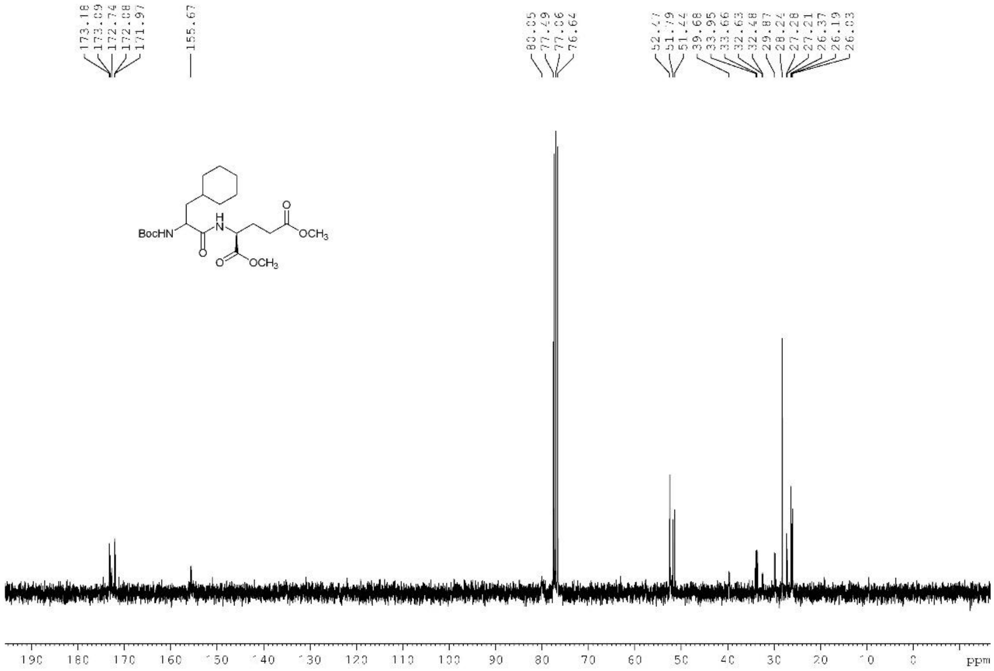

Supplementary Figure 88. ^{13}C NMR spectrum of compound **9d** (Condition B).

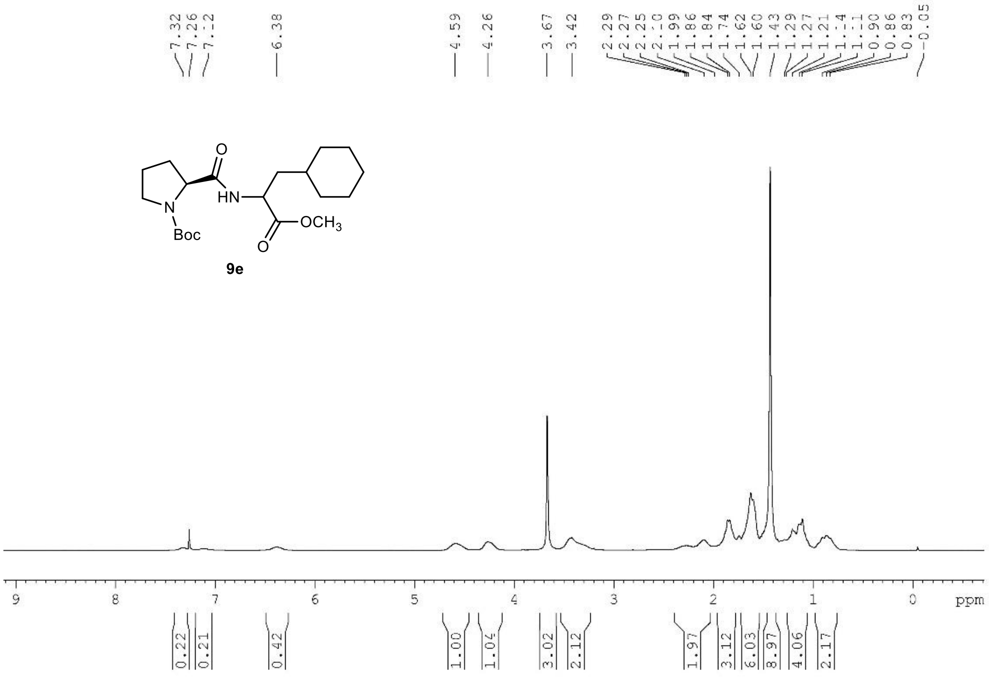

Supplementary Figure 89. ^{1}H NMR spectrum of compound **9e.**

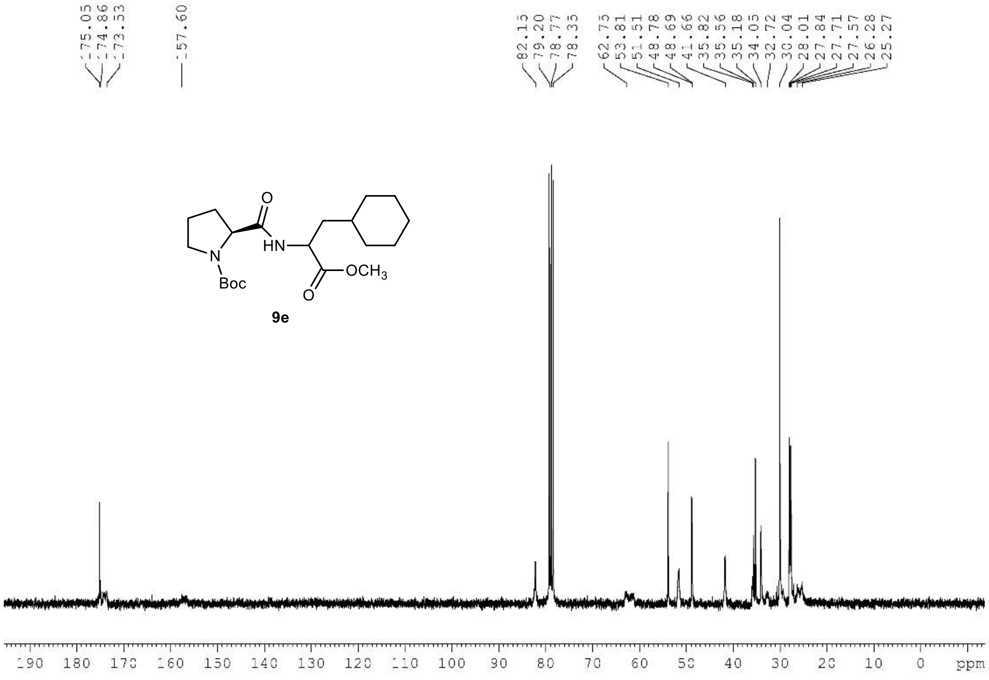

Supplementary Figure 90. ^{13}C NMR spectrum of compound **9e.**

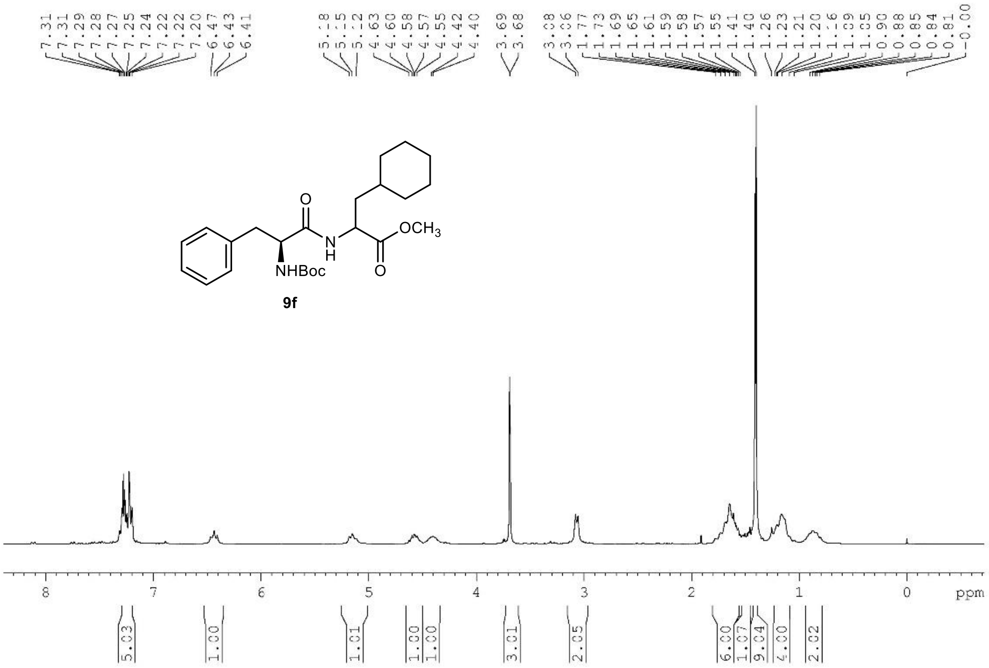

Supplementary Figure 91. ^{1}H NMR spectrum of compound **9f.**

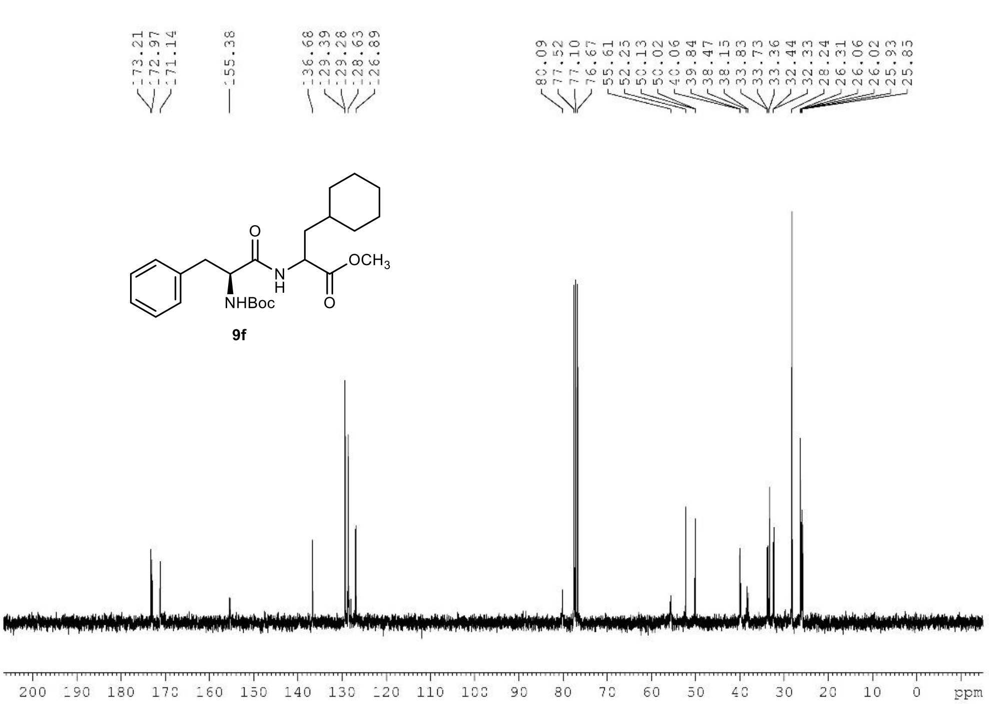

Supplementary Figure 92. ^{13}C NMR spectrum of compound **9f.**

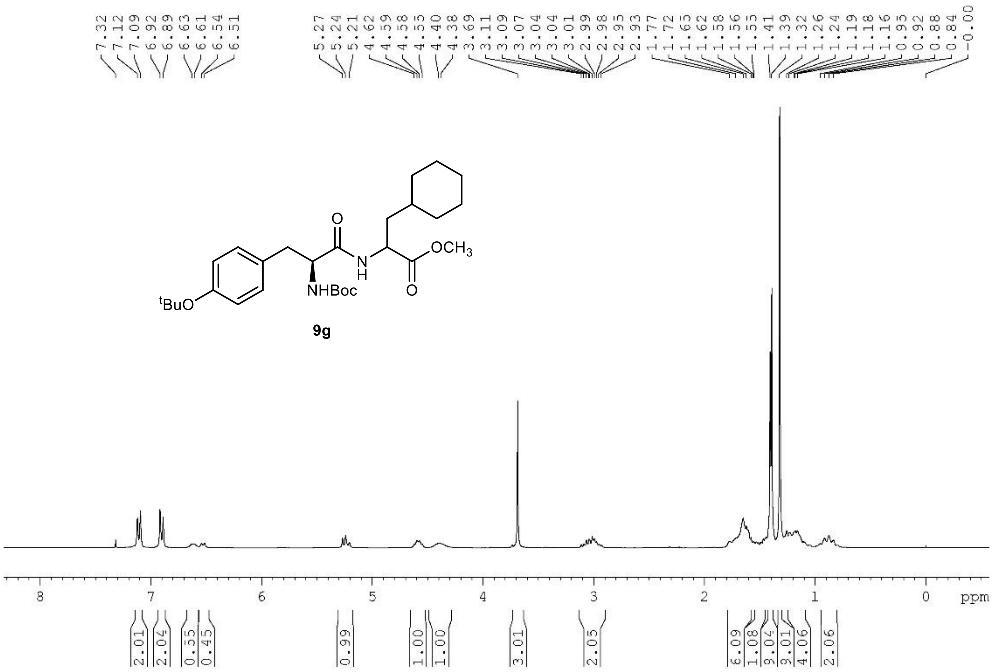

Supplementary Figure 93. ^{1}H NMR spectrum of compound **9g.**

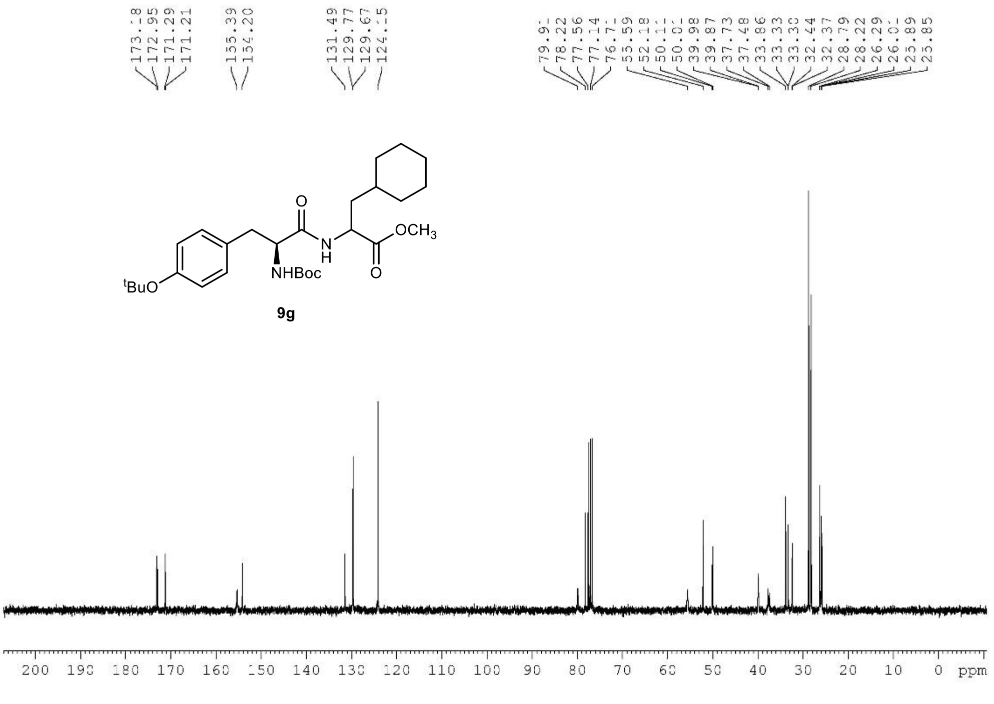

Supplementary Figure 94. ^{13}C NMR spectrum of compound **9g.**

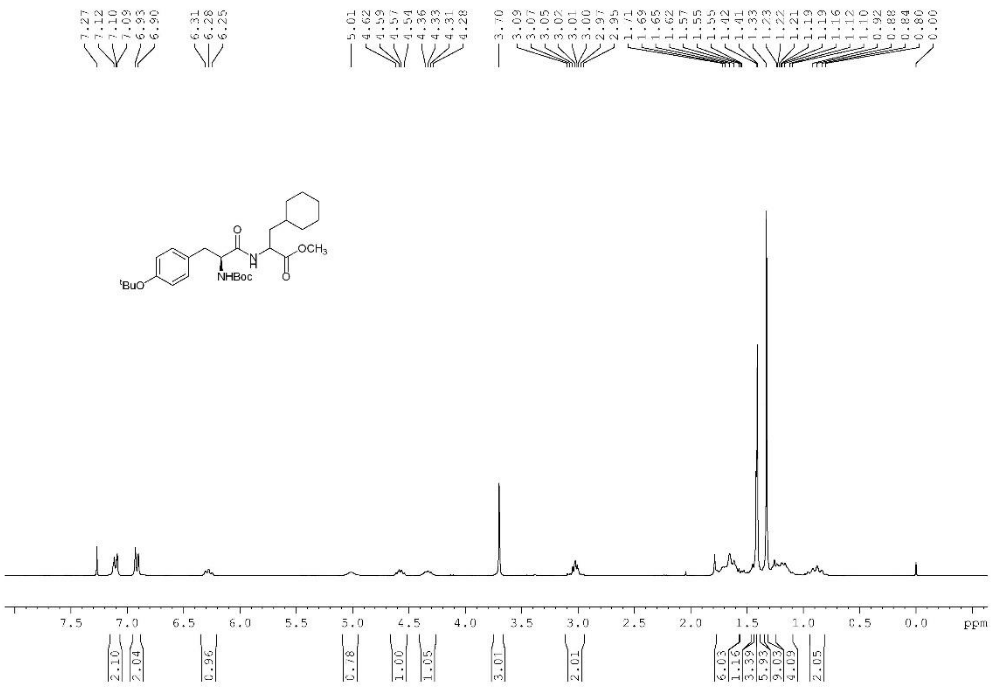

Supplementary Figure 95. ^{1}H NMR spectrum of compound **9g** (Condition B).

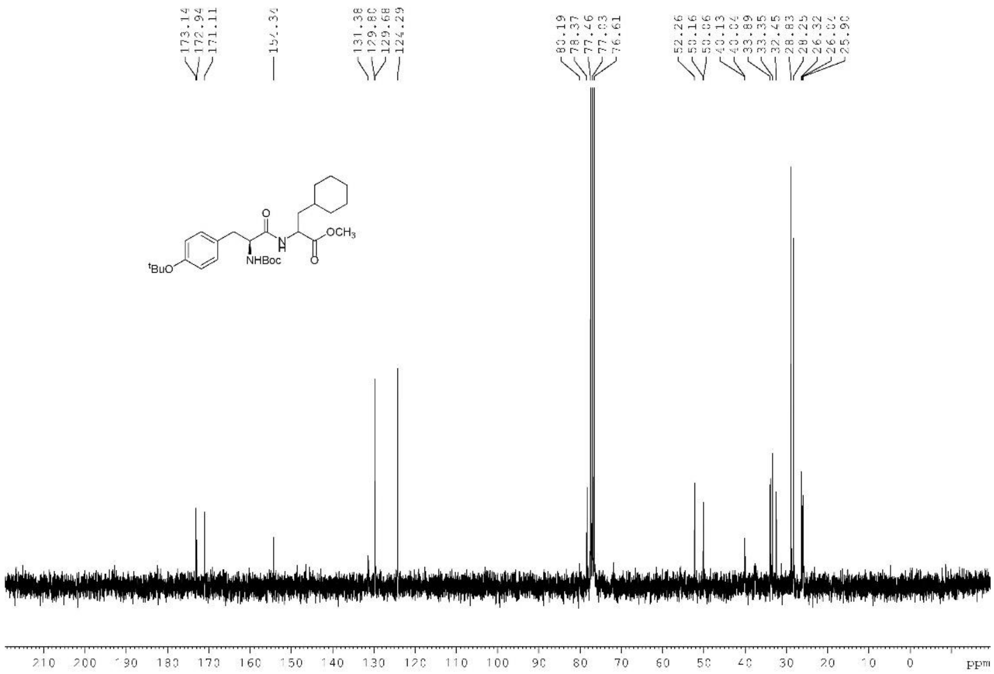

Supplementary Figure 96. ^{13}C NMR spectrum of compound **9g** (Condition B).

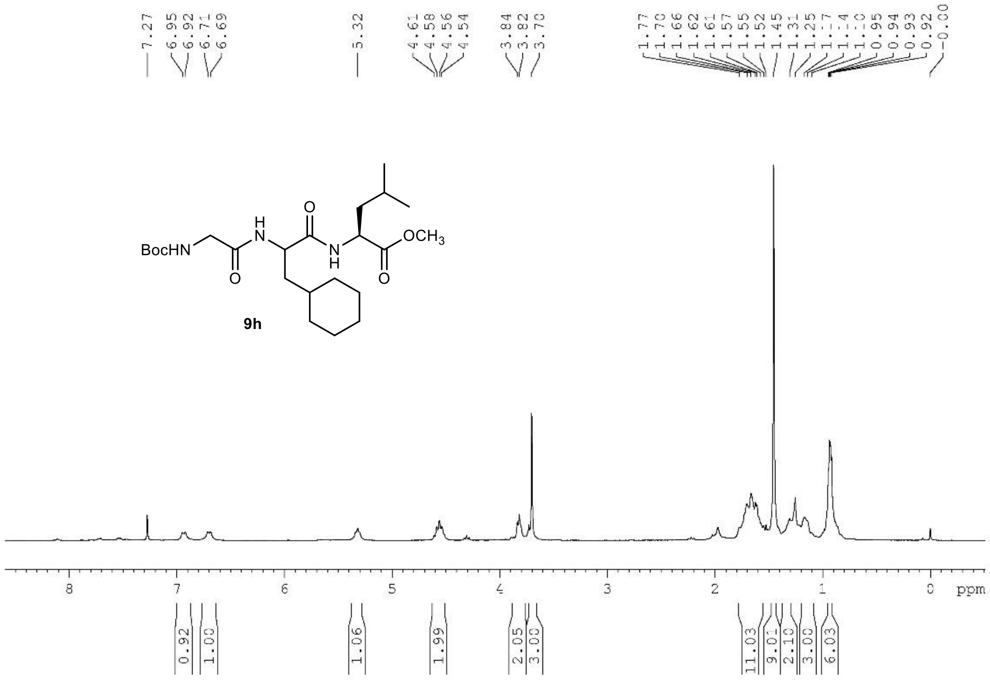

Supplementary Figure 97. ^{1}H NMR spectrum of compound **9h.**

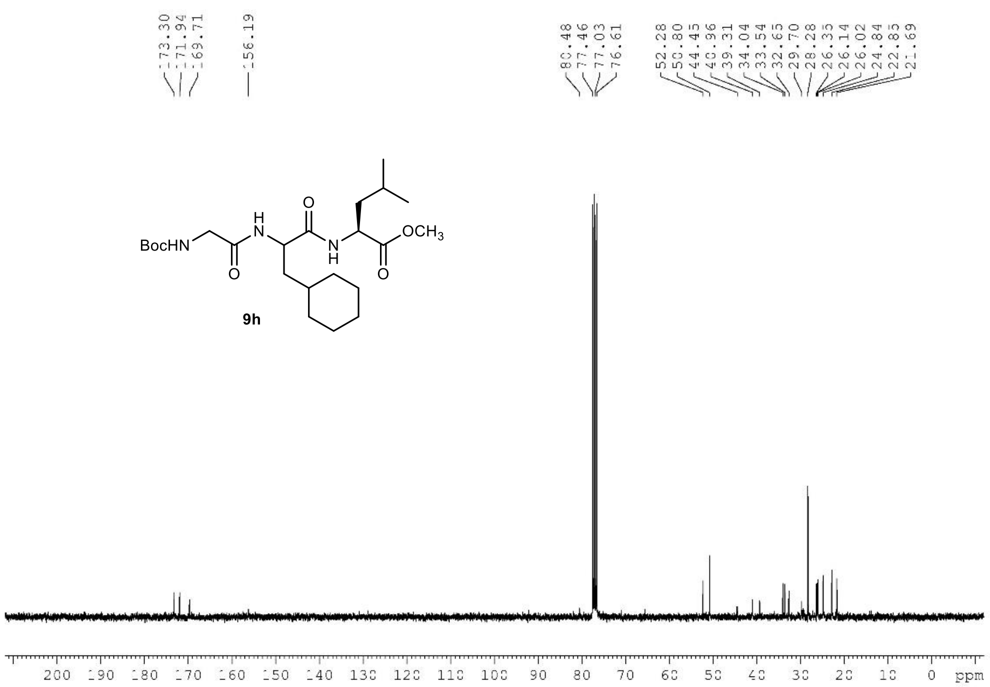

Supplementary Figure 98. ^{13}C NMR spectrum of compound **9h.**

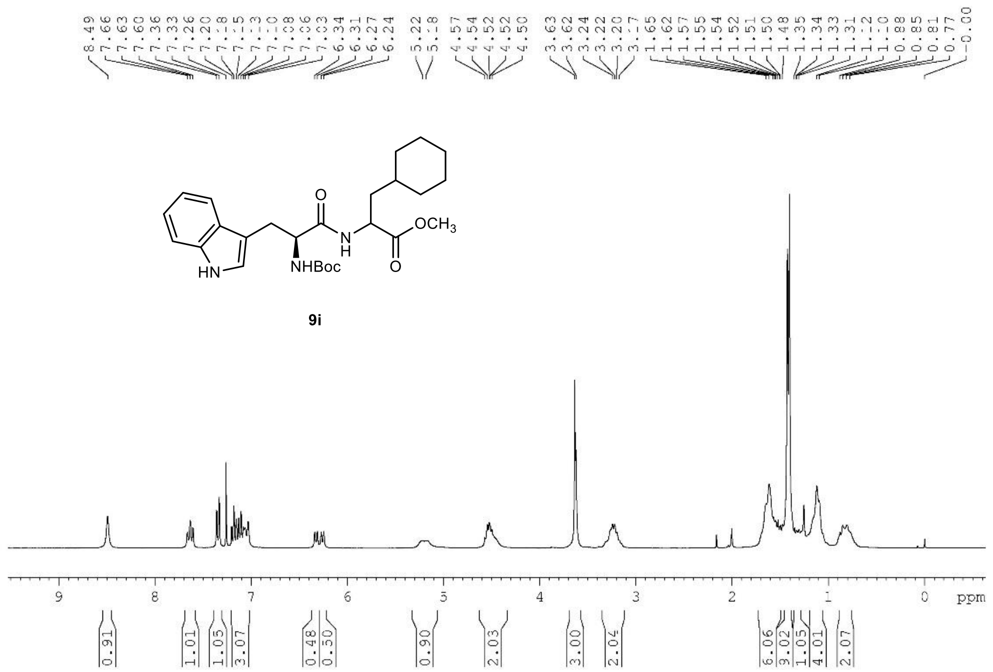

Supplementary Figure 99. ^{1}H NMR spectrum of compound **9i.**

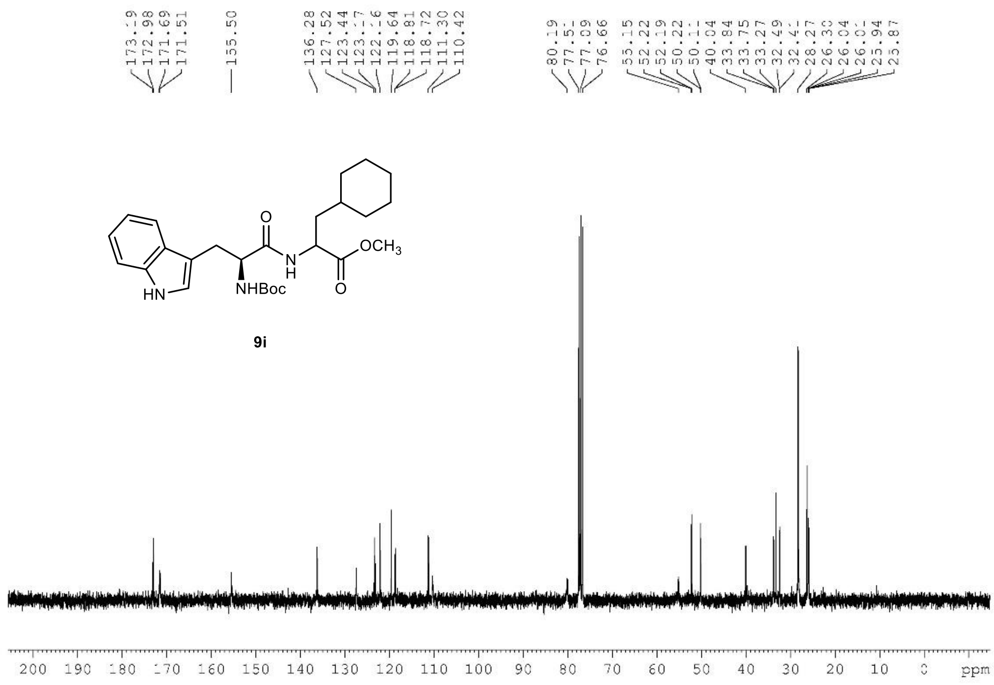

Supplementary Figure 100. ^{13}C NMR spectrum of compound **9i.**

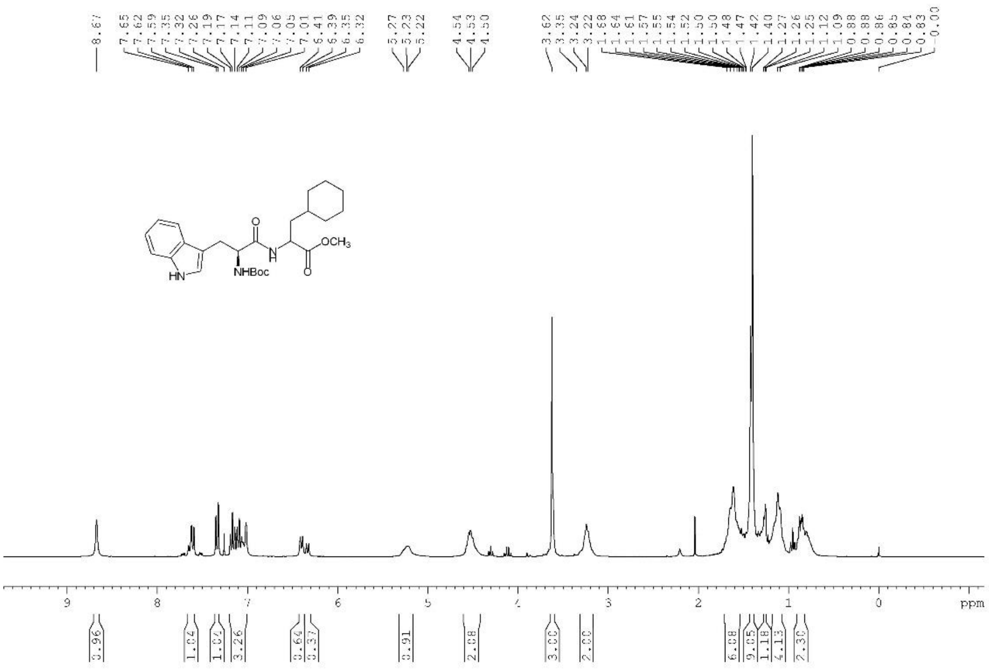

Supplementary Figure 101. ^{1}H NMR spectrum of compound **9i** (Condition B).

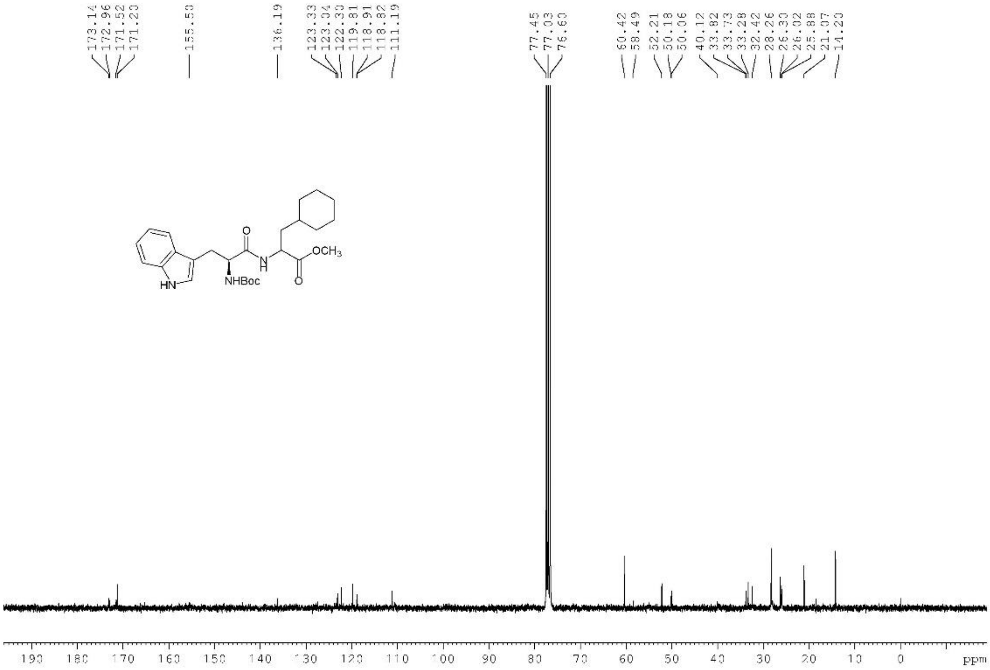

Supplementary Figure 102. ^{13}C NMR spectrum of compound **9i** (Condition B).

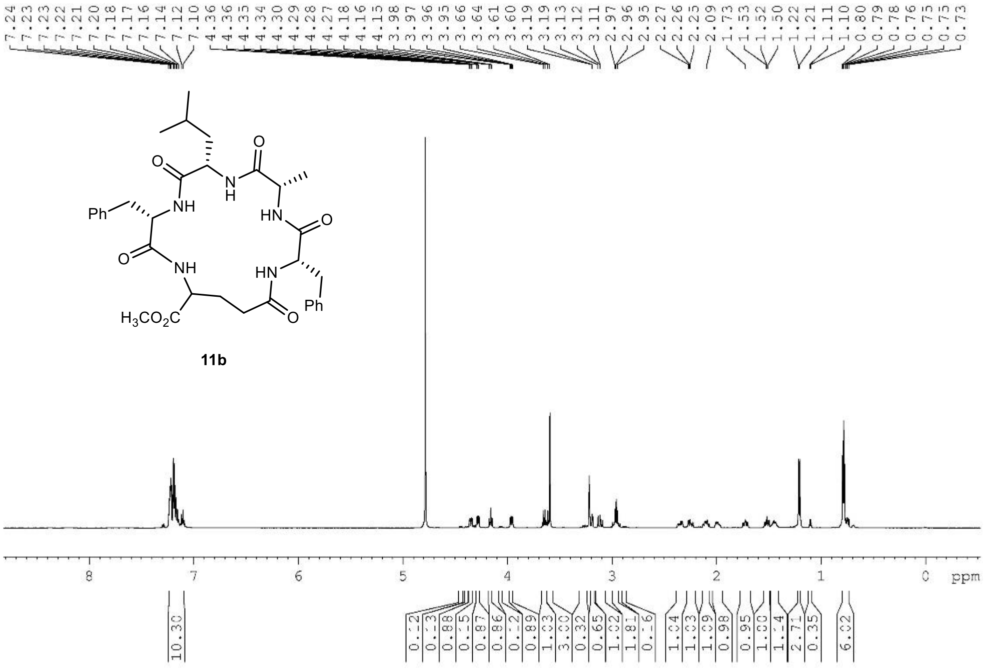

Supplementary Figure 103. ^{1}H NMR spectrum of compound **11b.**

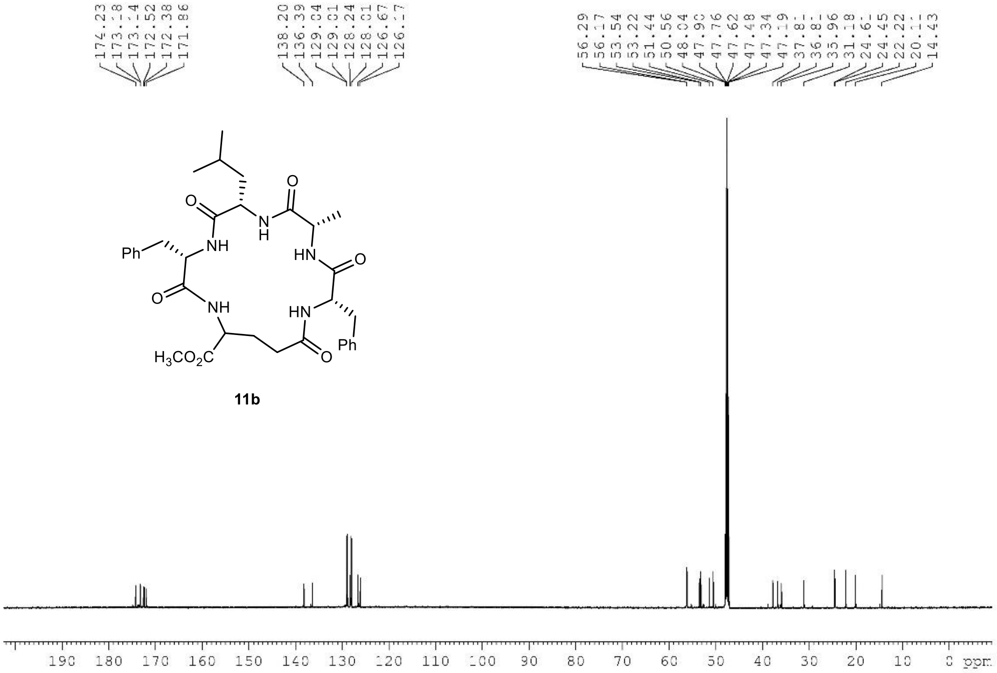

Supplementary Figure 104. ^{13}C NMR spectrum of compound **11b.**

7. Supplementary References

1. Wu, J., He, L., Noble, A. & Aggarwal, V. K. Photoinduced deaminative borylation of alkylamines. *J. Am. Chem. Soc*. **140**, 10700-10704 (2018).
2. Yue, H. et al. Nickel-catalyzed C-N bond activation: activated primary amines as alkylating reagents in reductive cross-coupling. *Chem. Sci*. **10**, 4430-4435 (2019).
3. Plunkett, S., Basch, C. H., Santana, S. O. & Watson, M. P. Harnessing alkylpyridinium salts as electrophiles in deaminative alkyl-alkyl cross-couplings. *J. Am. Chem. Soc*. **141**, 2257-2262 (2019).
4. Ociepa, M., Turkowska, J. & Gryko, D. Redox-activated amines in C(sp^3)-C(sp) and C(sp^3)-C(sp^2) bond formation enabled by metal-free photoredox catalysis. *ACS Catal*. **8**, 11362-11367 (2018).
5. Wang, C. et al. Visible-light-driven, copper-catalyzed decarboxylative C(sp^3)-H alkylation of glycine and peptides. *Angew. Chem. Int. Ed*. **57**, 15841-15846 (2018).
6. Chapmana, C. J., Hargravea, J. D., Bish, G. & Frosta, C. G. Peptide modification through site-selective residue interconversion: application of the rhodium-catalysed 1,4-addition of aryl siloxanes and boronates. *Tetrahedron* **64**, 9528-9539 (2008).
7. Chen, H. et al. Synthesis of α,α-difluorinated phosphonate pSer/pThr mimetics via rhodium-catalyzed asymmetric hydrogenation of β-difluorophosphonomethyl α-(acylamino)acrylates. *Org. Lett.* **20**, 3278-3281 (2018).
8. Wu, L., Wang, F., Chen, P. & Liu, G. Enantioselective construction of quaternary all-carbon centers via copper-catalyzed arylation of tertiary carbon-centered radicals. *J. Am. Chem. Soc.* **141**, 1887-1892 (2019).
9. Yin, H. et al. Stereoselective and divergent construction of β-thiolated/selenolated amino acids via photoredox-catalyzed asymmetric Giese reaction. *J. Am. Chem. Soc.* **142**, 14201-14209 (2020).
10. Kim, I., Im, H., Lee, H. & Hong, S. N-Heterocyclic carbene-catalyzed deaminative cross-coupling of aldehydes with Katritzky pyridinium salts. *Chem. Sci.* **11**, 3192-3197 (2020).
11. Li, Y. et al. Rhodium(III)-Catalyzed annulative carbooxygenation of 1,1-disubstituted alkenes triggered by C-H activation. *Chem. Eur. J.* **23**, 7453-7457 (2017).
12. Wu, J., Grant, P. S., Li, X., Noble, A. & Aggarwal, V. K. Catalyst-free deaminative functionalizations of primary amines by photoinduced single-electron transfer. *Angew. Chem. Int. Ed.* **58**, 5697-5701 (2019).
13. Wang, C. et al. Visible-light-promoted C(sp^3)-H alkylation by intermolecular charge transfer: preparation of unnatural α-amino acids and late-stage modification of peptides. *Angew. Chem. Int. Ed.* **59**, 7561-7466 (2020).
14. Kandukuri, S. R. et al. X-Ray characterization of an electron eonor-acceptor complex that drives the photochemical alkylation of indoles. *Angew. Chem. Int. Ed.* **54**, 1485-1489 (2015).
